# Expansion of the Stereochemical Space of Triterpenes by Mining Noncanonical Oxidosqualene Cyclases Across the Diversity of Green Plants

**DOI:** 10.1021/jacs.4c16956

**Published:** 2025-03-14

**Authors:** Samuel Edward Hakim, Shenyu Liu, Ronja Herzog, Ahmed Arafa, Jan de Vries, Gerald Dräger, Jakob Franke

**Affiliations:** a Centre of Biomolecular Drug Research, Leibniz University Hannover, Schneiderberg 38, Hannover 30167, Germany; b Institute of Botany, Leibniz University Hannover, Herrenhäuser Str. 2, Hannover 30419, Germany; c Pharmacognosy Department, Faculty of Pharmacy, Tanta University, Tanta 31527, Egypt; d Department of Applied Bioinformatics, Institute for Microbiology and Genetics, University of Göttingen, Goldschmidtstr. 1, Göttingen 37077, Germany; e Department of Applied Bioinformatics, Campus Institute Data Science (CIDAS), University of Göttingen, Goldschmidtstr. 1, Göttingen 37077, Germany; f Department of Applied Bioinformatics, Göttingen Center for Molecular Biosciences (GZMB), University of Göttingen, Goldschmidtstr. 1, Göttingen 37077, Germany; g Institute of Organic Chemistry, Leibniz University Hannover, Schneiderberg 1B, Hannover 30167, Germany

## Abstract

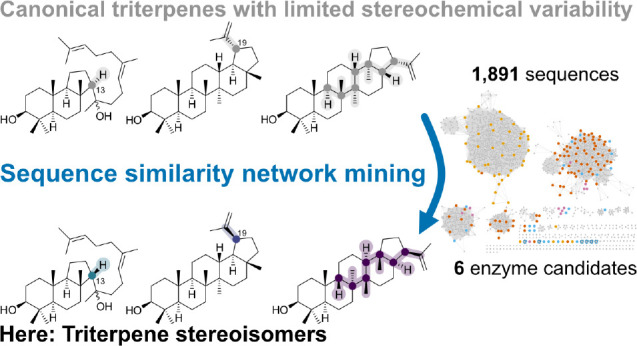

Triterpenoids and steroids are structurally complex polycyclic natural products with potent biological functions, for example, as hormones. In all eukaryotes, the carbon skeletons of these compounds are generated by oxidosqualene cyclases, which carry out a polycyclization cascade to generate four or five rings with up to nine stereogenic centers in a targeted manner. The tight stereochemical control of this cascade reaction severely limits the stereochemical space accessible by known oxidosqualene cyclases. Considering that naturally occurring hormone stereoisomers have markedly different biological activities, finding ways to produce stereoisomers of triterpenes would be highly desirable to open new avenues for developing triterpenoid and steroid drugs. Here, we present a plant kingdom-wide sequence mining approach based on sequence similarity networks to search for noncanonical oxidosqualene cyclases that might produce triterpene stereoisomers. From 1,891 oxidosqualene cyclase sequences representing the diversity of green plants, six candidates were selected for functional evaluation by heterologous production in *Nicotiana benthamiana*. Of these six candidates, three produced rare or previously inaccessible triterpene stereoisomers, namely, (3*S*,13*S*)-malabarica-17,21-diene-3β,14-diol, 19-*epi*-lupeol, and a previously unknown hopanoid stereoisomer that we call protostahopenol. Site-directed mutagenesis revealed key residues important for catalytic activity. The sequence similarity network mining strategy employed here will facilitate the targeted discovery of enzymes with unusual activity in higher organisms, which are not amenable to common genome mining approaches. More importantly, our work expands the accessible stereochemical space of triterpenes and represents the first step to the development of new triterpenoid-derived drugs.

## Introduction

**Figure 1:**
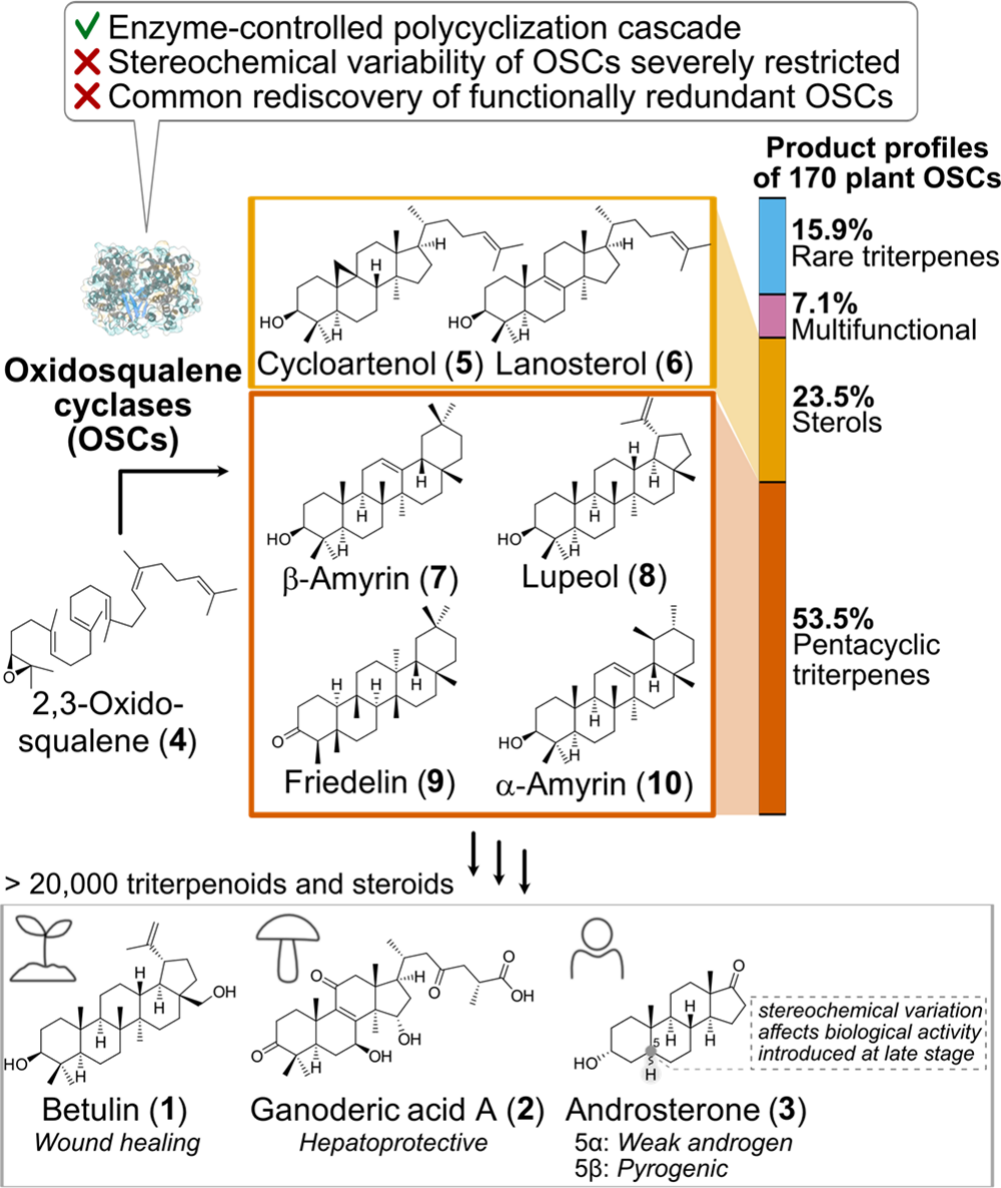
Triterpenoids and steroids (e.g., **1**–**3**) are widespread natural products in eukaryotes with potent biological functions and are derived from the simple precursor oxidosqualene (**4**) by enzymes called oxidosqualene cyclases (OSCs). Despite the fascinating biochemistry of the polycyclization cascades catalyzed by OSCs, these enzymes exhibit severely limited stereochemical variability and many are functionally redundant.^7^

Triterpenoids and steroids are famous natural products with important biological functions and industrial applications. Examples from the more than 20,000 representatives comprise the plant triterpenoid betulin (**1**),^1^ which is used in medicine to accelerate wound healing, ganoderic acid A (**2**) from fungi with hepatoprotective properties,^2^ and steroid hormones such as androsterone (**3**) in animals and humans.^3^ The complex polycyclic structures of this compound class as well as their enzymatic generation have intrigued chemists and biochemists for decades. In eukaryotes, the C_30_ carbon skeletons of triterpenoids and steroids are generated from the simple precursor 2,3-oxidosqualene (**4**) by enzymes termed oxidosqualene cyclases (OSCs).^4^ The polycyclization cascades catalyzed by OSCs belong to the most remarkable examples of enzymatic selectivity in Nature, generating typically four or five aliphatic rings and up to nine stereogenic centers in a controlled fashion (Figure 1).^4,5^ A major downside of the tight stereochemical control exerted by OSCs is that the stereochemical space accessible by these enzymes is severely restricted. Notably, the stereochemistry of polycyclic molecules can strongly affect their pharmacological properties, as even a single epimeric carbon atom can substantially alter the molecular shape and thus the interaction with drug targets.^6^ This is very well underlined by naturally occurring C5 epimers of androsterone (**3**) and related steroid hormones, which are generated at a late biosynthetic stage by 5α-reductase and 5β-reductase enzymes.^3,6^ While 5α-androsterone (**3a**) is a weak androgen,^3^ 5β-androsterone (also known as etiocholanolone) (**3b**) has pyrogenic (fever-inducing) properties in humans.^6^ Gaining access to triterpene skeletons with modified stereochemistry would therefore be highly attractive to develop new drugs based on triterpenoids or steroids.

Multiple studies have successfully achieved reprogramming of OSCs by protein engineering to generate carbon skeletons with altered constitution.^7−10^ However, stereoisomers rather than structural isomers are typically not accessible by this approach, as this would likely require larger alterations to the active sites of OSCs not achievable by a small set of mutations. Likewise, previous approaches to discover new OSCs have often only led to the rediscovery of enzymes with redundant or very similar functionality. Although 170 OSCs from plants had been characterized until 2021,^7^ the majority produced the same small set of compounds or showed insufficient selectivity (Figure 1). From these 170 plant OSCs, the most common products are the sterols cycloartenol (**5**) and lanosterol (**6**) (23.5%), and pentacyclic triterpenes such as β-amyrin (**7**), lupeol (**8**), friedelin (**9**), α-amyrin (**10**), and minor constitutional isomers (53.5%). Other OSCs are highly promiscuous (7.1%), producing up to 21 different products,^11^ and are therefore of limited value for downstream applications. Only the remaining 15.9% produce various rare triterpenes in a selective manner. The major cause why so many functionally redundant OSCs have been described is the default approach for finding new OSCs: Typically, when new plant species are investigated, the closest homologues of already characterized OSCs are prioritized. Owing to the ortholog conjecture, this entails a tendency to rediscover OSCs with known functions. A better approach to discover functionally more diverse OSCs has been the genome-wide systematic evaluation of all OSCs within a certain species.^8,12,13^ However, this strategy has been limited to single model plants such as *Arabidopsis thaliana*,^13^*Oryza sativa*,^8^ or *Avena strigosa*(12) so far and has also not yielded OSCs with stereochemical variability.

Considering that neither mutagenesis of OSCs nor searching for OSC homologues in a limited set of species granted access to stereoisomeric products, we envisioned that a new strategy to search for noncanonical enzymes would be required. We hypothesized that underexplored areas of the OSC sequence space have a much greater chance to yield enzymes with novel biochemical activity which might give access to rare or new triterpene stereoisomers. We now present here a strategy to search for OSCs with potentially novel function across the full diversity of green plants to overcome the dilemma of frequent rediscovery of functionally redundant OSCs. As a test sequence data set, we leveraged the data from the One Thousand Plant Transcriptomes initiative^14^ to provide a pool of 1,891 OSC sequences which was analyzed using sequence similarity networks. From six selected and tested candidates, three produced stereoisomers of triterpenes which have been difficult or impossible to access before. Site-directed mutagenesis was used to identify key residues responsible for catalytic activity. This search strategy will facilitate the targeted discovery of new biocatalysts from plants and animals in the future. Furthermore, our findings expand the stereochemical space of triterpenes and open the door to the development of new triterpenoid-derived drugs.

## Results

### Sequence Similarity Network Mining across the Diversity of Green Plants

**Figure 2:**
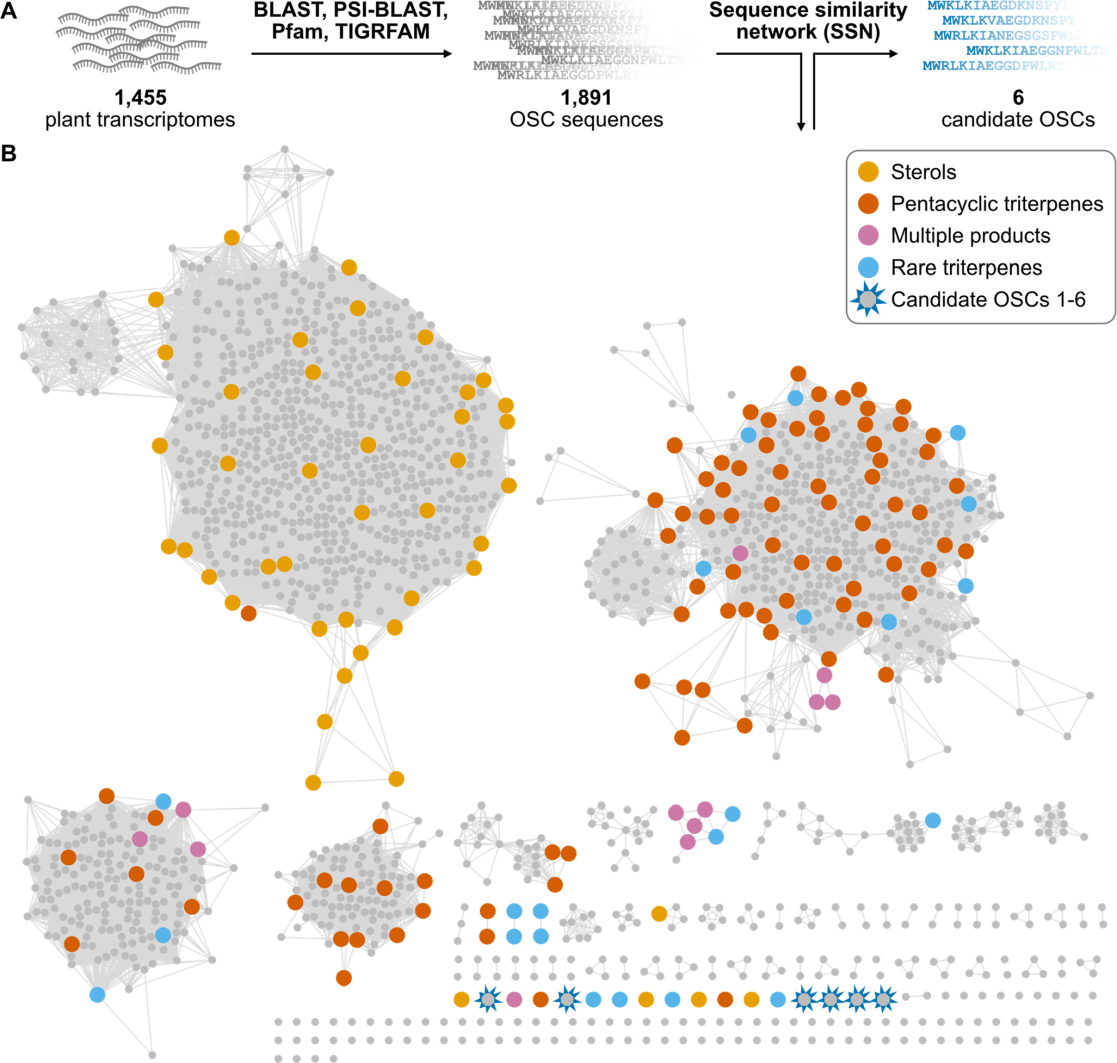
Plant kingdom-wide sequence similarity network (SSN) mining of oxidosqualene cyclases (OSCs). (A) Overview of mining strategy used in this work. (B) Sequence similarity network (SSN) of 1,891 OSCs from the whole plant kingdom (small gray circles) in comparison to 170 characterized OSCs (large colored circles). The color indicates the product category. An alignment score threshold of 360 was used. From this network, six candidates OSC1–6 were selected for functional evaluation.

The One Thousand Plant Transcriptomes initiative offers a comprehensive publicly available sequence data set that spans the diversity of green plants (Chloroplastida, formerly known as Viridiplantae).^14,15^ We therefore expected that it would provide a representative picture of the global sequence space of OSCs across more than billion-year-divergent plants and algae. To extract OSC sequences from this data set, we developed a custom script that combines two different search strategies (Figure 2A): 1) Sequence similarity to reported OSCs based on either BLAST or PSI-BLAST, with the latter being more sensitive toward distantly related sequences.^16^ As bait sequences, a set of 170 characterized reference OSCs compiled by Chen et al.^7^ was used. 2) Presence of OSC protein domains as judged by hidden Markov model (HMM) searches in comparison to the Pfam or TIGRFAM databases.^17−20^ Typical OSCs possess two protein domains, represented by the Pfam entries SQHop_N and SQHop_C, and one TIGRFAM domain, TIGR01787.

Various E-value cut-offs were evaluated for BLAST and HMM searches. The E-values of both search strategies had a strong effect on the number of shorter, likely fragmented OSC sequences; the number of full-length OSCs with ca. 750 amino acids, however, remained widely constant for BLAST E-values in the range e–20 to e–100 and HMM E-values in the range e–3 to e–30. All subsequent analyses were carried out with E-values of e–100 and e–30 for BLAST and HMM searches, respectively. The 170 reference OSCs show a narrow length distribution between 733 and 785 amino acids (Figure S1). We therefore excluded all hits with less than 700 amino acids, as these were considered to be fragmented sequences. In total, this search strategy resulted in 1,891 full-length OSC sequences for further analysis. Of these, 96% (i.e., 1,820) were identified by all tested search strategies (Figure S2). For 58, 11, and 2 OSCs, respectively, no SQHop_N, no SQHop_C and neither SQHop_N nor SQHop_C profiles were found at an E-value of e–30 (Figure S2). OSCs were found in all major clades of the plant kingdom (Figure S3, Table S1). Although we observed a general positive correlation between the number of sequenced species and the number of OSCs per clade, more recent clades exhibited a higher number of OSC per species (Figure S3). Overall, we concluded that our set of 1,891 OSC sequences would provide an adequate representation of OSC sequence space in the plant kingdom.

Next, we next wanted to visualize and screen the OSC sequence space to identify exotic OSCs that might possess unusual biochemical activity for further investigation. As phylogenetic trees become unwieldy for large numbers of sequences and offer limited possibilities for integrating metadata, we decided to employ sequence similarity networks (SSNs)^21,22^ using the web resource EFI-EST^23,24^ to investigate the relationship of sequences within this OSC data set. SSNs require careful finetuning of the alignment score threshold, to ensure that proteins with comparable function are correctly grouped together, but do not include proteins with different functions. Alignment score threshold optimization was guided by monitoring the position of the 170 characterized reference OSCs^7^ within the networks. Different alignment scores between 300 and 370 were compared. Lower alignment scores ≤ 350 led to undesired grouping of OSCs with different products, whereas higher alignment scores ≥ 370 led to a strong tendency to separate OSC clusters based on phylogeny rather than function (Figure S4, S5). An alignment score of 360 was considered to be an optimal compromise to group related catalytic function while limiting effects from phylogenetic distance.

The final SSN contained four major clusters, which contained 1,284 of the 1,891 extracted OSCs (68%), but 140 of 170 reference OSCs (82%) (Figure 2B). Again, this emphasized that previous strategies to identify new OSCs were strongly biased to reproduce known enzymology. The largest cluster contained almost all OSCs producing *chair*-*boat*-*chair* products (sterols such as cycloartenol (**5**), lanosterol (**6**), cucurbitadienol), and was well separated from OSCs producing *chair*-*chair*-*chair* products. This separation has also been observed previously in phylogenetic analyses.^4,25,26^ This sterol cluster contained OSCs from a broad range of plants, from nonvascular land plants to eudicots, whereas all other clusters were phylogenetically relatively closely confined (Figure S6). This might indicate a strong conservation of OSCs relevant for sterol biosynthesis and primary metabolism, resulting in limited sequence variation. Generally, apart from a few lineage-specific duplications, we observed a massive diversification of OSCs specific to flowering plants (angiosperms) (Figure S7), suggesting that chances to discover functionally novel OSCs would be highest among OSCs from angiosperms.

Most importantly, our OSC network contained 120 singletons that were not linked to any other OSC at the selected alignment score of 360. This set of singleton OSCs included 11 of the reference OSCs, some of them producing rare products such as a butyrospermol, achilleol B or taraxerol.^27−30^ The remaining 109 uncharacterized singleton OSCs were considered as prime candidates to represent new OSC functionality. Not surprisingly, we noted an overrepresentation of OSCs from ancient lineages in this singleton data set that is likely caused by phylogenetic distance rather than functional novelty (Figure S8). For this reason and due to the diversification of OSCs in angiosperms (Figure S7), we focused on OSCs from angiosperms. For a proof of concept, we selected six of these singleton OSCs, OSC1–6, representing different angiosperm families that have so far not been in the focus of triterpenoid research for functional characterization (Figure S9, Figure S10, Table S2). These are Aristolochiaceae (OSC1), Malvaceae (OSC2), Melianthaceae (OSC3), Hemerocallidaceae (OSC4), Asphodelaceae (OSC5), Escalloniaceae (OSC6) (Table S2).

### Characterization of OSC1–6

**Figure 3:**
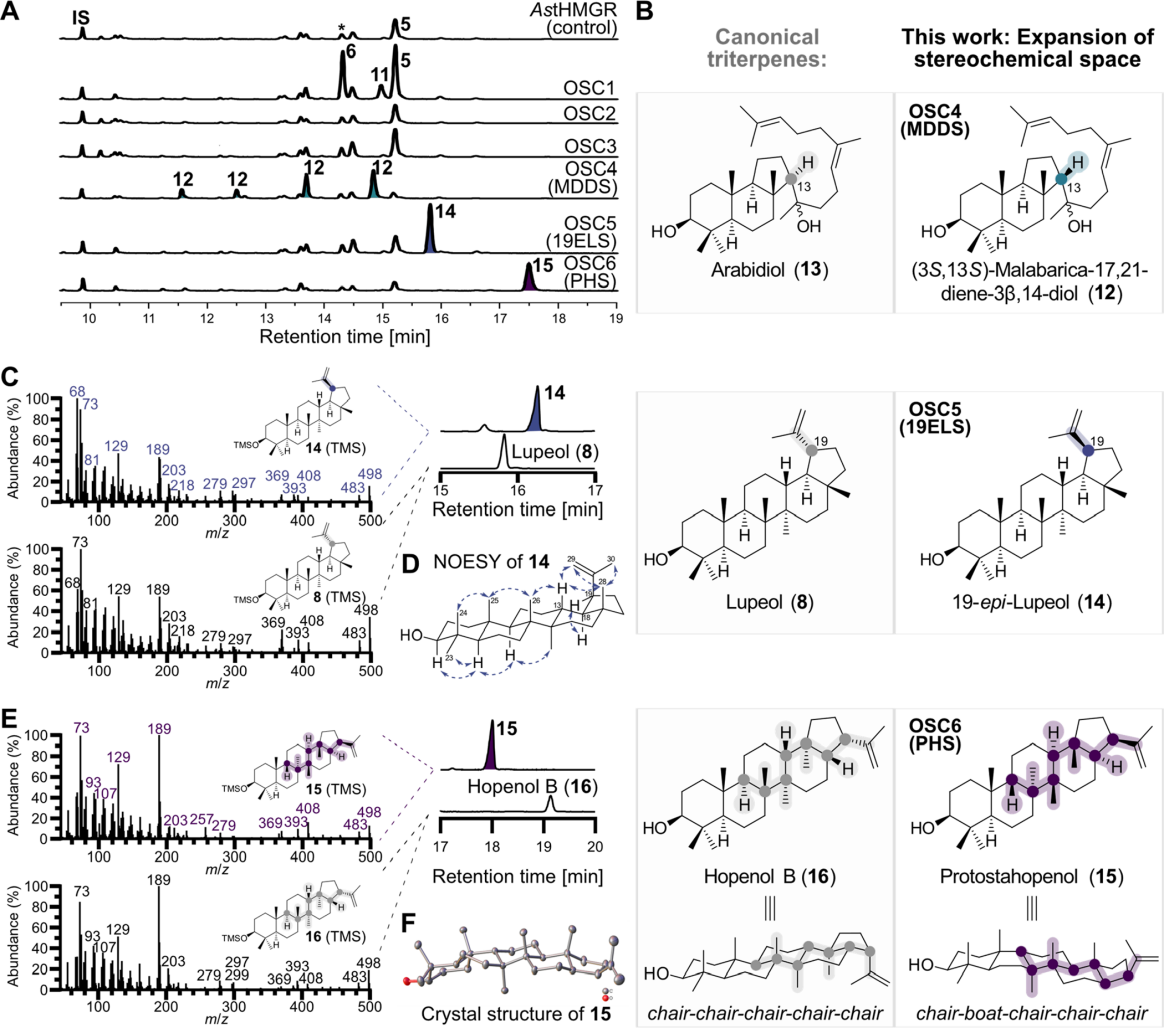
Functional characterization of OSC1–6, revealing formation of stereoisomeric triterpenes by OSC4 ((3*S*,13*S*)-malabarica-17,21-diene-3β,14-diol (**12**) synthase (MDDS)), OSC5 (19-*epi*-lupeol (**14**) synthase (19ELS)), and OSC6 (protostahopenol (**15**) synthase (PHS)). (A) GC-MS total ion current chromatograms of *OSC1–6* genes overexpressed in *N. benthamiana* together with an *Avena strigosa* truncated HMGR gene. * indicates a background peak that is different from the OSC1 product lanosterol (**6**) based on mass spectra comparison. (B) Side-by-side comparison of canonical triterpenes and their stereoisomers produced by OSC4–6 in this work. (C) Comparison of electron impact mass spectra of 19-*epi*-lupeol (**14**) and lupeol (**8**). (D) Key NOE correlations of 19-*epi*-lupeol (**14**) in support of the differing stereochemistry at C19. (E) Comparison of electron impact mass spectra of protostahopenol (**15**) and its diastereomer hopenol B (**16**). (F) Crystal structure of protostahopenol (**15**). Hydrogen atoms and lattice solvent molecules have been omitted for clarity. IS: Internal standard.

We tested the catalytic function of our six candidate OSCs 1–6 by transient gene expression in the plant host *Nicotiana benthamiana*, a common and reliable system for producing triterpenoids.^31−34^ A gene encoding a truncated, feedback-insensitive version of hydroxymethylglutaryl-CoA reductase (HMGR) from *Avena strigosa* was additionally coexpressed for increased supply of the OSC substrate oxidosqualene (**4**).^32^ After transient expression for 7 days, leaf extracts were analyzed by GC-MS to evaluate enzymatic activity (Figure 3A).

Overexpression of *OSC1* led to a new peak at 14.3 min that was absent in the negative control and an increase of a background peak at 15.2 min; by comparison to reference compounds, these were identified as lanosterol (**6**) and cycloartenol (**5**), respectively (Figure S11). A minor peak at 14.9 min showed a molecular ion with an increased *m*/*z* value of +14 compared to **5** and **6**; based on comparison of the mass spectrum to literature data,^35^ we assigned this product **11** as 24-methylenedihydrolanosterol, likely formed by unspecific background methylation of **6** in *N. benthamiana* (Figure S11C).^36^ OSCs 2 and 3 showed no detectable activity. Gratifyingly, OSC4, OSC5, and OSC6 each produced new peaks which did not match common OSC products by comparison of retention times and mass spectra to reference compounds. To characterize the products, transient expression was scaled up to 25–30 plants by vacuum infiltration,^37^ and the compounds were purified by successive rounds of chromatography for structure elucidation.

We obtained 0.5 mg/g dry weight isolated yield of triterpene **12**, the product of OSC4. Notably, pure **12** showed four peaks in GC-MS chromatograms, indicating that the compound is not stable during these conditions (Figure S12). The NMR spectrum contained four olefinic carbons in the range of 124–136 ppm, indicating the presence of two double bonds. In addition to the typical oxymethine carbon for C3 at 79.3 ppm, a second carbon with a similar downfield shift was observed at 76.2 ppm. This strongly suggested that product **12** was not a simple triterpene alcohol but a diol. Indeed, one of the peaks from GC-MS analysis also contained a signal at *m*/*z* 573, putatively corresponding to [M–CH_3_]^+^ of di-TMS-**12** (Figure S12). Using this partial information for a literature search, our compound **12** could be identified as the known triterpene (3*S*,13*S*)-malabarica-17,21-diene-3β,14-diol (Figure 3B, Figure S13, Table S3).^38^ The 13*R* epimer of compound **12** is known as arabidiol (**13**) (Figure 3B), and dedicated arabidiol synthases from plants have been described already.^38,39^ Although (3*S*,13*S*)-malabarica-17,21-diene-3β,14-diol (**12**) has been characterized before, it was only reported as a minor byproduct of a mutant squalene-hopene cyclase from bacteria^40^ and a mutant plant arabidiol cyclase.^38^ In contrast, OSC4 produces (3*S*,13*S*)-malabarica-17,21-diene-3β,14-diol (**12**), the C13 epimer of arabidiol (**13**), as its single main product; we therefore renamed OSC4 to (3*S*,13*S*)-malabarica-17,21-diene-3β,14-diol synthase (MDDS).

Next, we focused on OSC5 and isolated 1.6 mg/g dry weight isolated yield of triterpene **14**. NMR-based structure elucidation indicated a planar carbon backbone identical to the well-known triterpene lupeol (**8**). Indeed, electron impact mass spectra of lupeol (**8**) and our OSC5 product **14** were extremely similar, but the retention time of lupeol (**8**) clearly did not match our compound **14** (Figure 3C). Likewise, the NMR data was not consistent with authentic lupeol (**8**) (Table S4). Whereas^13^C chemical shifts in rings A-C were largely identical, carbons in the D and E ring showed substantial shift differences (Figure S14). We therefore suspected that **14** might be a stereoisomer of lupeol (**8**) in the E ring (Figure 3B). To elucidate the relative stereochemistry of **14**, we employed nuclear Overhauser effect spectroscopy (NOESY). The key correlations H18–H19, H13–H28, and H29/H30–H28 indicated that **14** was 19-*epi*-lupeol (Figure 3D); none of these correlations was visible in a NOE spectrum of lupeol (**8**) (Figure S15). We therefore renamed OSC5 to 19-*epi*-lupeol synthase (19ELS). 19-*epi*-Lupeol (**14**) is a rare triterpene reported only a single time (Table S5);^41^ to the best of our knowledge, it has not been observed as a product of any oxidosqualene cyclase before.

**Scheme 1:**
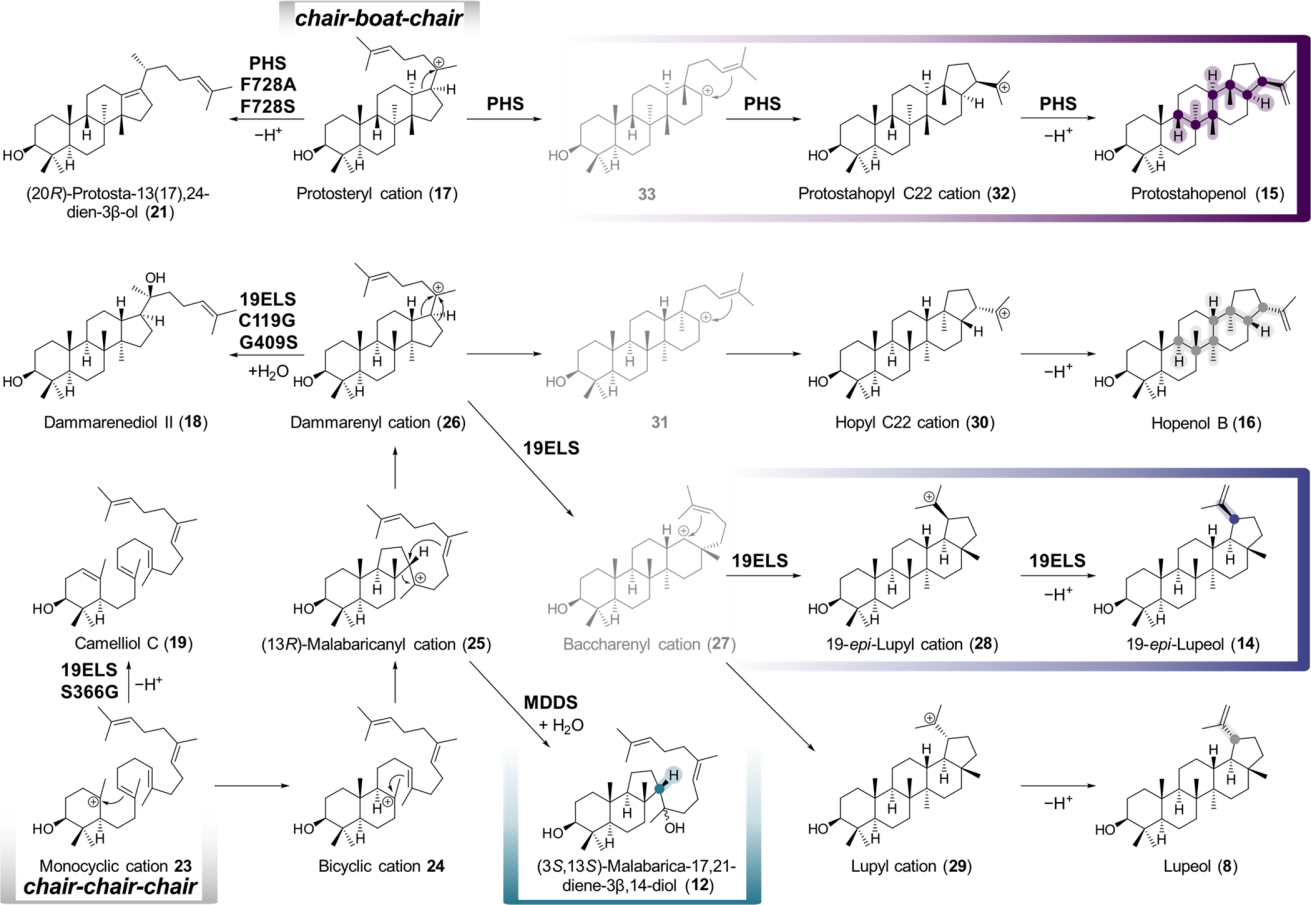
Proposed Mechanisms for Product Formation by MDDS, 19ELS, and PHS in Relation to More Common Triterpenes Initial cyclization of 2,3-oxidosqualene (**4**) can occur via a *chair*–*boat*–*chair* fold, leading to protosteryl cation (**17**), or via a *chair–chair–chair* fold to monocyclic cation **23** and later dammarenyl cation (**26**). The formation of pentacyclic cations likely proceeds in an asynchronous concerted manner rather than via the secondary carbocations shown in gray.^53^

Lastly, we turned our attention to OSC6 and obtained 3.4 mg/g dry weight isolated yield of triterpene **15** for NMR-based structure elucidation. We identified a planar skeleton matching to the rare triterpene hopenol B (**16**) (Figure S16),^12^ but again chemical shifts of our compound **15** were not in agreement with previously published data (Figure S17, Table S6).^12^ Expression of the recently reported *Aquilegia coerulea* hopenol B synthase gene^12^ in *N. benthamiana* confirmed that our compound **15** had a highly similar mass spectrum but different retention time compared with hopenol B (**16**) (Figure 3E). To elucidate the stereochemistry of our hopene-like compound **15**, we successfully obtained single crystals by slow evaporation from a dichloromethane-methanol (1:2) solution at 4 °C and solved its crystal structure, revealing an unexpected *chair-boat-chair* fold of the ABC rings (Figure 3F, Figure S18). This stereochemistry was also supported by independent NOESY analysis (Figure S16). Hopenes are widespread and crucial membrane components particularly in bacteria and are characterized by a complete *chair–chair–chair–chair–chair* fold, leading to extremely stable compounds.^42^ In sharp contrast, compound **15** combines the hopene skeleton with a *boat* conformation in the B ring typical for protosteryl cation (**17**) (Figure 3B, Scheme 1). Compound **15** was therefore named protostahopenol, and OSC6 was named protostahopenol synthase (PHS). Protostahopenol (**15**) is not known as a natural product, but a derivative has been found in plants before.^43,44^ Taken together, three of the six tested OSCs identified by our sequence similarity network mining approach yielded stereoisomers of common triterpenes (Figure 3B).

### Site-Directed Mutagenesis Reveals Key Residues

**Figure 4:**
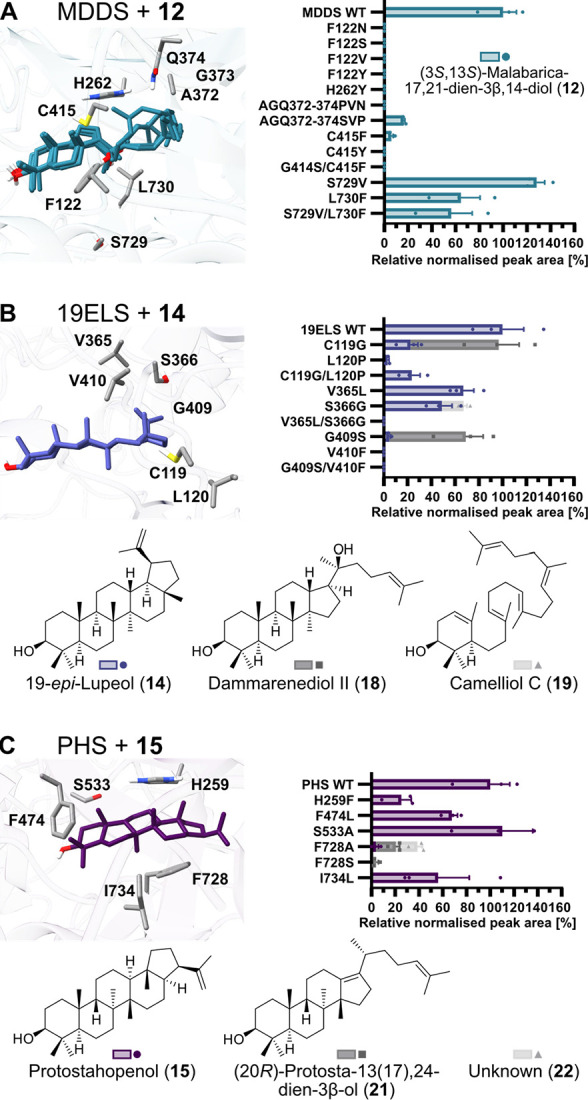
Site-directed mutagenesis of MDDS (OSC4), 19ELS (OSC5), and PHS (OSC6) to reveal key active site residues relevant for catalytic activity. Active site residues selected for mutagenesis, product profiles, and structures of mutant products of MDDS (A), 19ELS (B), and PHS (C) are shown. Protein structures are AlphaFold2 models of OSCs with docked products. Product profiles of mutants are provided as relative peak areas normalized by peak area of internal standard and dry weight of samples in comparison to the wildtype (WT) enzyme. Bar plots show mean ± SEM and data points of three biological replicates (i.e., three different infiltrated *N. benthamiana* plants).

Considering the unusual biochemical activity of OSC4–6, we next wanted to understand which active site residues are involved in product formation. As OSCs are integral membrane enzymes that are difficult to purify and crystallize, we generated AlphaFold2 models^45^ instead. OSC products **12**, **14**, and **15** were then docked into the active sites of our OSC structural models with AutoDock Vina (Figure 4).^46,47^

The structural models of the OSCs and docking poses were in very good agreement with a crystal structure of human lanosterol synthase in complex with its product lanosterol (**6**) (Figure S19), suggesting that our prediction was reliable enough to select active site residues for site-directed mutagenesis. For (3*S*,13*S*)-malabarica-17,21-diene-3β,14-diol (**12**), both C14 epimers were docked, and five possible docking poses with similar energies were obtained in total due to the long flexible side chain of the tricyclic compound. We used a combination of multiple sequence alignments and analysis of the structural models to prioritize residues and mutations that might be relevant for the unusual biochemical activity of MDDS, 19ELS, and PHS (Figure S20–S22). All mutants were tested by transient expression in *N. benthamiana* in comparison with the wildtype enzymes.

For MDDS, 12 mutants were generated for the eight residues F122, H262, A372, G373, Q374, C415, S729, and L730, based on a multiple sequence alignment of MDDS with OSCs that produce arabidiol or other triterpenes that are only partially cyclized (Figure 4A, Figure S20). The mutants S729V and L730F alone or in combination only had a minor effect on overall productivity. In contrast, all other mutants showed a strong loss of total activity and no formation of other triterpenes (Figure 4A, Figure S23A).

For 19ELS, we tested nine mutants of residues C119, L120, V365, S366, G409, and V410, which are close to the epimeric position C19 and not conserved in standard lupeol synthases (Figure 4B, Figure S21). The mutants L120P and V410F as well as the double mutants G409S+V410F and C119G+L120P only showed a complete or at least strong loss of total activity (Figure 4B, Figure S23B). In contrast, mutants C119G and G409S produced less 19-*epi*-lupeol (**14**), but instead a new product **18**. We isolated the compound and confirmed by NMR spectroscopy that it was dammarenediol II (**18**) (Table S7). The mutant S366G also produced less **14** and another new product **19**. By isolation and NMR-based structure elucidation we identified this compound as the monocyclic triterpene camelliol C (**19**) (Table S8). These results show that C119 and G409 are important for the final formation of the E ring, whereas S366 is connected to the initial cyclization cascade. In mutants S366G and G409S, an additional unknown compound **20** was observed (Figure S23). Compound **20** coeluted with another peak under our GC-MS conditions; our isolation efforts also failed as the compound could not be separated from residual 19-*epi*-lupeol (**14**). Lupeol (**8**) was not observed in any of the mutants, and hence no simple functional swap of 19ELS to a lupeol synthase could be achieved with the tested set of mutants.

For PHS, six mutants of the five residues H259, F474, S533, F728, and I734 were evaluated that reflect differences to reported hopenol B synthases (Figure 4C, Figure S22).^12^ Whereas mutations H259F, F474L, S533A, and I734L only had a moderate or weak effect on the overall productivity, the mutations F728A and F728S led to a strong or complete reduction of protostahopenol (**15**) levels, respectively, and formation of a new product **21** (Figure 4C, Figure S23C). Compound **21** was identified as the known compound (20*R*)-protosta-13 (17),24-dien-3β-ol (**21**) by isolation and NMR spectroscopy (Table S9).^48^ Mutant F728A also produced an unknown compound **22** related to the tetracyclic triterpene parkeol (Figure S24). These results strongly support our hypothesis that protostahopenol (**15**) is indeed biosynthetically derived from protosteryl cation (**17**) and underline the importance of PHS residue F728 for the formation of the E ring of protostahopenol (**15**).

## Discussion

The characterization of OSC4–6 which form the rare or previously inaccessible triterpene stereoisomers (3*S*,13*S*)-malabarica-17,21-diene-3β,14-diol (**12**), 19-*epi*-lupeol (**14**), and protostahopenol (**15**) demonstrates that sequence similarity network mining is a powerful approach to discover unusual oxidosqualene cyclases from plants. The discovery of MDDS, 19ELS, and PHS opens up new regions of stereochemical space of triterpenoids which are now accessible by biocatalytic approaches. We anticipate that coupling of these OSCs with tailoring enzymes such as cytochrome P450 monooxygenases^49^ by combinatorial biosynthesis^33,50,51^ can lead to the generation of new bioactive triterpenoids that could not be produced enzymatically or synthetically before. We propose the following mechanistic routes for the formation of these compounds (Scheme 1):

The formation of (3*S*,13*S*)-malabarica-17,21-diene-3β,14-diol (**12**) likely proceeds via monocyclic cation **23**, bicyclic cation **24**, the tricyclic (13*R*)-malabaricanyl cation **25**, and final cation quenching with water, as suggested by others.^26,38,52^

For 19-*epi*-lupeol (**14**), we propose that first the tetracyclic cation dammarenyl cation (**26**) is formed. Ring expansion and cyclization via baccharenyl cation (**27**) or an asynchronous concerted reaction^53^ could lead to 19-*epi*-lupyl cation (**28**) instead of the common lupyl cation (**29**), to give rise to 19-*epi*-lupeol (**14**) instead of lupeol (**8**) after loss of a proton. To achieve C19 epimer formation, the terminal isobutenyl moiety would need to exist in a flipped orientation compared to lupeol (**8**) formation to make the opposite face of the double bond accessible (Figure S25). The formation of the E ring can be interrupted by 19ELS mutations C119G and G409S, leading to dammarenediol II (**18**), whereas mutation S366G interferes with the cyclization at an even earlier stage to give rise to camelliol C (**19**) from monocyclic cation **23**.

In the case of PHS, we propose a more initial divergence compared to hopene biosynthesis. For hopenol B (**16**), it is proposed that dammarenyl cation (**26**), formed by *chair–chair–chair* cyclization, undergoes a ring expansion and cyclization to hopyl C22 cation (**30**).^12^ This could proceed via secondary carbocation **31** analogous to baccharenyl cation (**27**) or in an asynchronous concerted reaction. For protostahopenol (**15**), we propose that protosteryl cation (**17**), formed by *chair-boat-chair* cyclization, would be the starting point instead of dammarenyl cation (**26**). The involvement of protosteryl cation (**17**) is also supported by the formation of (20*R*)-protosta-13(17),24-dien-3β-ol (**21**) from PHS mutants F728A and F728S. Ring expansion and cyclization of protosteryl cation (**17**) analogous to the hopenol B (**16**) pathway would then lead to protostahopyl C22 cation (**32**), either via secondary carbocation **33** or in an asynchronous concerted way. Final proton loss of the C22 carbocations **32** and **30** would then give rise to protostahopenol (**15**) and hopenol B (**16**), respectively.

By site-directed mutagenesis, we identified multiple key amino acids which are important for the overall biochemical activity of OSC4–6. However, with the mutants tested, we could not achieve a switch in stereochemistry to more canonical products, such as converting 19ELS to a lupeol synthase, or PHS to a hopenol B synthase or isoarborinol synthase. A possible explanation might be that we actively selected OSCs that are very distant from typical OSCs. For example, none of the 170 reference OSCs shares more than 61% amino acid sequence identity with 19ELS (Figure S10). Therefore, the evolutionary trajectory between OSC4–6 and more common OSCs likely depends on multiple mutation events that need to be combined for successful reprogramming. In contrast to other studies where single amino acid mutations of OSCs led to a complete functional swap,^7,9,10^ more efforts will be necessary to understand the structural and functional determinants that enable OSC4–6 to produce stereoisomers of more common triterpenes. Most importantly, a better coverage of the full OSC sequence space will be required to determine how distant OSCs accumulated sets of mutations to reach their unusual biochemical functions.

We could not identify obvious reasons why OSC2 and OSC3 did not show any detectable activity. To make sure that the sequences from the One Thousand Plant Transcriptomes initiative are reliable, we analyzed the read coverage underlying the *de novo* transcriptome assemblies (Figure S26). The coding sequence of *OSC3* was overall well supported, while for *OSC2* the read support in the middle of the transcript was weak (Figure S26). We also checked whether the conserved protein motifs of OSCs^7,54^ exhibited any anomalies (Figure S27). For OSC3, no unusual variants were found; for OSC2, the MWCYCR motif contained an unusual C→Y mutation that is not seen for any of the 170 reference OSCs^7^ (Figure S27). Nonetheless, OSC3 and – with minor limitations – OSC2 appear overall like *bona fide* OSCs in terms of length, sequence homology and protein motifs.

Our work demonstrates that sequence similarity network mining can be a powerful approach to rapidly discover enzymes with novel or unusual function in plants and generally across the eukaryote tree of life. Whereas genome mining is a common approach for discovering new natural products and enzymology in bacteria and fungi,^55^ similar strategies in plants have been lagging behind due to the larger genome size and complexity of plants.^56^ Only over the past decade, more phylogenetically informed efforts to systematically sequence the biodiversity of plants and algae have come to fruition.^57^ Now, phylodiverse plant genome and transcriptome data sets await to be tapped to accelerate the discovery of biosynthetic enzymes from plants.^58^

While several breakthrough studies already demonstrated that mining sequence data from plants is a promising approach,^56,59,60^ previous studies have remained mostly restricted to individual species,^59^ genera^8^ or families.^61^ Successful recent examples of this approach include genome-wide searches of OSCs from quinoa,^62^ tea,^63^ and rice.^64^ Here, we demonstrate that unusual biochemistry can be found by searching large-scale sequencing data across the diversity brought forth by hundreds of millions of years of plant and algal evolution. Nonetheless, there are still limitations that need to be overcome in the future. While the transcriptome data from the One Thousand Plant Transcriptomes initiative provides an excellent and easily accessible starting point, it bears two major problems: First, the sequencing quality based on short read technology is not always optimal, resulting in a relatively high number of fragmented or potentially misassembled sequences. Second, only vegetative tissues were sampled, but many triterpenoids come from other tissues such as roots or bark.^65−67^ It is therefore likely that many more novel OSCs could be discovered with more comprehensive sequencing data, to possibly enhance the stereochemical space of triterpenes and sterols further. This could also be combined with synteny network analyses of OSC flanking genes^68^ to find tailoring enzymes acting on such unusual scaffolds. We propose that, with a further increase of available sequence data and particularly chromosome-level genome assemblies, our sequence similarity network mining strategy will become even more powerful in the near future.

## Conclusions

We presented here a strategy for mining large sequence data sets across the diversity of plants with sequence similarity networks. This led to the discovery of three oxidosqualene cyclases – out of six tested candidates – capable of producing stereoisomers of more common triterpenes. These enzymes produce the rare triterpene (3*S*,13*S*)-malabarica-17,21-diene-3β,14-diol (**12**), 19-*epi*-lupeol (**14**), which was so far not accessible by oxidosqualene cyclases, and the previously undescribed compound protostahopenol (**15**). As such, our work demonstrates that sequence similarity network mining is a promising strategy to facilitate the discovery of enzymes with novel functions in plants and other eukaryotes. This approach reduces the risk of rediscovering functionally redundant enzymes and enabled the discovery of oxidosqualene cyclases that expand the stereochemical space of triterpenes.

## Supporting Information Available

Experimental details, materials, compound purification and characterization details, mutagenesis details, spectra, and sequences (PDF)

## Abbreviations

19ELS: 19-*epi*-lupeol (**14**) synthase
HMGR: hydroxymethylglutaryl-CoA reductase
MDDS: (3*S*,13*S*)-malabarica-17,21-diene-3β,14-diol (**12**) synthase
NOESY: nuclear Overhauser effect spectroscopy
OSC: oxidosqualene cyclase
PHS: protostahopenol (**15**) synthase
