# Supplementary material for: Expansion of the Stereochemical Space of Triterpenes by Mining Noncanonical Oxidosqualene Cyclases Across the Diversity of Green Plants
Source: J Am Chem Soc. 2025 Mar 14;147(12):10320–30. doi: 10.1021/jacs.4c16956 (PMC11951148; doi:10.1021/jacs.4c16956)
Supplement: Supplementary file 1 — ja4c16956_si_001.pdf [file ja4c16956_si_001.pdf]

Supporting Information for

Expansion of the Stereochemical Space of Triterpenes by Mining Noncanonical Oxidosqualene Cyclases Across the Diversity of Green Plants

Samuel Edward Hakim[a], Shenyu Liu[a], Ronja Herzog[a], Ahmed Arafa[b][c], Jan de Vries[d][e][f], Gerald Dräger[g] and Jakob Franke[a][b]*

[a] Centre of Biomolecular Drug Research, Leibniz University Hannover, Schneiderberg 38, 30167 Hannover (Germany)

[b] Institute of Botany, Leibniz University Hannover, Herrenhäuser Str. 2, 30419 Hannover (Germany)

[c] Pharmacognosy Department, Faculty of Pharmacy, Tanta University, 31527 Tanta (Egypt)

[d] Department of Applied Bioinformatics, Institute for Microbiology and Genetics, University of Göttingen, Goldschmidtstr. 1, 37077 Göttingen (Germany)

[e] Department of Applied Bioinformatics, Campus Institute Data Science (CIDAS), University of Göttingen, Goldschmidtstr. 1, 37077 Göttingen (Germany)

[f] Department of Applied Bioinformatics, Göttingen Center for Molecular Biosciences (GZMB), University of Göttingen, Goldschmidtstr. 1, 37077 Göttingen (Germany)

[g] Institute of Organic Chemistry, Leibniz University Hannover, Schneiderberg 1B, 30167 Hannover (Germany)

* To whom correspondence should be addressed: jakob.franke@botanik.uni-hannover.de

Table of Contents

Table of Contents 2
Experimental Procedures 4
General chemical methods 4
Mining of OSC sequences and sequence similarity network analysis 4
Cloning and transient expression of candidate oxidosqualene cyclase genes in *Nicotiana benthamiana* 4
Metabolite extraction 5
GC-MS analysis 5
Purification of OSC products 5
Analytical data 7
X-ray diffraction measurements 8
Bioinformatics analyses, docking, and selection of targets for site-directed mutagenesis 8
Site-directed mutagenesis 8
Supplementary References 73

Figures

Figure S1. Amino acid sequence length distributions of potential OSCs found by different search strategies in comparison to the 170 reference OSCs collected by Chen *et al.*[3] 9
Figure S2. Venn diagram of OSC count identified by different search strategies (BLASTP, PSI-BLAST, Pfam (SQHop_N and SQHop_C profiles) and TIGRFAM (TIGR01787)). 10
Figure S3. Phylogenetic distribution of oxidosqualene cyclases (OSCs) identified from the One Thousand Plant Transcriptomes dataset in comparison to the number of sequenced species per clade. 11
Figure S4. Sequence similarity network of 1,891 OSCs and 170 reference OSCs at a sequence alignment threshold of E 350. 12
Figure S5. Sequence similarity network of 1,891 OSCs and 170 reference OSCs at a sequence alignment threshold of E 370. 13
Figure S6. Sequence similarity network of 1,891 OSCs and 170 reference OSCs highlighting their phylogenetic position. 14
Figure S7. Maximum likelihood phylogenetic tree of OSCs sampled across streptophyte diversity 15
Figure S8. Phylogenetic distribution of 109 singleton OSCs identified from One Thousand Plant Transcriptomes dataset in relation to the total numbers of OSCs from these groups. 16
Figure S9. Maximum likelihood phylogenetic tree of OSC1-6, 170 reference OSCs from ref. [3], and three hopanoid synthases from ref. [10]. 17
Figure S10. Heatmap representation of the multiple sequence alignment of reference OSCs[3] and OSCs 1-6 underlying the phylogenetic tree in Figure S9. 18
Figure S11. Production of lanosterol (**6**) and cycloartenol (**5**) by OSC1 and of the *N. benthamiana* shunt product 24-methylenedihydrolanosterol (**11**). 19
Figure S12. Pure (3*S*,13*S*)-malabarica-17,21-diene-3β,14-diol (**12**) shows four peaks a, b, c, and d upon saponification, derivatisation, and GC-MS analysis 20
Figure S13. Key NMR correlations of (3*S*,13*S*)-malabarica-17,21-diene-3β,14-diol (**12**). 21
Figure S14. Position-specific ^{13}C NMR shift differences between 19-*epi*-lupeol (**14**) and lupeol (**8**) (C_6D_6). 22
Figure S15. Key NMR correlations of 19-*epi*-lupeol (**14**) in comparison with lupeol (**8**). 23
Figure S16. Key NMR correlations of protostahopenol (**15**). 24
Figure S17. Position-specific ^{13}C NMR shift differences between protostahopenol (**15**) and hopenol B (**16**). 25
Figure S18. ORTEP of protostahopenol (**15**) with ellipsoids drawn at the 50% probability level and hydrogens omitted for clarity. 26
Figure S19. AlphaFold2 models of MDDS (OSC4) (A), 19ELS (OSC5) (B) and PHS (OSC6) (C) with docked products (3*S*,13*S*)-malabarica-17,21-diene-3β,14-diol (**12**) (D), 19-*epi*-lupeol (**14**) (E), or protostahopenol (**15**), respectively, in comparison to the crystal structure of lanosterol synthase (1W6K) (grey) containing lanosterol (orange).[18] 27
Figure S20. Multiple sequence alignment of MDDS (OSC4), OSCs with a similar product spectrum, and selected representative OSCs 28
Figure S21. Multiple sequence alignment of 19ELS (OSC5) and OSCs with a similar product spectrum. 29
Figure S22. Multiple sequence alignment of PHS (OSC6) and OSCs with a similar product spectrum. 30
Figure S23. Detailed OSC mutagenesis results 31
Figure S24. Unknown compound **22** produced by PHS mutant F728A matches with a minor unknown byproduct of *Oryza sativa* parkeol synthase (OsPS)[11] in terms of retention time and mass spectrum. 32
Figure S25. Proposed mechanism for 19-*epi*-lupeol (**14**) formation involving a flipped orientation (highlighted in red) of the terminal isobutenyl moiety in comparison to lupeol (**8**) formation. 33
Figure S26. Read coverage of the *OSC2* and *OSC3* transcripts in the One Thousand Plant Transcriptomes dataset. 34
Figure S27. Comparison of conserved motifs[3,27] between the 170 reference OSCs[3] and OSC2/OSC3. 35
Figure S28. ^{1}H spectrum of (3*S*,13*S*)-malabarica-17,21-diene-3β,14-diol (**12**) ($CDCl_3$, 500 MHz, 298 K). 36
Figure S29. ^{13}C spectrum of (3*S*,13*S*)-malabarica-17,21-diene-3β,14-diol (**12**) ($CDCl_3$, 151 MHz, 298 K). 37
Figure S30. ^{1}H spectrum of 19-*epi*-lupeol (**14**) (C_6D_6, 500 MHz, 298 K). 38
Figure S31. ^{13}C spectrum of 19-*epi*-lupeol (**14**) (C_6D_6, 100 MHz, 298 K). 39
Figure S32. HSQC spectrum of 19-*epi*-lupeol (**14**) (C_6D_6, 500 MHz, 298 K). 40
Figure S33. HMBC spectrum of 19-*epi*-lupeol (**14**) (C_6D_6, 500 MHz, 298 K). 41
Figure S34. COSY spectrum of 19-*epi*-lupeol (**14**) (C_6D_6, 500 MHz, 298 K). 42
Figure S35. NOESY spectrum of 19-*epi*-Lupeol (**14**) (C_6D_6, 500 MHz, 298 K). 43
Figure S36. ^{1}H spectrum of protostahopenol (**15**) ($CDCl_3$, 600 MHz, 298 K). 44
Figure S37. ^{13}C spectrum of protostahopenol (**15**) ($CDCl_3$, 151 MHz, 298 K). 45
Figure S38. HSQC spectrum of protostahopenol (**15**) ($CDCl_3$, 600 MHz, 298 K). 46
Figure S39. HMBC spectrum of protostahopenol (**15**) ($CDCl_3$, 600 MHz, 298 K). 47
Figure S40. COSY spectrum of protostahopenol (**15**) ($CDCl_3$, 600 MHz, 298 K). 48
Figure S41. NOESY spectrum of protostahopenol (**15**) ($CDCl_3$, 600 MHz, 298 K). 49
Figure S42. ^{1}H spectrum of dammarenediol II (**18**) ($CDCl_3$, 600 MHz, 298 K). 50

Figure S43. ^{13}C spectrum of dammarenediol II (**18**) ($CDCl_3$, 151 MHz, 298 K) 51
Figure S44. ^{1}H spectrum of camelliol C (**19**) ($CDCl_3$, 400 MHz, 298 K) 52
Figure S45. ^{13}C spectrum of camelliol C (**19**) ($CDCl_3$, 100 MHz, 298 K). 53
Figure S46. ^{1}H spectrum of (20*R*)-protosta-13(17),24-dien-3β-ol (**21**) ($CDCl_3$, 600 MHz, 298 K). 54
Figure S47. ^{13}C spectrum for (20*R*)-protosta-13(17),24-dien-3β-ol (**21**) ($CDCl_3$, 151 MHz, 298 K). 55
Figure S48. ^{1}H spectrum of lupeol (**8**) (C_6D_6, 600 MHz, 298 K) 56
Figure S49. ^{13}C spectrum of lupeol (**8**) (C_6D_6, 151 MHz, 298 K). 57

Tables

Table S1. Phylogenetic group summary based on clades defined in One Thousand Plant Transcriptomes dataset.[2] 58
Table S2. List of six OSCs tested in this work 59
Table S3. NMR shifts of (3*S*,13*S*)-malabarica-17,21-diene-3β,14-diol (**12**) in comparison to literature.[35] 60
Table S4. NMR shifts of 19-*epi*-lupeol (**14**) in comparison to lupeol (**8**) (own measurements) and lupeol acetate (**34**) literature data (all in C_6D_6).[37] 61
Table S5. ^{13}C NMR shifts of 19-*epi*-lupeol (**14**) in comparison to literature data (all in $CDCl_3$).[39] 62
Table S6. NMR shifts of protostahopenol (**15**) in comparison to the stereoisomer hopenol B (**16**).[10] 63
Table S7. NMR shifts of dammarenediol II (**18**), product of 19ELS mutants C119G and G409S, in comparison to literature.[40] 64
Table S8. NMR shifts of camelliol C (**19**), product of 19ELS mutant S366G, in comparison to literature.[42] 65
Table S9. NMR shifts of (20*R*)-protosta-13(17),24-dien-3β-ol (**21**), product of PHS mutants F728A and F728S, in comparison to literature.[43] 66
Table S10. Crystallographic details of protostahopenol (**15**). 67
Table S11. Coding sequences of tested OSCs. 68
Table S12. Sequences of the primers used in this study 71

Experimental Procedures

General chemical methods

NMR spectra were recorded using Bruker Ultrashield 400, Ultrashield 500 or Ascend 600 MHz spectrometers operating at 400, 500 and 600 MHz for ^{1}H NMR and at 100, 126 and 151 MHz for ^{13}C NMR. $CDCl_3$ and C_6D_6 were used as solvents. Chemical shifts were referenced relative to the residual solvent signals ($CDCl_3$: δ_H = 7.26 ppm, δ_C = 77.16 ppm; C_6D_6: 7.16 ppm, δ_C = 128.06 ppm) and expressed in δ values (ppm), with coupling constants reported in Hz. Analysis was conducted with TopSpin (Version 4.0.6) or MestReNova (Version 14.2).
HRMS measurements were carried out on a Waters Acquity UPLC coupled to a Waters QTof Premier mass spectrometer.
Analytical and semipreparative LCMS analyses were performed on an Agilent Infinity II 1260 system consisting of a G7167A autosampler, G7116A column thermostat, G7111B quaternary pump, G7110B make-up pump, G7115A diode array detector, G1364F fraction collector, and G6125B single quadrupole mass spectrometer equipped with an ESI source (positive mode, 4000 V, 12 L/min drying gas, 350 °C gas temperature). 5 mM NH_4OAc was added to the mobile phase as indicated. The columns and gradients used are described below.
Automated flash chromatography was performed on a Biotage Isolera One with the stationary phases, solvents and gradients described below.
Reference compounds used in this manuscript were obtained from the following suppliers: Cycloartenol (**5**) by saponification and TMS-derivatisation of its cycloartenyl ferulate ester from γ-oryzanol[1] (Dragonspice Naturwaren, Reutlingen, Germany); lanosterol (**6**) from Avanti Polar Lipids (Alabaster, AL, USA); lupeol (**8**) from Biosynth (Staad, Switzerland). Hopenol B (**16**) and parkeol were generated *in situ* by transient expression of genes encoding *Aquilegia coerulea* hopenol B synthase or *Oryza sativa* parkeol synthase, respectively, as described below. Solvents and other chemicals were purchased from Sigma-Aldrich, Fisher Scientific and Carl Roth. All chemicals were directly used without further purification unless mentioned otherwise.

Mining of OSC sequences and sequence similarity network analysis

A metadata table of all plant samples within the 1KP data was obtained from the website at https://www.onekp.com/samples/list.php. Protein sequences ("xxxx-translated.protein.fa.gz") of all samples were obtained from GigaDB dataset 100910 (n = 1455).[2] OSC protein sequences were extracted based on BLASTP, PSI-BLAST, Pfam (profiles SQHop_N/C) and TIGRFAM (TIGR01787) searches using a custom R script. For BLASTP and PSI-BLAST, the 170 reference OSCs reported by Chen *et al.* were used.[3] Final results were filtered against E-values e−100 (BLAST/PSI-BLAST) and e−30 (Pfam/TIGRFAM) and a minimum length of 700 amino acids. The script and example data are provided on Figshare (DOI 10.6084/m9.figshare.26826439). The final list of 1,891 OSC sequences obtained by this approach plus 170 reference OSCs is also provided on Figshare (DOI 10.6084/m9.figshare.26826439).
Then, a sequence similarity network was generated from a FASTA file containing all 1,891 plus 170 reference OSC sequences on the EFI-EST website.[4–6] Option C (FASTA) with default parameters was chosen. The network was evaluated at different alignment score thresholds in the range 300-370. The optimal alignment score threshold used for final analysis was 360. The representative node network at 100% ID was used to eliminate duplicate sequences, resulting in 1,778 final nodes in the network. The resulting network file was visualised in Cytoscape 3.10.2 using the yFiles Organic layout 1.1.4.[7] The final Cytoscape session is also provided on Figshare (DOI 10.6084/m9.figshare.26826439).

Cloning and transient expression of candidate oxidosqualene cyclase genes in *Nicotiana benthamiana*

To test the function of candidate OSCs 1-6, transient expression in *Nicotiana benthamiana* was used. *Nicotiana benthamiana* LAB strain[8] was grown from seeds in a greenhouse with 11 to 16 hours illumination per day and at a temperature between 21 °C to 23 °C as described previously.[9]
Coding sequences of *OSC1-6* (Table S11) were obtained as synthetic genes from Genewiz (Leipzig, Germany). Coding sequences of *Aquilegia coerulea* hopenol B synthase[10] (accession number OM401331.1) for comparison with OSC6 / PHS and *Oryza sativa* parkeol synthase / OsOSC2[11] (accession number AK066327.1) for comparison with OSC6 / PHS mutant F728A were also obtained as synthetic genes. All genes were cloned into the plant expression vector pEAQ-HT[12] by In-Fusion HD cloning (Takara Bio) cloning or into pHREAC[13] by Golden Gate cloning, respectively, as described previously.[9] After cloning, the constructs were verified by Sanger sequencing and transformed into *A. tumefaciens* GV3101 by electroporation. Individual clones containing each target construct were cultured in 12 mL LB medium supplemented with antibiotics (50 µg/mL kanamycin, 50 µg/mL rifampicin and 50 µg/mL gentamicin) and incubated for two days at 28 °C with shaking (180 rpm). Cells were then centrifuged at 6,000 × *g* for 15 min, and the resulting pellet was resuspended in 5 mL MMA infiltration buffer (10 mM 2-(*N*-morpholino)ethanesulfonic acid (MES) buffer, pH 5.6, 10 mM $MgCl_2$, 100 µM acetosyringone) and then incubated for 2 - 4 hours at room temperature. Strains carrying candidate genes were mixed with *A. tumefaciens* strains carrying *Avena strigosa* truncated *HMGR* (KY284573) in pEAQ-HT.[14] *Agrobacterium* suspensions (final OD_{600} = 0.1 for each strain) were then infiltrated into 4–5-week-old *N. benthamiana* leaves as previously reported.[9] After a period of 7 days, three biological replicates (i.e., leaf disks from three different infiltrated plants) were collected for metabolite extraction and GC-MS analysis.

Metabolite extraction

Infiltrated leaves were harvested 7 days after infiltration. Ten leaf disks were harvested using cork borer no. 5 (10 mm) and lyophilised overnight. The dried leaf disks were ground with a Retsch MM 400 ball mill with two 5 mm diameter steel beads. Around 10-15 mg dry weight of infiltrated powdered leaf disks were used for metabolite extraction. A saponification solution (500 µL) of 10% (w/v) KOH in 90% ethanol was added to the ground leaves. The mixture was then incubated at 70 °C for one hour. After incubation, 250 µL of H_2O and 500 µL of *n*-hexane containing 10 µg/mL of the internal standard 5α-cholestane were added to the samples. The mixture was vortexed to ensure thorough mixing and then centrifuged at 342 × *g* and 40 °C for 10 minutes to facilitate phase separation. The organic layer was then transferred to a new glass vial and concentrated under reduced pressure.

GC-MS analysis

Prior to GC-MS measurement, samples were silylated using BSTFA:pyridine (1:1). GC-MS analysis was conducted using an HP 6890 GC system (HEWLETT PACKARD, Palo Alto, CA, USA) equipped with an OPTIMA 5MS column (MACHEREY-NAGEL, 30 m × 0.25 mm, capillary layer: 0.25 µm). A 5973N mass selective detector was employed to capture mass spectrometric data. The temperature program started at 100 °C and ramped up at a rate of 30 °C per minute until reaching 275 °C. Subsequently, the temperature was raised by 3 °C per minute until 300 °C was reached, where it was maintained for 15.83 minutes.
Injection volume was 1 µL and sample was injected with a 1:5 split ratio. The injector and detector temperatures were set to 300 °C and 250 °C, respectively. Helium was chosen as the carrier gas, with a flowrate of 1.5 mL per minute. Mass spectra were obtained with a scan range of *m*/*z* 43 to 850, with a solvent delay of 8 minutes after injection.

Purification of OSC products

For the isolation of OSC products, 5-week-old *N. benthamiana* plants (25-30 plants) were subjected to vacuum infiltration using a 9.2 L ROTILABO desiccator (Carl Roth, Karlsruhe, Germany) connected to an MZ 2 NT membrane pump (Vacuubrand, Wertheim, Germany) at 30 mbar for 1 minute. After 7 days post-infiltration, the leaves were harvested and freeze-dried for 2-3 days until a constant dry weight was reached. The resulting crude plant material was ground to a powder at room temperature using a blender and then extracted with ethyl acetate (50 mL / g dry weight) overnight. The extract was then filtered and concentrated in vacuo. The dried extracts were taken up with a minimal volume of ethanol until completely dissolved. Following this, an ion-exchange resin (Ambersep 900 OH, ca. 1 g per 3-4 mL ethanol) was added to the mixture while it was gently rotated to remove chlorophyll. The yellowish supernatant together with the resin was filtered over Celite 535; the resin was then additionally washed with ethanol, ethanol:petroleum ether (1:1), and petroleum ether. All filtrate and wash fractions were combined and concentrated *in vacuo*. The final extracts were purified by successive rounds of flash chromatography (Biotage Isolera) followed by a purification with a semi-preparative LC-MS (Agilent Infinity II instrument as described above). The conditions and gradients for compound isolation were:

**(3*S*,13*S*)-Malabarica-17,21-diene-3β,14-diol (12)**	**Plant dry weight:**			7.6 g	
	**Extraction solvent:**		Ethyl acetate	**Crude extract:**	561 mg
	**Ion exchanger:**		Ambersep 900 OH	**Filtered extract:**	270 mg
	**Instrument**	**Column**	**Solvents**	**Gradient**	**Yield**
	Biotage	SNAP Ultra 10 g	A: Petroleum ether<br>B: Ethyl acetate	0-40% B (10 CV)<br>40% B (3 CV)<br>40-100% B (2 CV)	181 mg
	Biotage	KP Sil 10 g	A: Petroleum ether<br>B: Ethyl acetate	0-3% B (10 CV)<br>3-50% B (3 CV)<br>50% B (2 CV)	70 mg
	Biotage	SNAP Ultra 10 g	A: Petroleum ether<br>B: Ethyl acetate	0-30% B (3 CV)<br>30% B (10 CV)<br>30-100% B (2 CV)<br>100% B (2 CV)	42 mg
	Biotage	Sfaer C18 D 12 g	A: H_2O/NH_4OAc<br>B: Methanol/NH_4OAc	90-100% B (10 CV)<br>100% B (15 CV)	4 mg

**19-*epi*-Lupeol (14)**	**Plant dry weight:**			4.3 g	
	**Extraction solvent:**		Ethyl acetate	**Crude extract:**	319 mg
	**Ion exchanger:**		Ambersep 900 OH	**Filtered extract:**	200 mg
	**Instrument**	**Column**	**Solvents**	**Gradient**	**Yield**
	Biotage	SNAP Ultra 10 g	A: Petroleum ether<br>B: Ethyl acetate	0-40% B (10 CV)<br>40% B (3 CV)<br>40-100% B (2 CV)<br>100% B (2 CV)	33 mg

	Biotage	SNAP Ultra 10 g	A: Petroleum ether<br>B: Ethyl acetate	0-12% B (5 CV)<br>12% B (10 CV)<br>12-40% B (3 CV)<br>40% B (3 CV)<br>40-100% B (3 CV)<br>100% B (3 CV)	22 mg
	LC-MS	Luna 5 µm C8 100 Å<br>250 x 10 mm	A: H_2O/NH_4OAc<br>B: Methanol/NH_4OAc<br>Flowrate: 5 mL/min	90-100% B (6 min)<br>100% B (5 min)<br>100-90% B (0.1 min)<br>90% B (2 min)	7 mg

**Dammarenediol II (18)**	**Plant dry weight:**			15.7 g	
	**Extraction solvent:**		Ethyl acetate	**Crude extract:**	1.521 g
	**Ion exchanger:**		Ambersep 900 OH	**Filtered extract:**	813 mg
	**Instrument**	**Column**	**Solvents**	**Gradient**	**Yield**
	Biotage	Sfaer Silica HC D<br>10 g	A: Petroleum ether<br>B: Ethyl acetate	0-40% B (10 CV)<br>40% B (5 CV)<br>40-100% B (2 CV)<br>100% B (3 CV)	81 mg
	Biotage	Sfaer Silica HC D<br>5 g	A: Petroleum ether<br>B: Ethyl acetate	0-20% B (3 CV)<br>20% B (10 CV)<br>20-40% B (2 CV)<br>40% B (3 CV)<br>40-100% B (2 CV)<br>100% B (3 CV)	35 mg
	LC-MS	Luna 5 µm C8 100 Å<br>250 x 10 mm	A: H_2O/NH_4OAc<br>B: Methanol/NH_4OAc<br>Flowrate: 5 mL/min	85-100% B (8 min)<br>100% B (3 min)<br>100-85% B (0.1 min)<br>85% B (2 min)	16 mg

**Camelliol C (19)**	**Plant dry weight:**			11 g	
	**Extraction solvent:**		Ethyl acetate	**Crude extract:**	811 mg
	**Instrument**	**Column**	**Solvents**	**Gradient**	**Yield**
	Biotage	SNAP KP Sil 25g	A: Petroleum ether<br>B: Ethyl acetate	0-40% B (10 CV)<br>40% B (5 CV)<br>40-100% B (2 CV)<br>100% B (5 CV)	121 mg
	Biotage	Sfaer Silica HC D<br>5 g	A: Petroleum ether<br>B: Ethyl acetate	0-12% B (3 CV)<br>12% B (10 CV)<br>12-100% B (2 CV)<br>100% B (3 CV)	74 mg
	Open column	Silica (7 g)	DCM	DCM (4 CV)	36 mg
	LC-MS	Luna 5 µm C8 100 Å 250 x 10 mm	A: H_2O/NH_4OAc<br>B: Methanol/NH_4OAc<br>Flowrate: 4 mL/min	98-99.7% B (8 min)<br>99.7-98% B (0.1 min)<br>98% B (2 min)	5.9 mg

**Protostahopenol (15)**	**Plant dry weight:**			27 g	
	**Extraction solvent:**		Ethyl acetate	**Crude extract:**	3.39 g
	**Instrument**	**Column**	**Solvents**	**Gradient**	**Yield**
	Biotage	SNAP KP Sil 100 g	A: Petroleum ether<br>B: Ethyl acetate	0-40% B (13 CV)	549 mg
	Biotage	SNAP KP Sil 25 g	A: Petroleum ether<br>B: DCM<br>C: Methanol	25% B; 75% A (1 CV)<br>50% B; 50% A (1 CV)<br>75% B; 75% A (5 CV)<br>100% B (3 CV)<br>80% B; 20% C (3 CV)	155 mg
	Open column	Silica (15 g)	A: Petroleum ether<br>B: Ethyl acetate	7.5% B (14 CV)	92 mg

**(20*R*)-Protosta-13(17),24-dien-3β-ol (21)**	**Plant dry weight:**			30 g	
	**Extraction solvent:**		Ethyl acetate	**Crude extract:**	1.92 g
	**Instrument**	**Column**	**Solvents**	**Gradient**	**Yield**
	Biotage	SNAP KP Sil 50g	A: Petroleum ether<br>B: Ethyl acetate	0-40% B (9 CV)<br>40% B (4 CV)<br>40-100% B (1 CV)<br>100% B (3 CV)	108 mg
	Biotage	Sfaer Silica HC D 5g	A: Petroleum ether<br>B: Ethyl acetate	0-12% B (5 CV)<br>12% B (10 CV)<br>12-100% B (3 CV)<br>100% B (5 CV)	93 mg
	Biotage	Sfaer Silica HC D 5g	A: Petroleum ether<br>B: Ethyl acetate	0-10% B (15 CV)<br>10% B (5 CV)<br>10-100% B (2 CV)<br>100% B (3 CV)	53 mg
	Open column	Silica (5 g)	A: Dichloromethane<br>B: Ethyl acetate	100% A (1 CV)<br>98% A (4 CV)	42 mg
	LC-MS	Luna 5 µm C8 100 Å<br>250 x 10 mm	B: Methanol/NH_4OAc<br>Flowrate: 4 mL/min	100% B (12 Min)	19.7 mg
	Open column	Silica (2 g)	A: Dichloromethane<br>B: Ethyl acetate<br>C: Toluene	100% C (2 CV)<br>80% C; 20% A (5 CV)<br>100% A (1 CV)<br>100% B (4 CV)	15 mg<br>(only 5 mg used for the next step)
	LC-MS	Luna 5 µm C8 100 Å<br>250 x 10 mm	A: H_2O/NH_4OAc<br>B: Methanol/NH_4OAc<br>Flowrate: 4 mL/min	86% B (62 min)<br>86-100% B (0.1 min)<br>100% B (5 min)<br>100-86% B (0.1 min)<br>86% B (5 min)	2.3 mg

Analytical data

(3*S*,13*S*)-Malabarica-17,21-diene-3β,14-diol (12):
HR-ESI-MS: 427.3925 $[M + H - H_2O]^+$ (calc. for $C_{30}H_{51}O^+$ 427.3934).
EI mass spectra see Figure S12 (four peaks were observed for pure **12**).
^1H and ^{13}C NMR data see Table S3. NMR spectra see Figure S28-Figure S29.

19-*epi*-Lupeol (14)
HR-ESI-MS: 409.3850 $[M + H - H_2O]^+$ (calc. for $C_{30}H_{49}^+$ 409.3829).
EI mass spectrum see Figure 3.
^1H and ^{13}C NMR data see Table S4 and Table S5. NMR spectra see Figure S30-Figure S35.

Protostahopenol (15)
HR-ESI-MS: 427.3899 $[M + H]^+$ (calc. for $C_{30}H_{51}O^+$, 427.3934).
EI mass spectrum see Figure 3.
^1H and ^{13}C NMR data see Table S6. NMR spectra see Figure S36-Figure S41.
Crystallographic details see Table S10.

Dammarenediol II (18)
HR-ESI-MS: 467.3848 $[M + Na]^+$ (calc. for $C_{30}H_{52}O_2Na^+$, 467.3860).
EI mass spectra see Figure S23 (two peaks were observed from pure **14**).
^1H and ^{13}C NMR data see Table S7. NMR spectra see Figure S42-Figure S43.

Camelliol C (19)
HR-ESI-MS: 427.3940 $[M + H]^+$ (calc. for $C_{30}H_{51}O^+$, 427.3934).
EI mass spectrum see Figure S23.
^1H and ^{13}C NMR data see Table S8. NMR spectra see Figure S44-Figure S45.

(20*R*)-Protosta-13(17),24-dien-3β-ol (21)
HR-ESI-MS: 409.3824 $[M + H - H_2O]^+$ (calc. for $C_{30}H_{49}^+$, 409.3829).
EI mass spectrum see Figure S23.
^1H and ^{13}C NMR data see Table S9. NMR spectra see Figure S46-Figure S47.

X-ray diffraction measurements

Single crystals of protostahopenol (**15**) were obtained by slow evaporation from a dichloromethane-methanol (1:2) solution at 4 °C. Single crystal x-ray diffraction measurement was carried out on an XtaLAB AFC12 (RINC): Kappa single diffractometer operating at T = 100.00(10) K using Cu Kα radiation (λ = 1.54184 Å). The diffraction pattern was indexed and the total number of runs and images was based on the strategy calculation from the program CrysAlisPro (Rigaku). Data reduction, scaling and absorption corrections were performed using CrysAlisPro (Rigaku, V1.171.43.95a, 2023). The structure was solved with the ShelXT[15] structure solution program using the Intrinsic Phasing solution method and by using Olex2[16] as the graphical interface. The model was refined with version 2019/3 of ShelXL 2019/3 using Least Squares minimisation.[17] All non-hydrogen atoms were refined anisotropically. Hydrogen atom positions were calculated geometrically and refined using the riding model. Hydrogen atom positions were calculated geometrically and refined using the riding model. Crystallographic data have been deposited in the CCDC database. CCDC 2379089 contains CIF file for 2(**15**) • MeOH.

Bioinformatics analyses, docking, and selection of targets for site-directed mutagenesis

A multiple sequence alignment of OSCs1-6 with 170 reference OSCs reported in ref. [3], three hopanoid synthases from ref. [10] and human lanosterol synthase (1W6K)[18] as an outgroup was generated using the MUSCLE algorithm in Geneious 9.1.8 (https://www.geneious.com). From the resulting alignment, a phylogenetic tree was constructed using PhyML 3.1 with the JTT model and 100 bootstrap replicates.[19] The final phylogenetic tree is shown in Figure S9. The underlying multiple sequence alignment is visualized in Figure S10 as a heatmap.

Structural models of MDDS, 19ELS, and PHS were generated with AlphaFold 2.2.4 with default settings.[20] The position of the active site of OSCs was identified by comparison of the structural model to the crystal structure of human lanosterol synthase (1W6K) in complex with the reaction product lanosterol.[18] The protein models were prepared for docking with ADFRsuite 1.0. Products of MDDS, 19ELS, and PHS were prepared for docking with AutoDockTools 1.5.6 and docked into the active site using AutoDock Vina 1.2.3.[21,22] For (3*S*,13*S*)-malabarica-17,21-diene-3β,14-diol (**12**), both possible C14 epimers were used. All structures and docking results were analysed in UCSF ChimeraX 1.8, developed by the Resource for Biocomputing, Visualization, and Informatics at the University of California, San Francisco, with support from National Institutes of Health R01-GM129325 and the Office of Cyber Infrastructure and Computational Biology, National Institute of Allergy and Infectious Diseases.[23] Poses that showed a similar orientation as lanosterol in lanosterol synthase, particularly regarding the interaction with the catalytic acidic residue Asp455, were considered as realistic and used for further analysis. The resulting docking poses are shown in Figure S19.

To identify residues and mutations for site-directed mutagenesis, two strategies were combined: First, all residues within a zone of 5 Å around the docked products were identified as potentially relevant for catalytic activity. Then, a multiple sequence alignment with OSCs with a similar product profile (e.g., OSCs producing lupeol (**8**) compared to 19ELS) was generated using the ClustalOmega algorithm in R 4.2.1 with the package msa 1.28.0 (Figure S20, Figure S21, Figure S22). Amongst the active site residues, non-conserved positions were identified from the multiple sequence alignment and prioritised based on the position of the residues in the structural model (e.g., for 19ELS, residues close to C19 were prioritised).

Read coverage of *OSC* transcripts was analyzed by mapping raw reads with bowtie2 (v 2.3.5.1) to the assembled transcriptome sequences provided by the One Thousand Plant Transcriptomes initiative.[2,24] The following files were used: For *OSC2*, read pair ERR2040457_1/_2.fast.gz and assembly WYIG-SOAPdenovo-Trans-assembly.fa; for OSC3, read pair ERR2040311_1/_2.fastq.gz and assembly HDWF-SOAPdenovo-Trans-assembly.fa. The resulting alignments were sorted and indexed with samtools 1.21 [25] and visualized with IGV 2.8.13 [26].

Common protein motifs of OSCs as reported before[3,27] were extracted from the multiple sequence alignment of OSC amino acid sequences described above and visualized with Logomaker[28] in Python.

Site-directed mutagenesis

Site-directed mutagenesis was performed using Golden Gate cloning. Primers were designed to introduce suitable recognition and restriction sites, facilitated by the Golden Mutagenesis online tool (Table S12).[29] Mutated sequences were generated by PCR, then cloned into the plant expression vector pHREAC[13] using Golden Gate cloning. The resulting constructs were transformed into *A. tumefaciens* GV3101 and used for transient expression in *N. benthamiana* as described above. Metabolite extraction and GC-MS analysis were also conducted as described above. For data analysis, peak areas of new products from the mutants were normalised by dividing by the peak area of the internal standard 5α-cholestane and the dry weight of the sample. The relative normalised peak area was finally obtained by dividing by the normalised peak area of the main product of the wild type OSC. For (3*S*,13*S*)-malabarica-17,21-diene-3β,14-diol (**12**) and dammarenediol II (**18**), which show multiple peaks under GC-MS conditions (Figure S12), all product peak areas were summed.

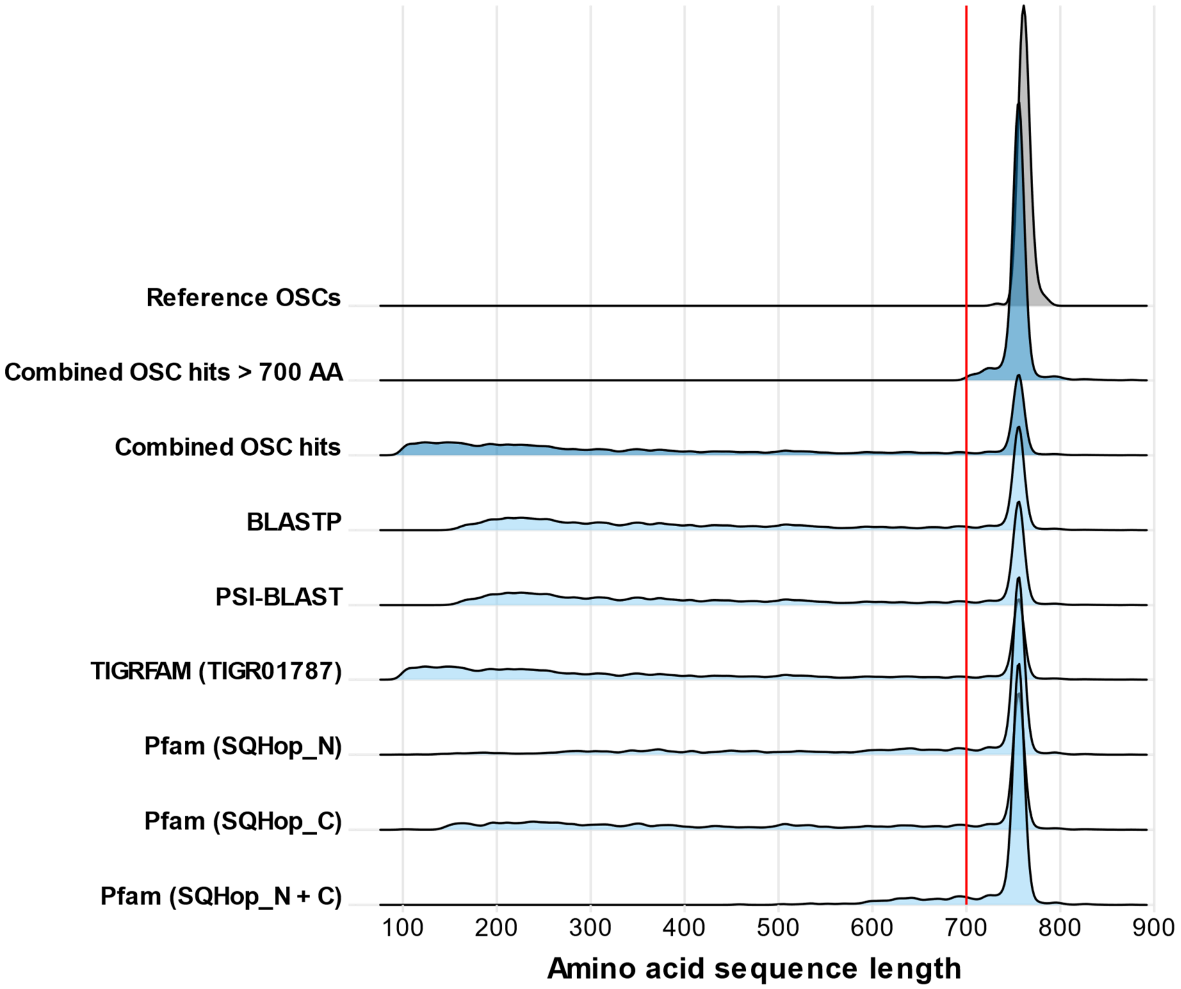

Figure S1. Amino acid sequence length distributions of potential OSCs found by different search strategies in comparison to the 170 reference OSCs collected by Chen *et al.*[3]

The red vertical line indicates the chosen length cut-off of 700 amino acids (AA) for the final dataset of 1,891 full-length OSCs. Data is shown as a ridgeline plot (bandwidth = 5).

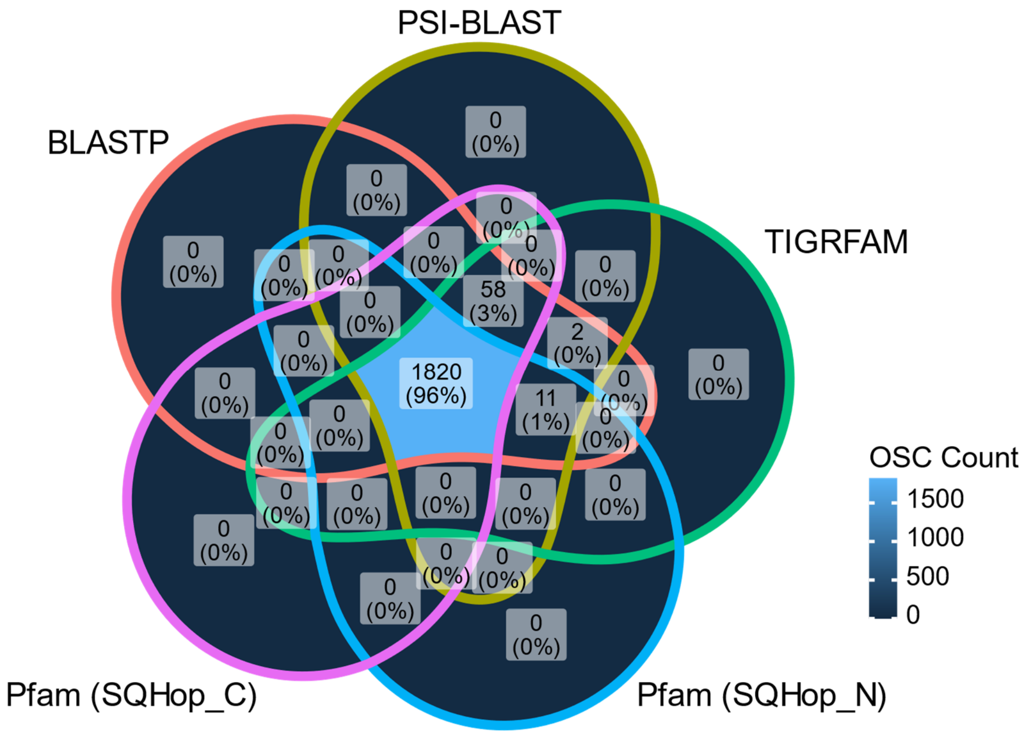

Figure S2. Venn diagram of OSC count identified by different search strategies (BLASTP, PSI-BLAST, Pfam (SQHop_N and SQHop_C profiles) and TIGRFAM (TIGR01787)).

In total, 1,891 OSCs were found.

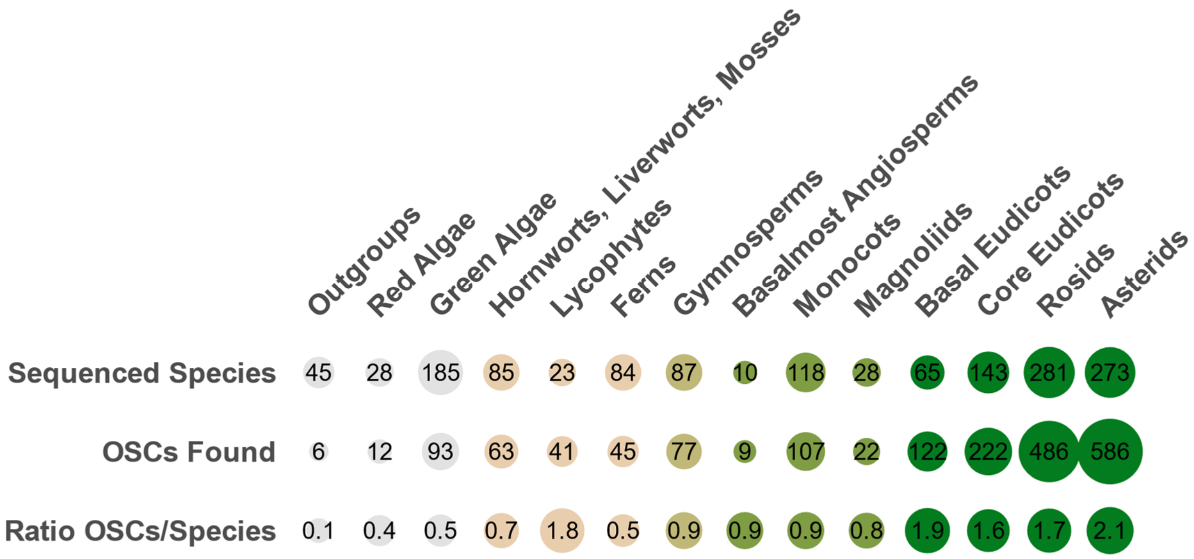

Figure S3. Phylogenetic distribution of oxidosqualene cyclases (OSCs) identified from the One Thousand Plant Transcriptomes dataset in comparison to the number of sequenced species per clade.

Clade assignments were used as published,[2] but were additionally pooled for simplification as described in Table S1.

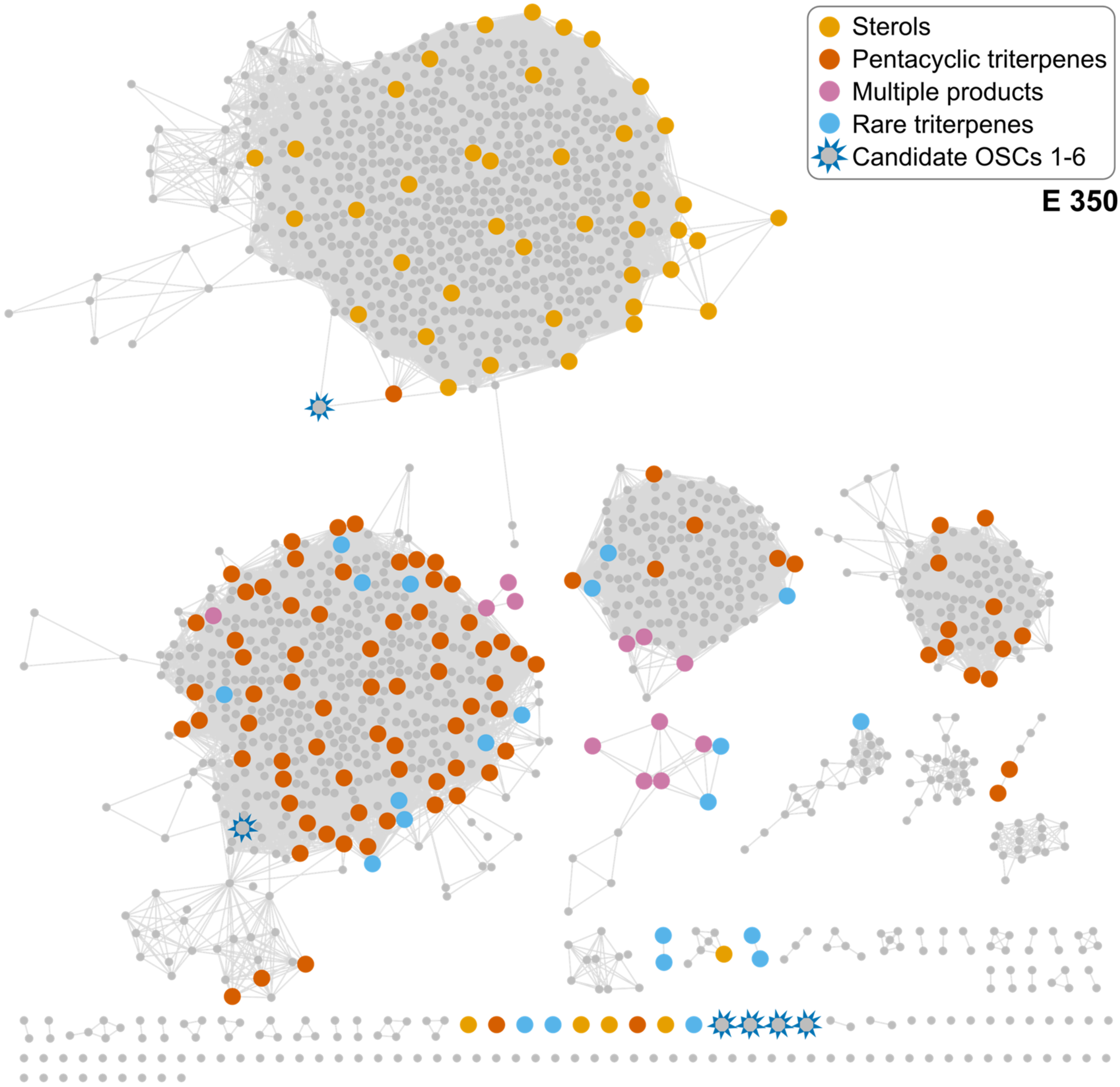

Figure S4. Sequence similarity network of 1,891 OSCs and 170 reference OSCs at a sequence alignment threshold of E 350.

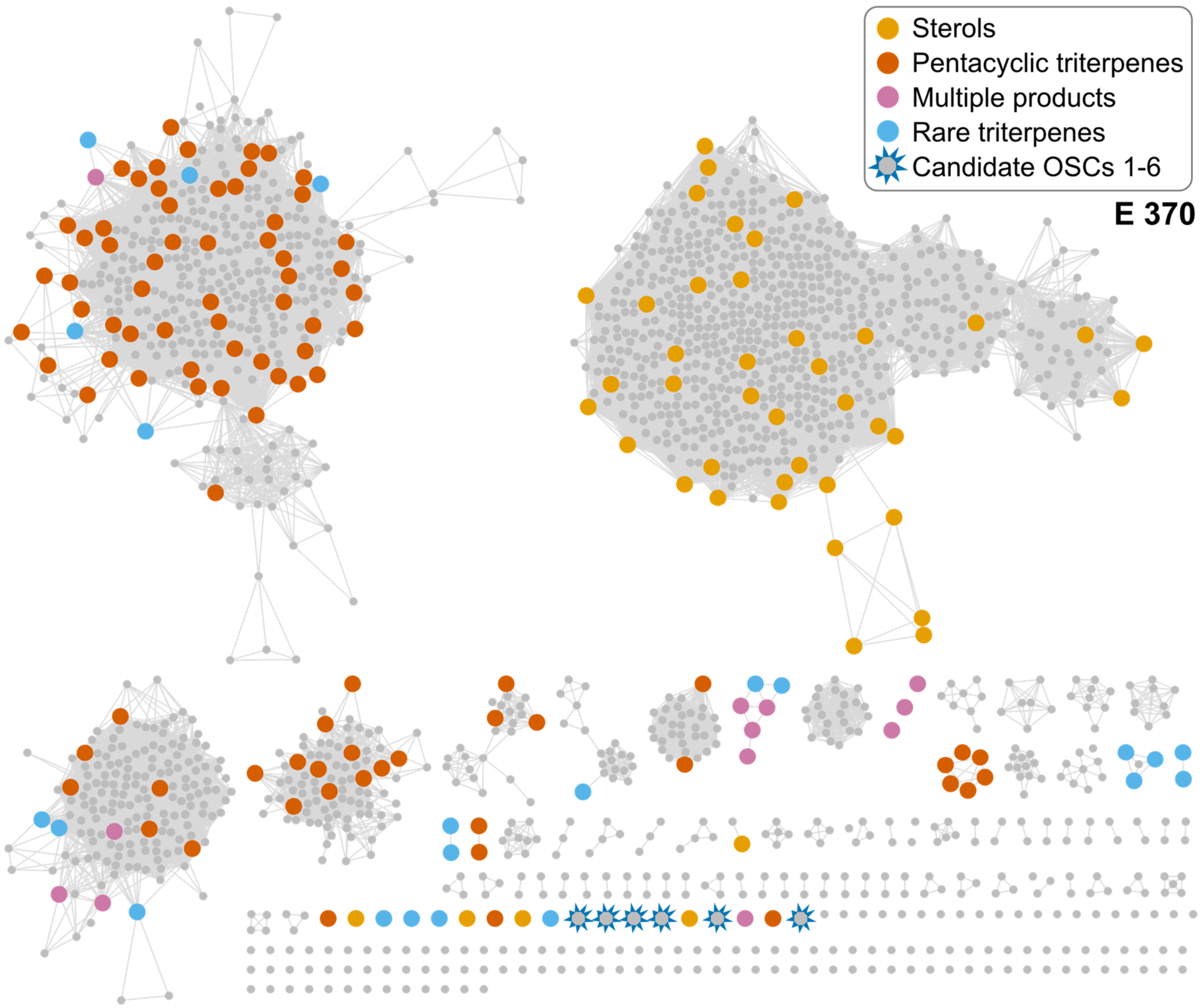

Figure S5. Sequence similarity network of 1,891 OSCs and 170 reference OSCs at a sequence alignment threshold of E 370.

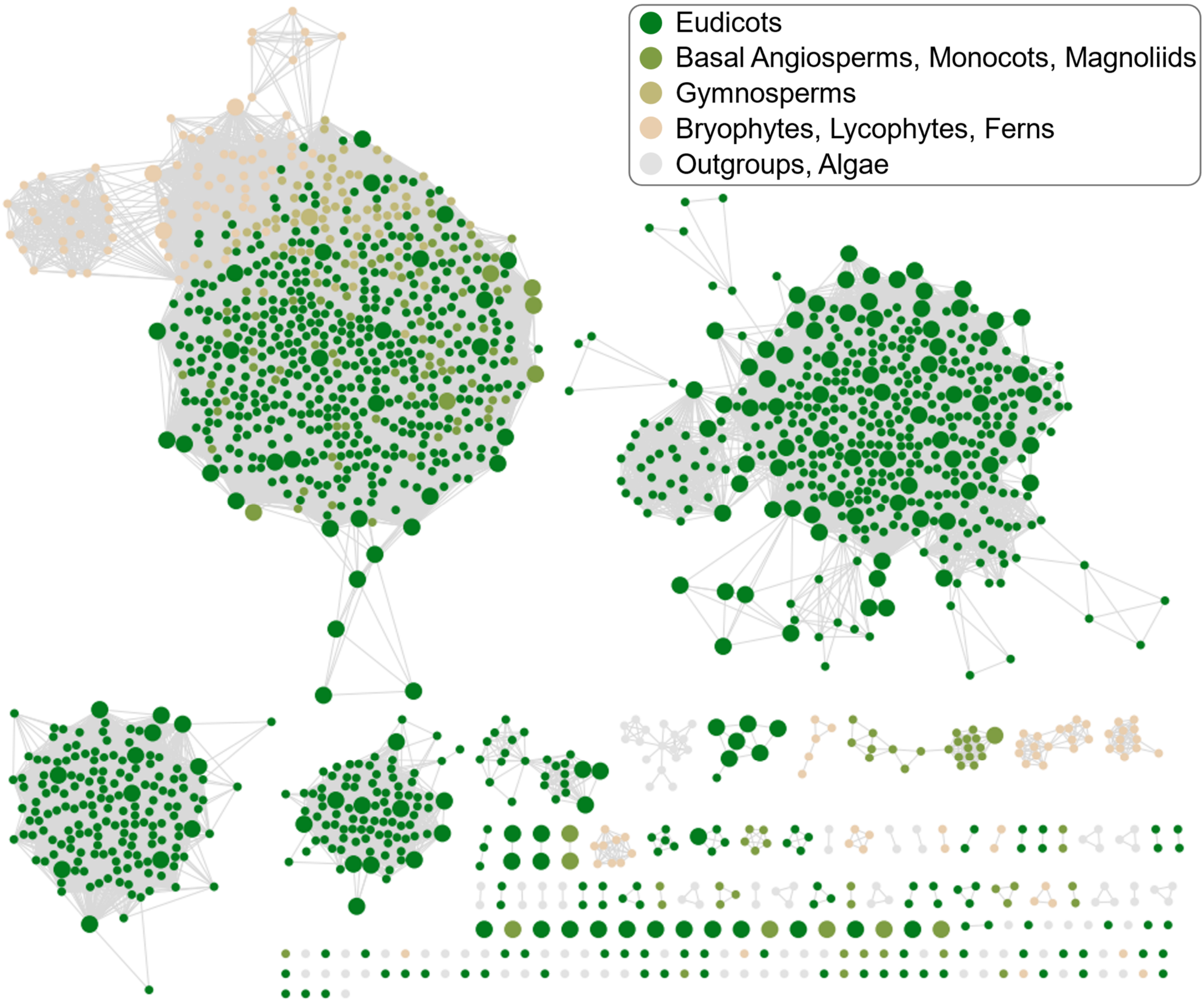

Figure S6. Sequence similarity network of 1,891 OSCs and 170 reference OSCs highlighting their phylogenetic position.

See also Table S1 and Figure S3 for details regarding the clades and colour code.

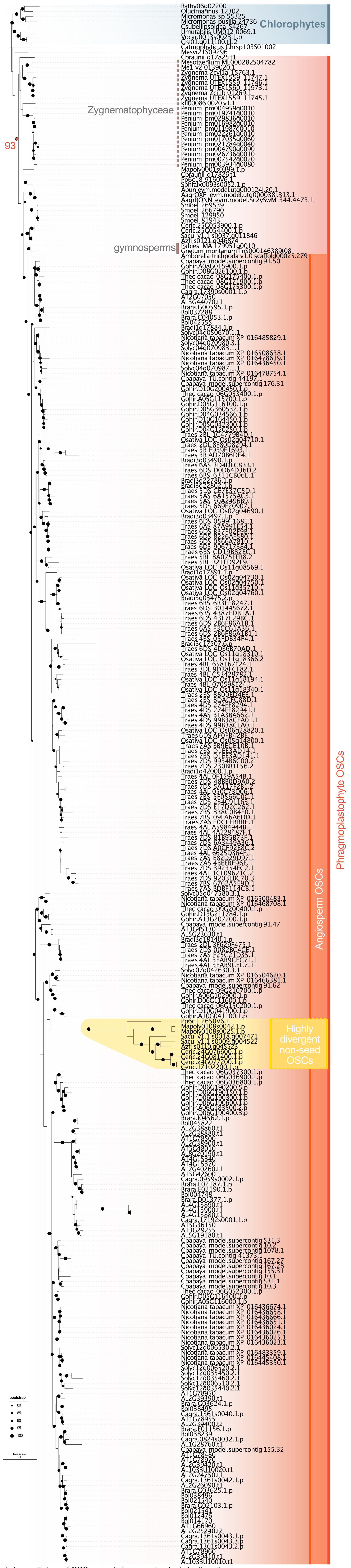

Figure S7. Maximum likelihood phylogenetic tree of OSCs sampled across streptophyte diversity.

298 OSC homologs in streptophytes and 8 in chlorophytes were sampled using BLAST, aligned, and a maximum likelihood phylogeny under the best-fit model JTT+G4 (according to Bayesian Information Criterion) was computed with 5000 ultrafast bootstrap pseudoreplicates. Note the massive diversification of OSCs within angiosperms; the clade of highly divergent OSCs found in a few non-seed plants likely falls into the angiosperm clade because of its long branch.

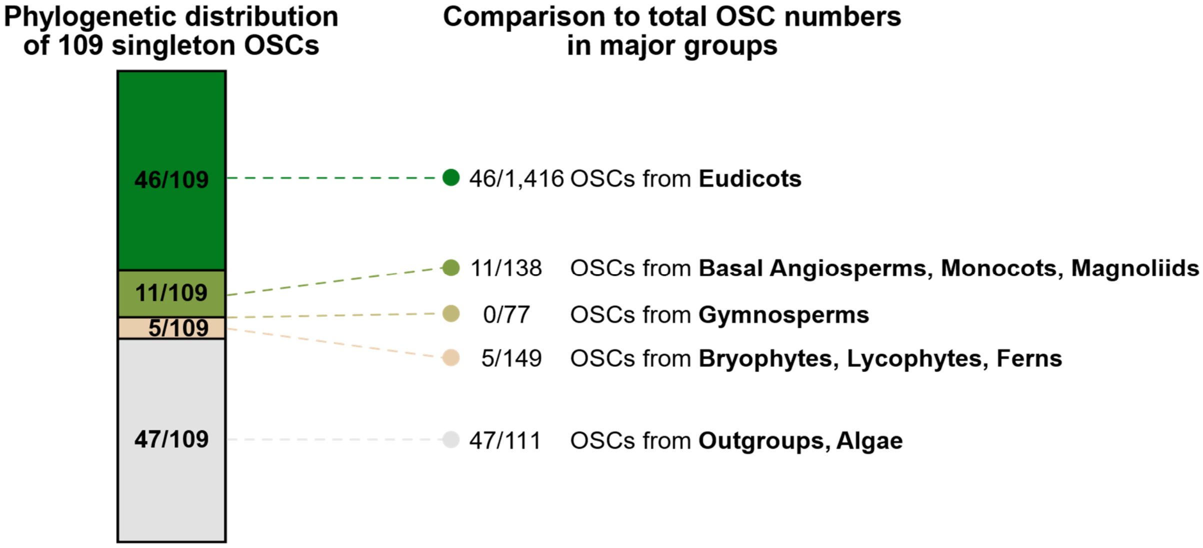

Figure S8. Phylogenetic distribution of 109 singleton OSCs identified from One Thousand Plant Transcriptomes dataset in relation to the total numbers of OSCs from these groups.

The overrepresentation of singleton OSCs from algae during SSN analysis is likely caused by the large phylogenetic distance. For details regarding groups see Table S1.

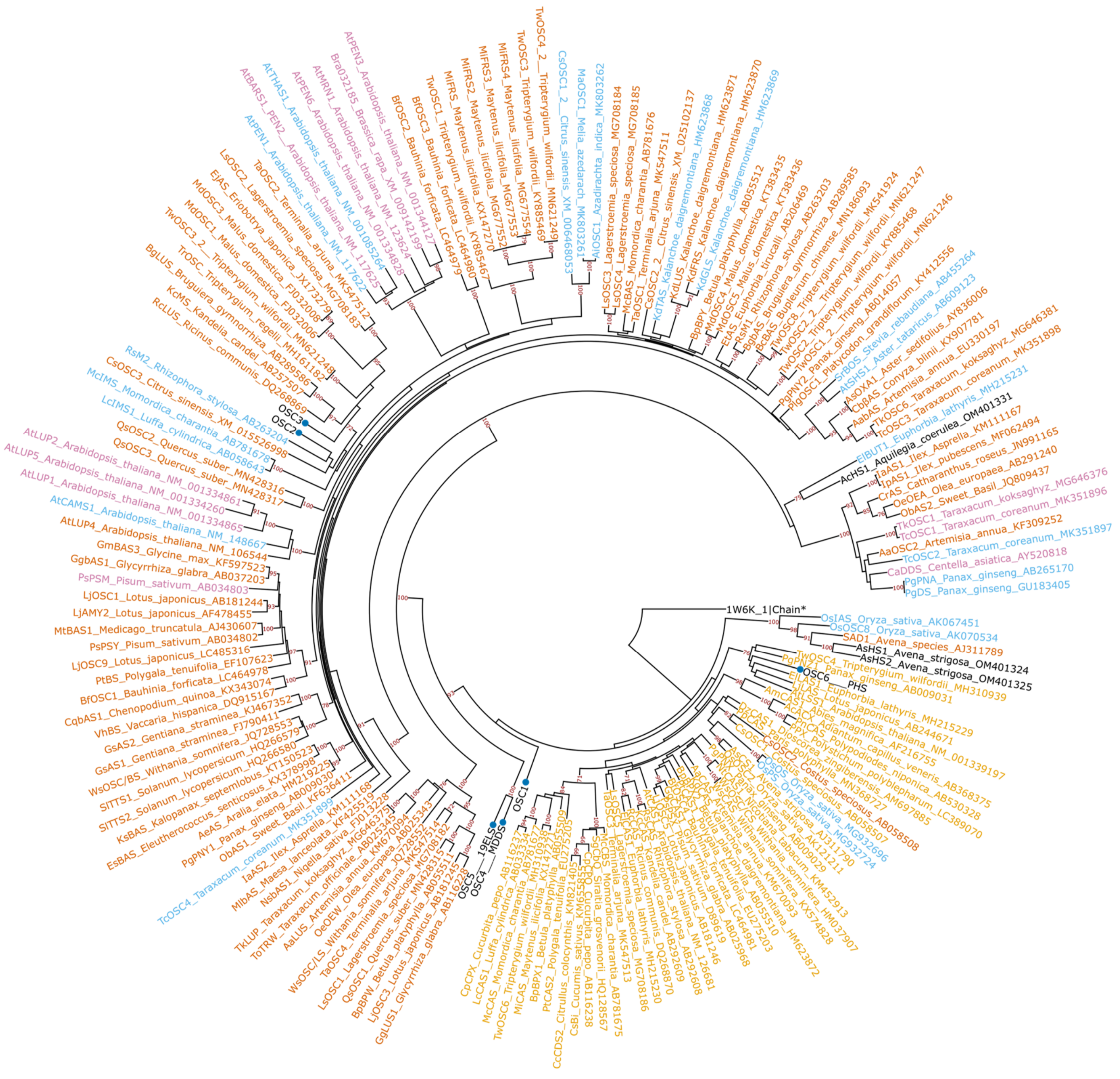

Figure S9. Maximum likelihood phylogenetic tree of OSC1-6, 170 reference OSCs from ref. [3], and three hopanoid synthases from ref. [10].

OSCs1-6 are indicated by a blue dot. Colors of reference OSCs correspond to the product color code from Figure 2. Human lanosterol synthase (1W6K)[18] was used as an outgroup. Numbers in red represent bootstrap values from 100 replicates.

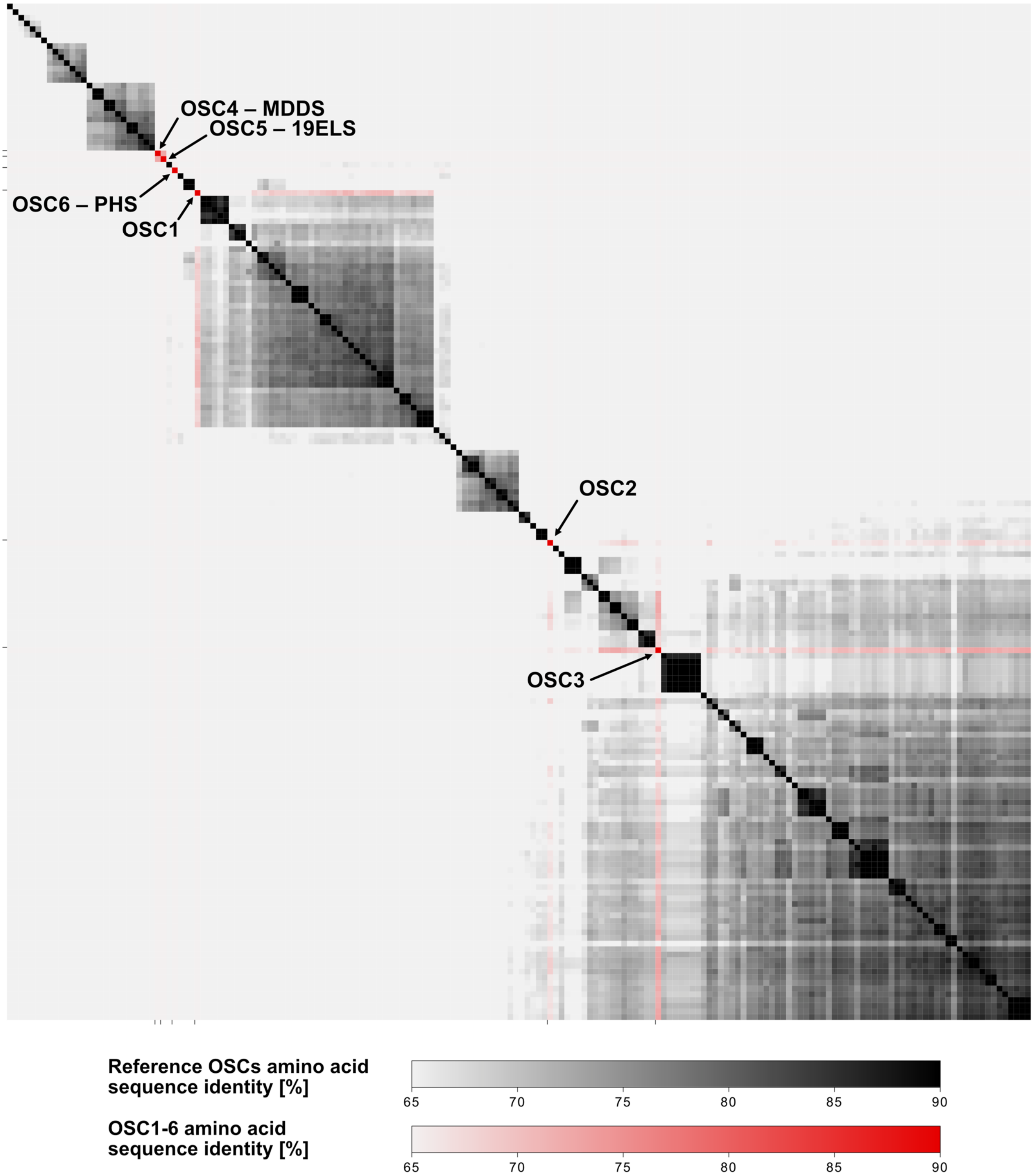

Figure S10. Heatmap representation of the multiple sequence alignment of reference OSCs[3] and OSCs 1-6 underlying the phylogenetic tree in Figure S9.

In contrast to most reference OSCs, OSCs1-6 share limited sequence identities <= 73% with previously characterized OSCs, emphasizing their distance in sequence space.

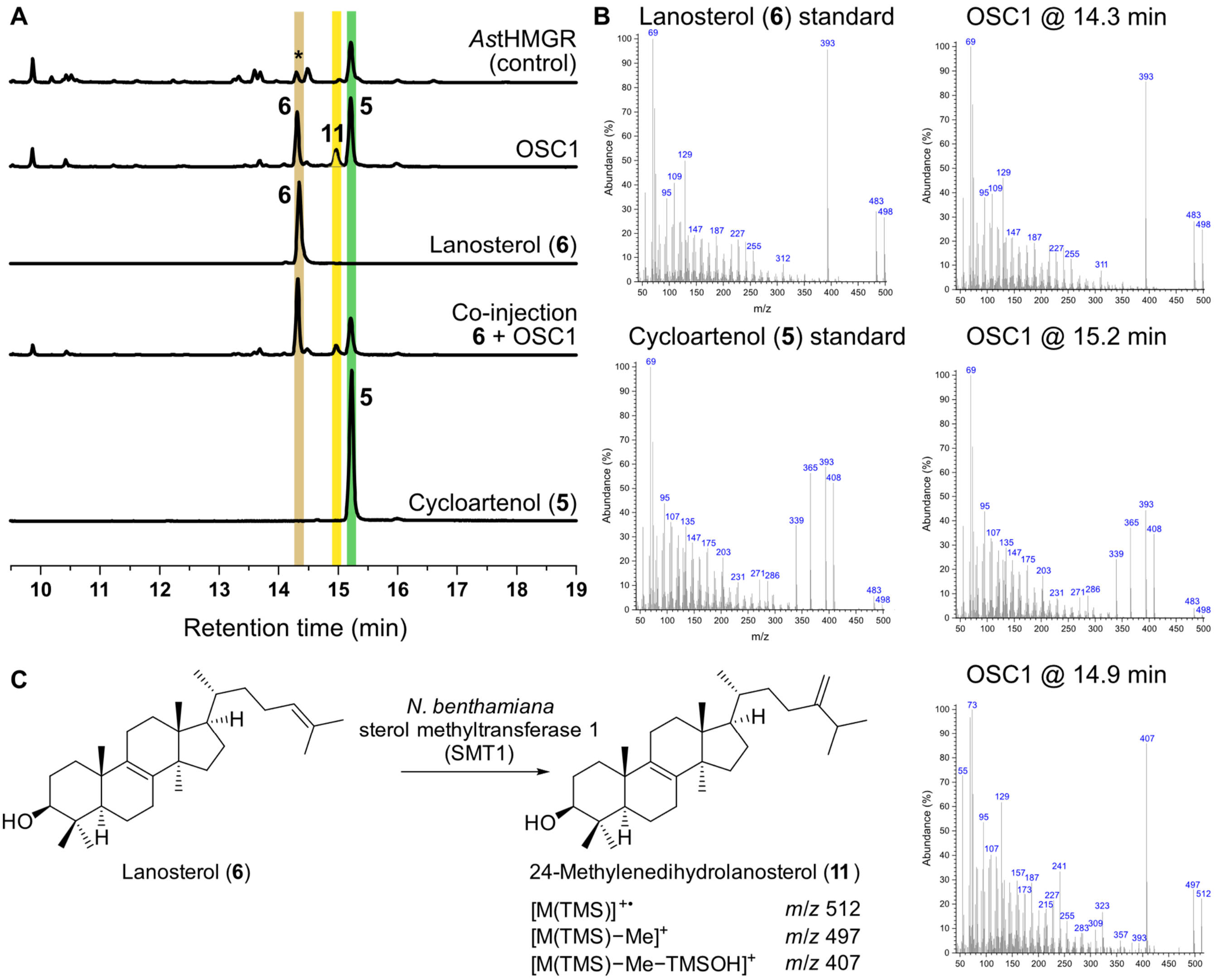

Figure S11. Production of lanosterol (**6**) and cycloartenol (**5**) by OSC1 and of the *N. benthamiana* shunt product 24-methylenedihydrolanosterol (**11**).

A) Chromatograms of OSC1 peaks in comparison to reference compounds of lanosterol (**6**) and cycloartenol (**5**). The peak in the *Ast*HMGR control at 14.3 min (indicated by *) is not lanosterol (**6**) as judged by the mass spectrum.

B) Mass spectra of OSC1 products and reference compounds **5** and **6**. Compound **11** was assigned as 24-methylenedihydrolanosterol based on comparison of the mass spectrum to literature.[30]

C) Proposed formation of 24-methylenedihydrolanosterol (**11**) *via* promiscuous methylation of lanosterol (**6**) by *N. benthamiana* sterol methyltransferase 1 (SMT1).[31]

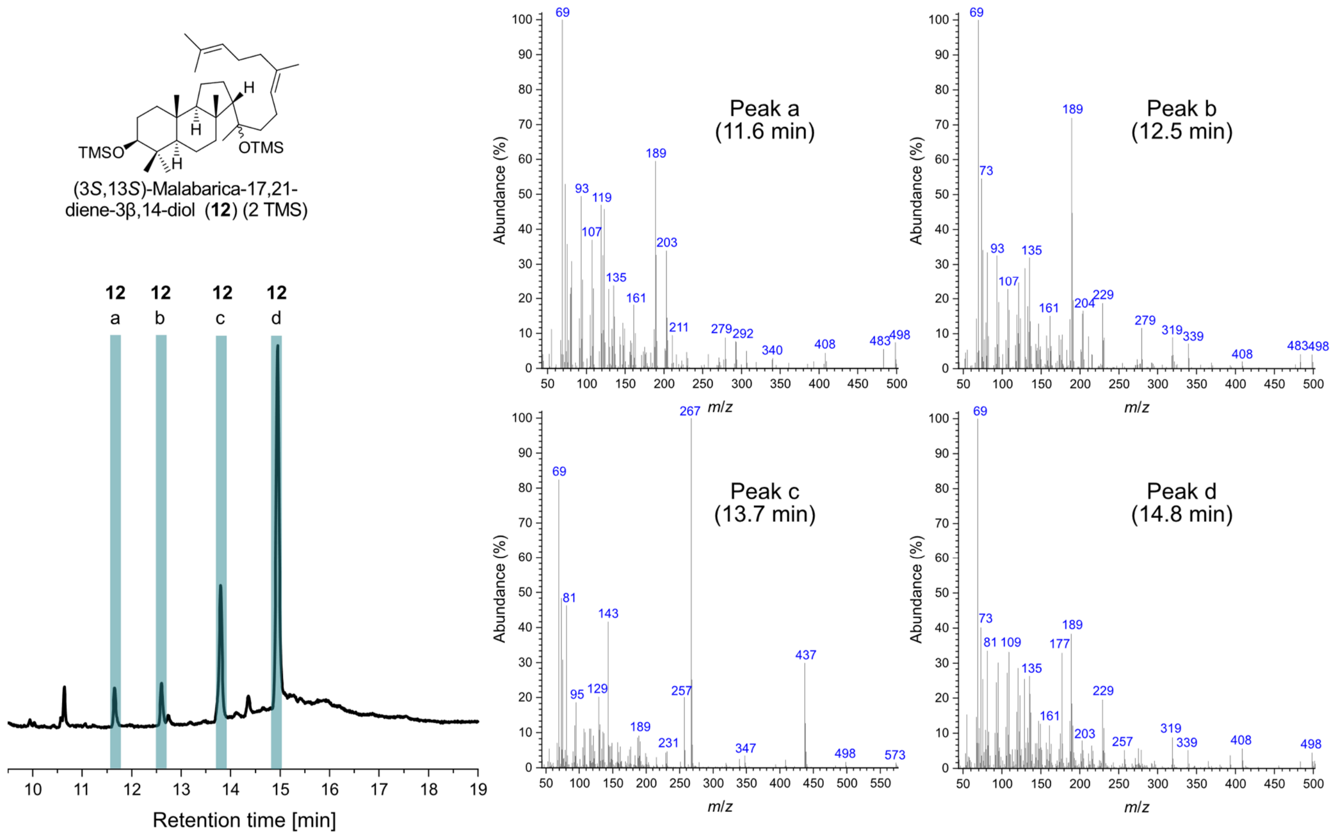

Figure S12. Pure (3*S*,13*S*)-malabarica-17,21-diene-3β,14-diol (**12**) shows four peaks a, b, c, and d upon saponification, derivatisation, and GC-MS analysis.

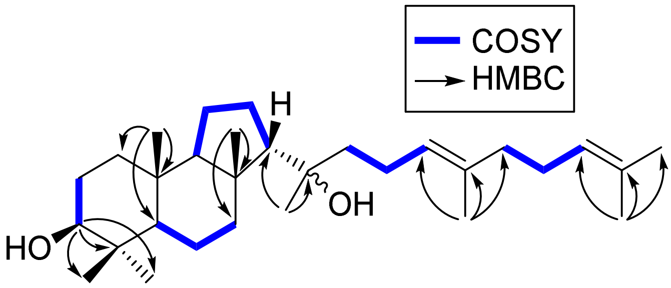

Figure S13. Key NMR correlations of (3*S*,13*S*)-malabarica-17,21-diene-3β,14-diol (**12**).

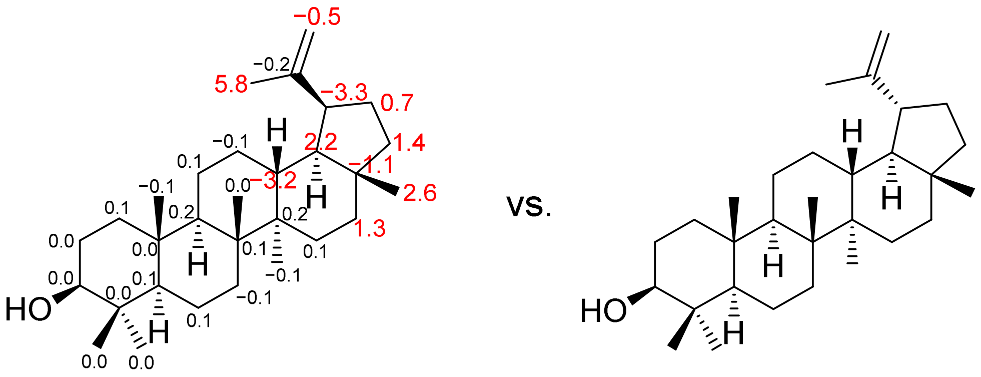

Figure S14. Position-specific ^{13}C NMR shift differences between 19-*epi*-lupeol (**14**) and lupeol (**8**) (C_6D_6).

Differences ≥ 0.5 ppm are highlighted in red.

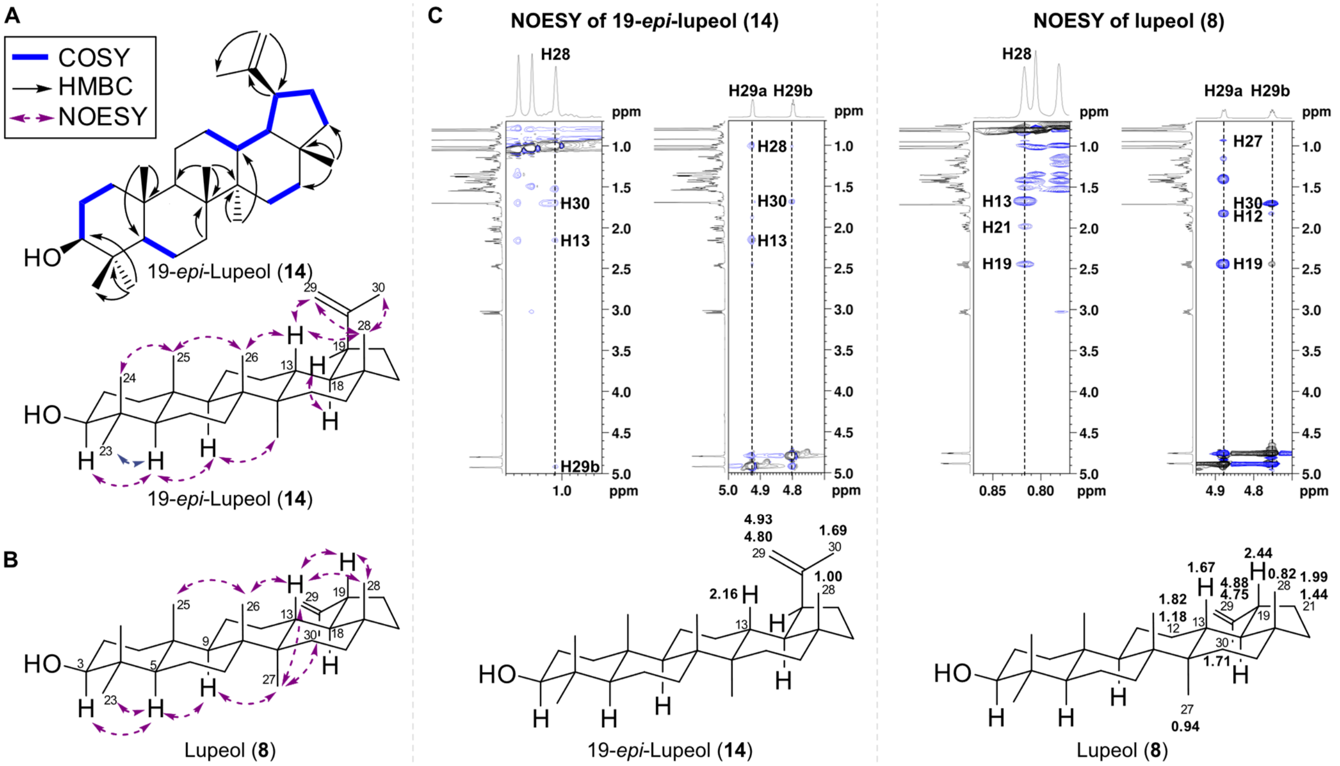

Figure S15. Key NMR correlations of 19-*epi*-lupeol (**14**) in comparison with lupeol (**8**).

A) Key COSY, HMBC, and NOESY correlations of 19-*epi*-lupeol (**14**). B) Key NOESY correlations of lupeol (**8**) for comparison. C) Sections from original NOESY spectra with chemical shifts of relevant protons (C_6D_6, 500 and 600 MHz, respectively, 298 K).

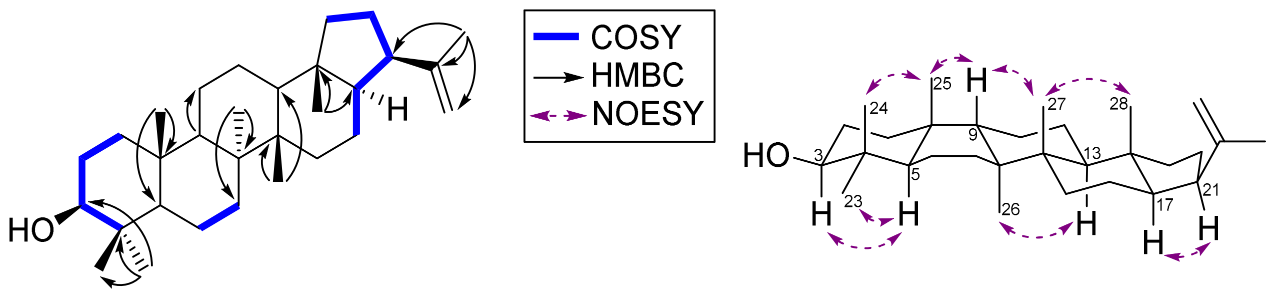

Figure S16. Key NMR correlations of protostahopenol (**15**).

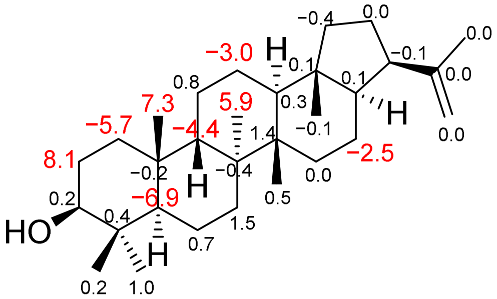

Protostahopenol (**15**)

vs.

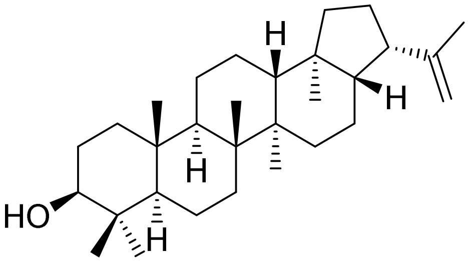

Hopenol B (**16**)

Figure S17. Position-specific ^{13}C NMR shift differences between protostahopenol (**15**) and hopenol B (**16**).

Differences ≥ 2.0 ppm are highlighted in red.

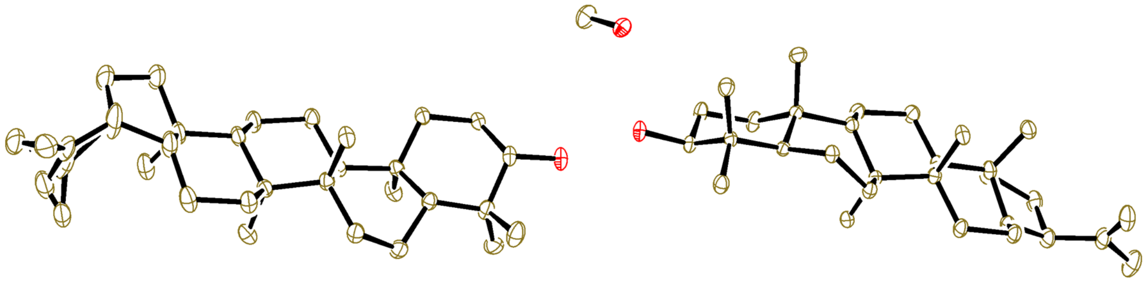

Figure S18. ORTEP of protostahopenol (**15**) with ellipsoids drawn at the 50% probability level and hydrogens omitted for clarity.

A 1:1 disorder of the side chain was observed in the crystal. CCDC 2379089 contains the supplementary crystallographic data.

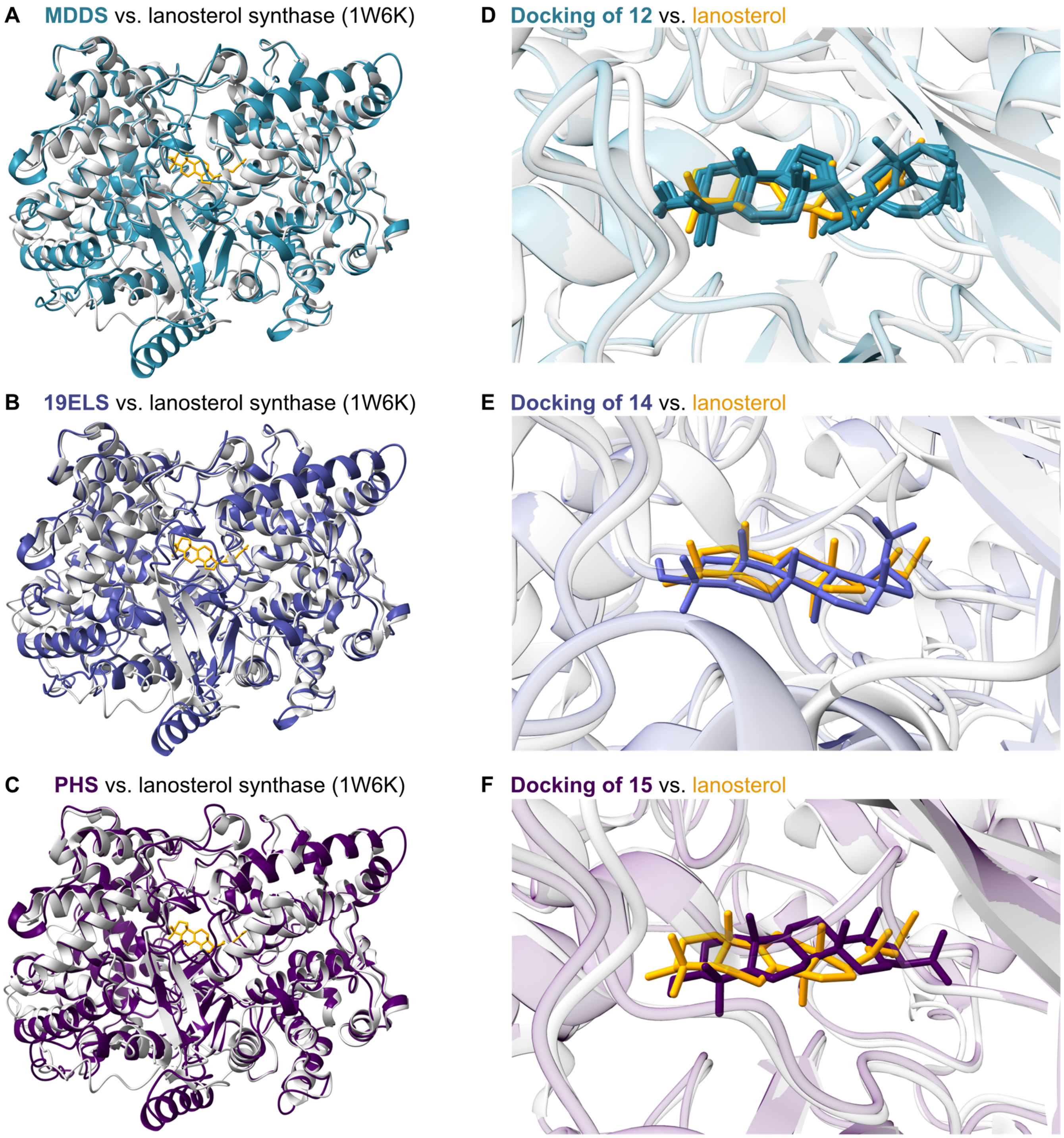

Figure S19. AlphaFold2 models of MDDS (OSC4) (A), 19ELS (OSC5) (B) and PHS (OSC6) (C) with docked products (3*S*,13*S*)-malabarica-17,21-diene-3β,14-diol (**12**) (D), 19-*epi*-lupeol (**14**) (E), or protostahopenol (**15**), respectively, in comparison to the crystal structure of lanosterol synthase (1W6K) (grey) containing lanosterol (orange).[18]

For (3*S*,13*S*)-malabarica-17,21-diene-3β,14-diol (**12**), both possible C14 epimers were docked. Due to the flexible side chain, multiple similar docking poses were obtained (five in total for both C14 epimers).

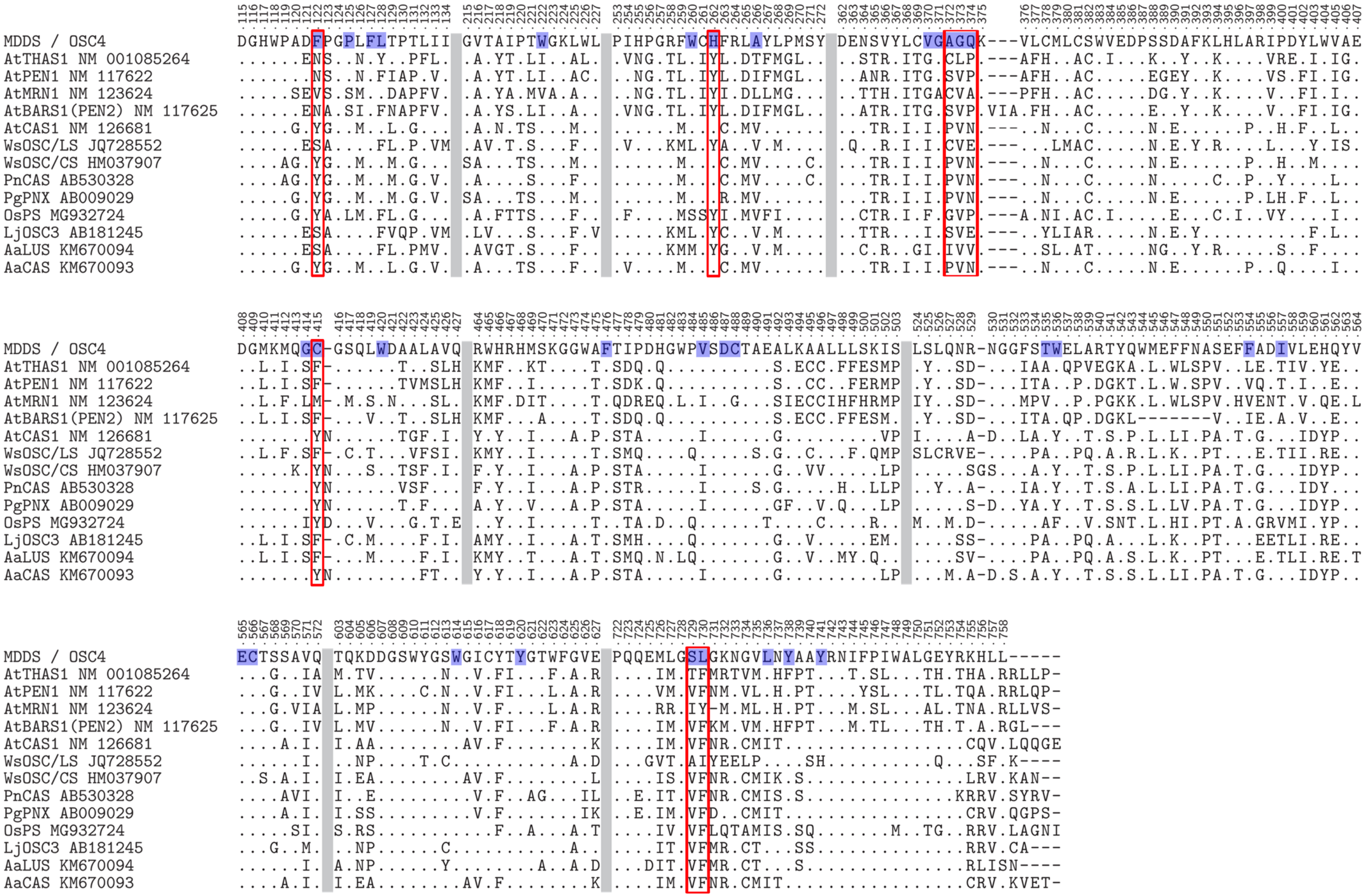

Figure S20. Multiple sequence alignment of MDDS (OSC4), OSCs with a similar product spectrum, and selected representative OSCs.

Residues within 5 Å of the docked product are highlighted in blue. Non-conserved residues in the active site which were selected for mutagenesis are highlighted with red boxes. Conserved residues and regions outside of the active site are omitted for clarity. The numbering refers to the sequence of MDDS.

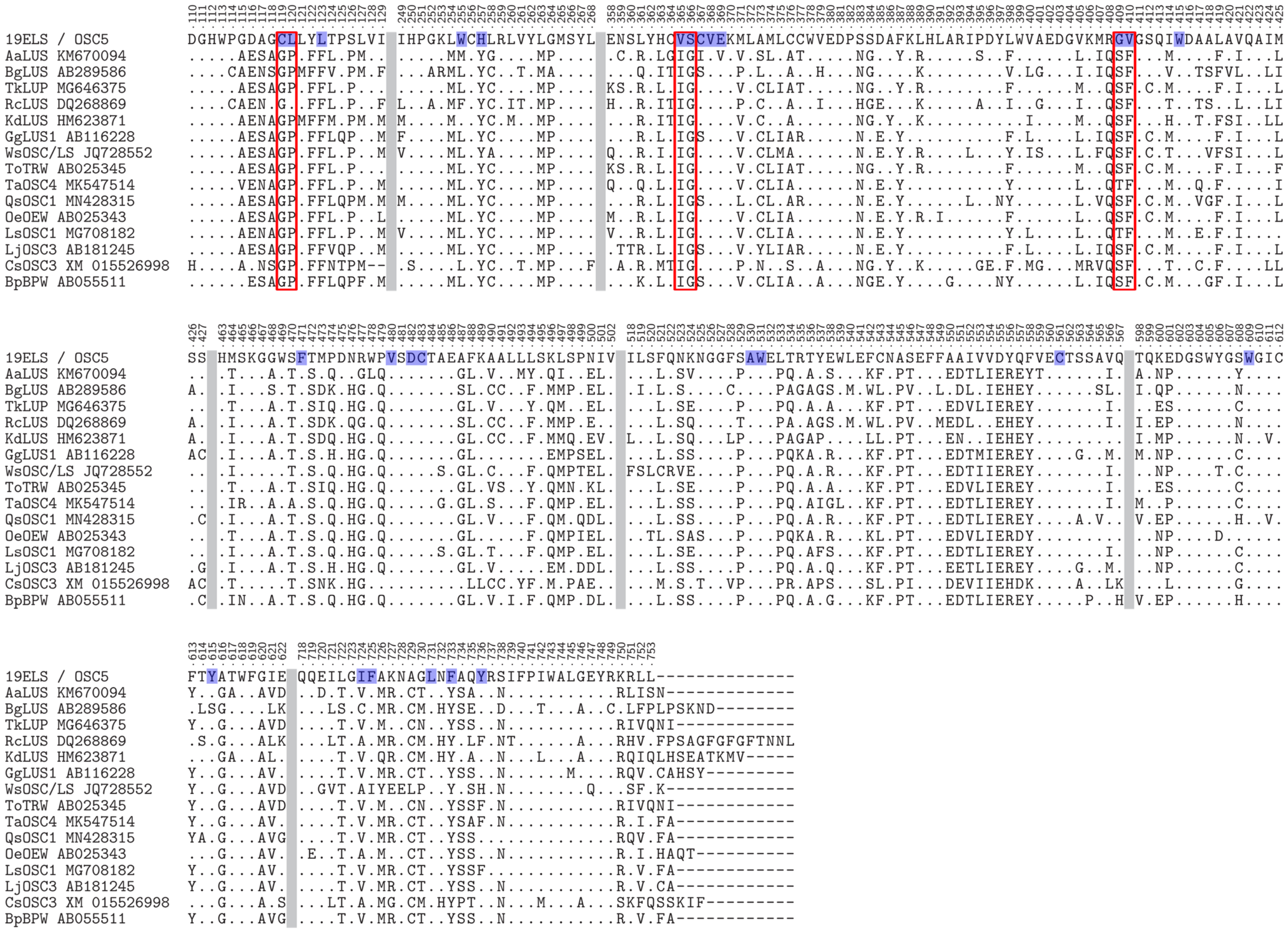

Figure S21. Multiple sequence alignment of 19ELS (OSC5) and OSCs with a similar product spectrum.

Residues within 5 Å of the docked product are highlighted in blue. Non-conserved residues in the active site which were selected for mutagenesis are highlighted with red boxes. Conserved residues and regions outside of the active site are omitted for clarity. The numbering refers to the sequence of 19ELS.

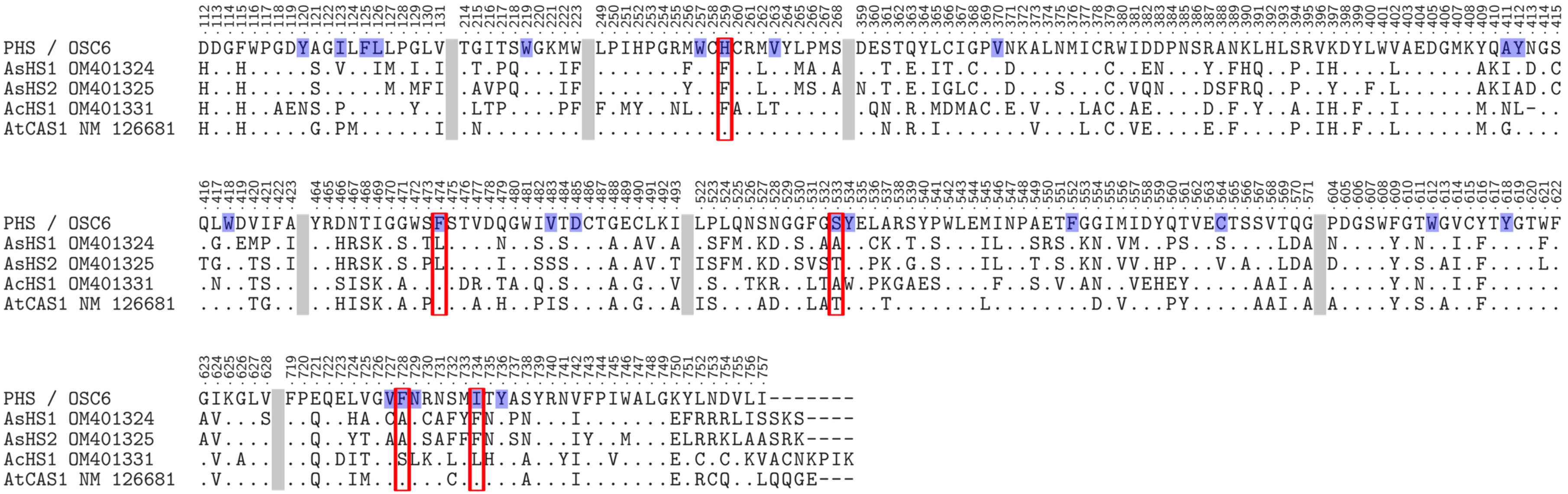

Figure S22. Multiple sequence alignment of PHS (OSC6) and OSCs with a similar product spectrum.

Residues within 5 Å of the docked product are highlighted in blue. Non-conserved residues in the active site which were selected for mutagenesis are highlighted with red boxes. Conserved residues and regions outside of the active site are omitted for clarity. The numbering refers to the sequence of PHS.

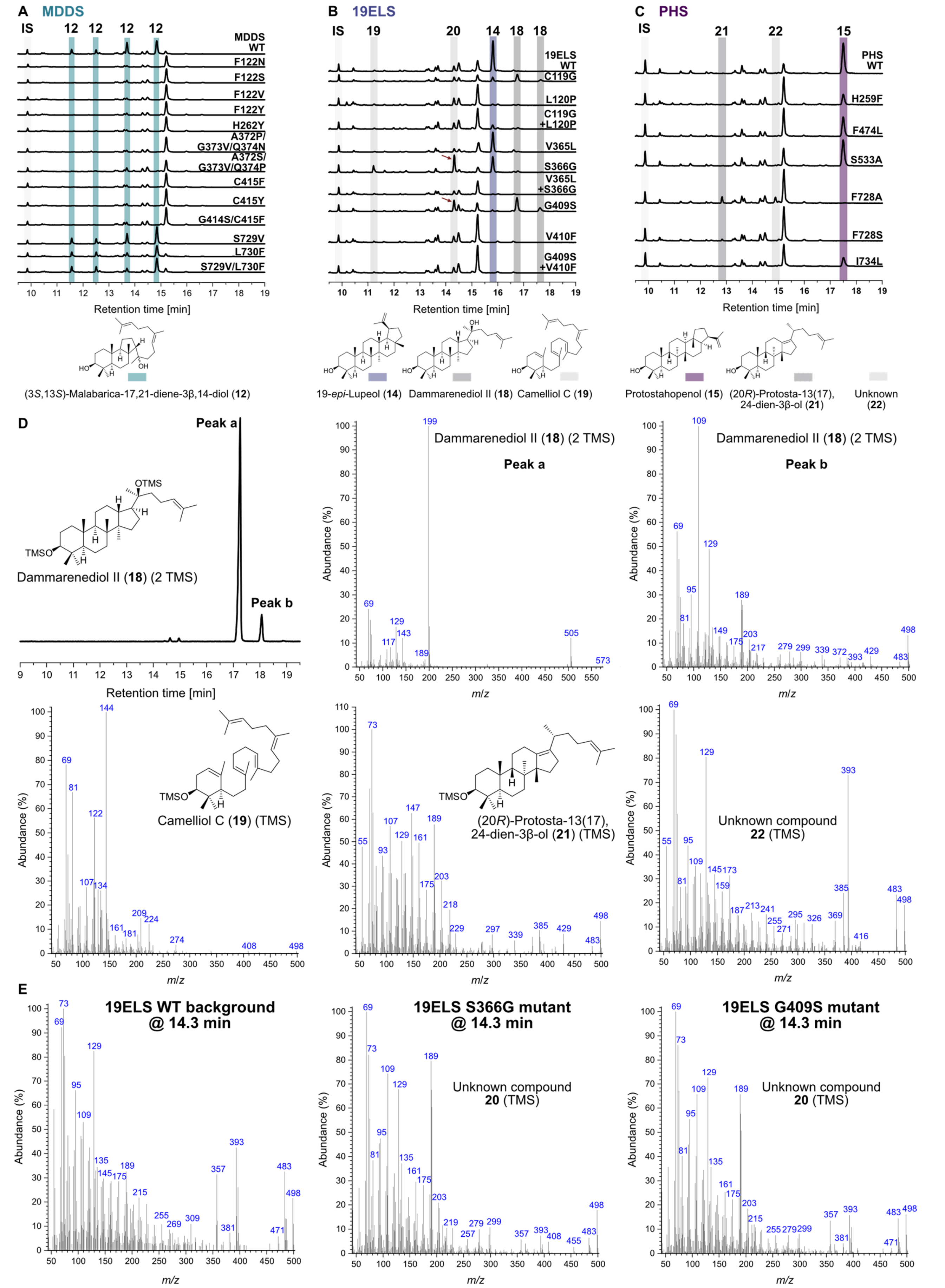

Figure S23. Detailed OSC mutagenesis results.

Representative GC-MS TIC chromatograms of mutants of MDDS (A), 19ELS (B), and PHS (C) together with identified compounds. D) Mass spectra (EI) of compounds from mutant OSCs (TMS derivatives). Pure dammarenediol II (**18**) shows two peaks a and b upon saponification, derivatisation, and GC-MS analysis. E) 19ELS mutants S366G and G409S produce an unknown compound **20** co-eluting with a background peak at 14.3 min as judged by a change in mass spectra.

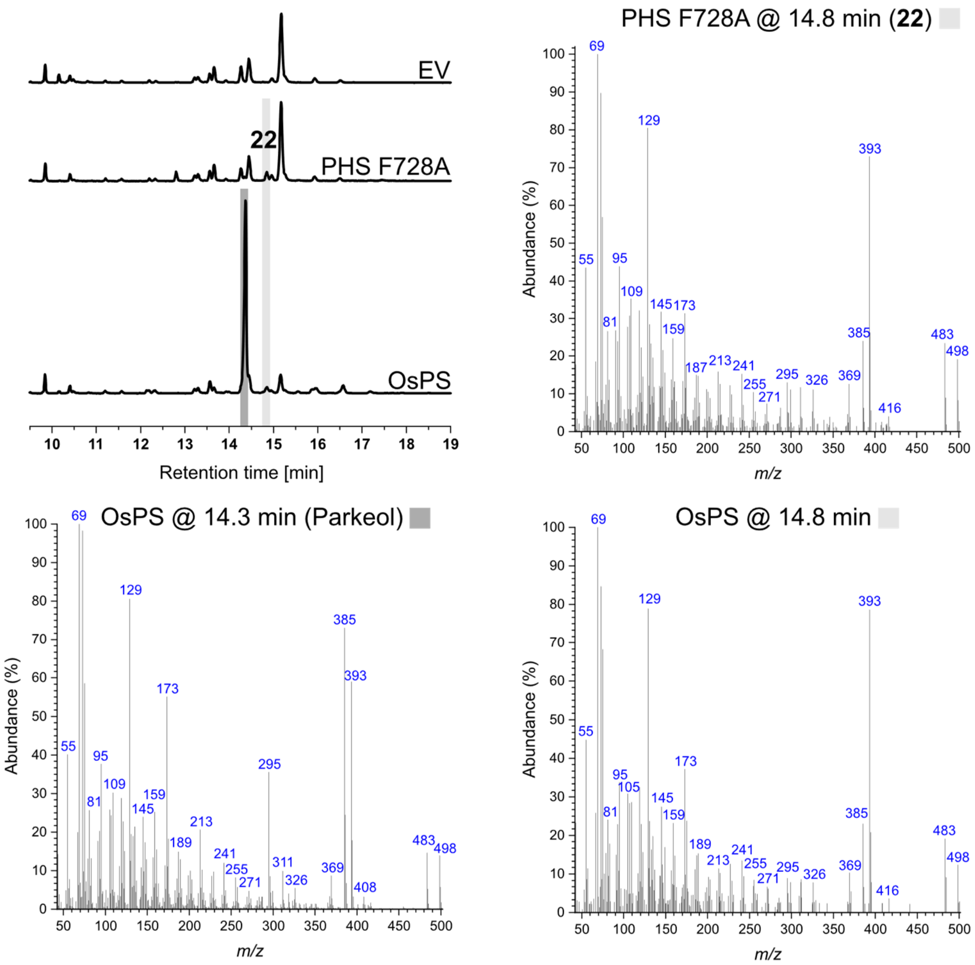

Figure S24. Unknown compound **22** produced by PHS mutant F728A matches with a minor unknown byproduct of *Oryza sativa* parkeol synthase (OsPS)[11] in terms of retention time and mass spectrum.

The mass spectrum of **22** (TMS ether) shows strong similarities to the mass spectrum of parkeol (TMS ether) at 14.3 min.

Please note that there are inconsistencies in the literature regarding the mass spectrum of parkeol (TMS ether): The mass spectrum reported by Xue *et al.*[32] is in excellent agreement with the spectrum that we observed for the main peak at 14.3 min; in contrast, the mass spectra by Pearson et al.[33] and Banta *et al.*[34] fit much better to the minor byproduct at 14.8 min and compound **22**.

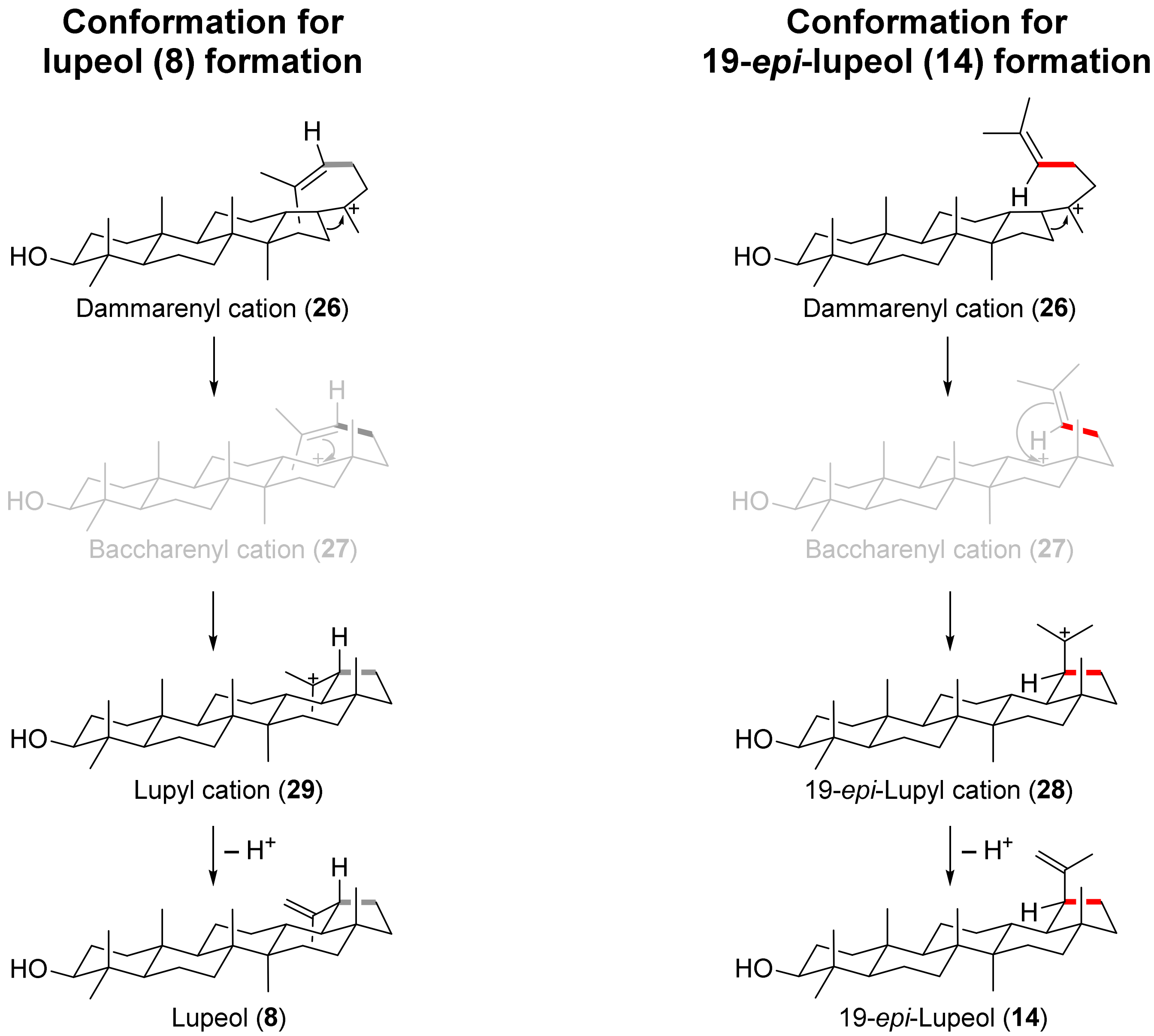

Figure S25. Proposed mechanism for 19-*epi*-lupeol (**14**) formation involving a flipped orientation (highlighted in red) of the terminal isobutenyl moiety in comparison to lupeol (**8**) formation.

The formation of pentacyclic cations likely proceeds in an asynchronous concerted manner rather than via the secondary carbocations shown in gray.

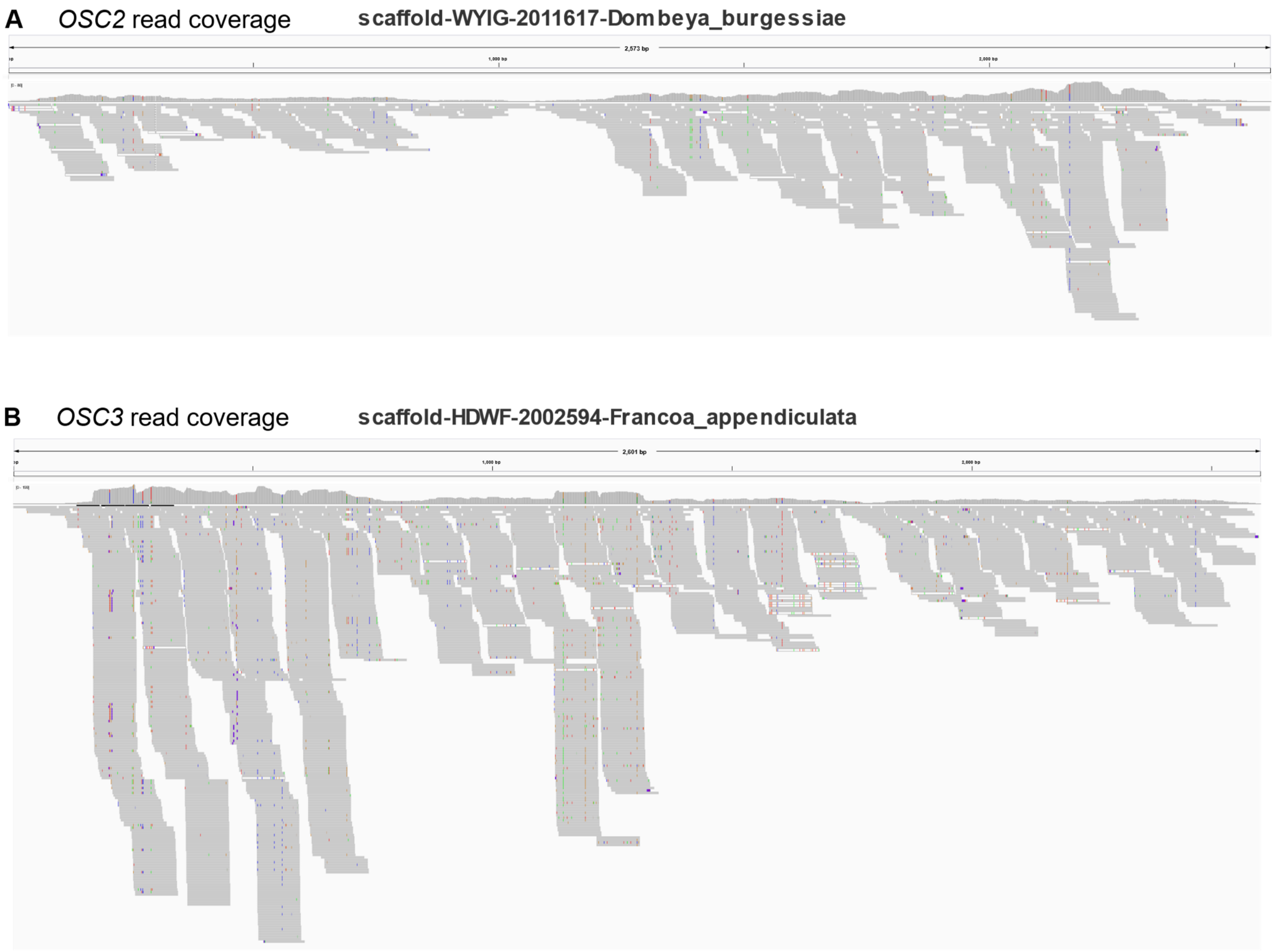

Figure S26. Read coverage of the *OSC2* and *OSC3* transcripts in the One Thousand Plant Transcriptomes dataset.

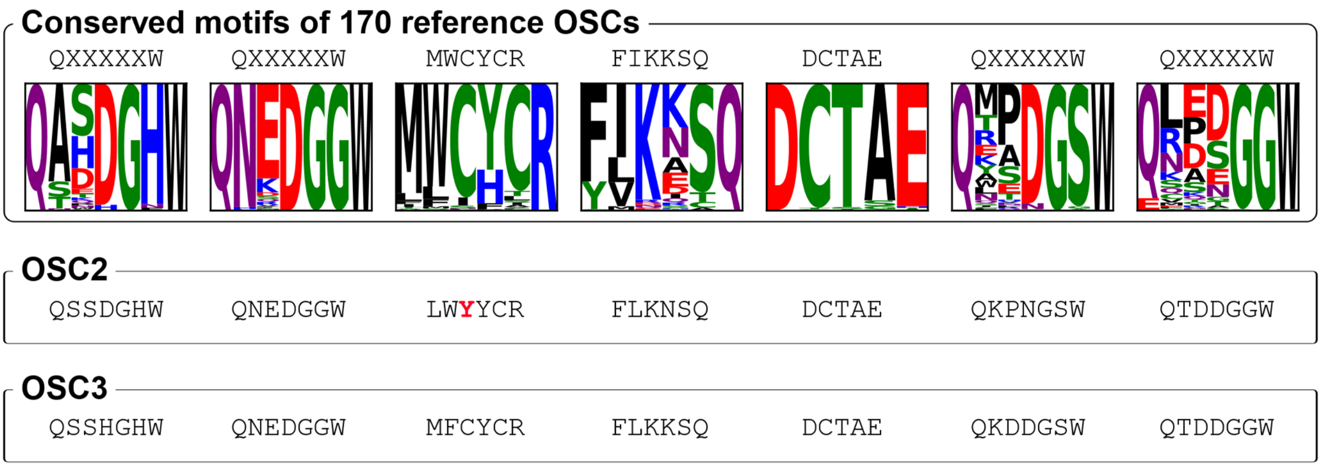

Figure S27. Comparison of conserved motifs[3,27] between the 170 reference OSCs[3] and OSC2/OSC3.
For OSC2, the MWCYCR motif contains a highly unusual C->Y mutation (red, bold) that is not seen for any of the reference OSCs.

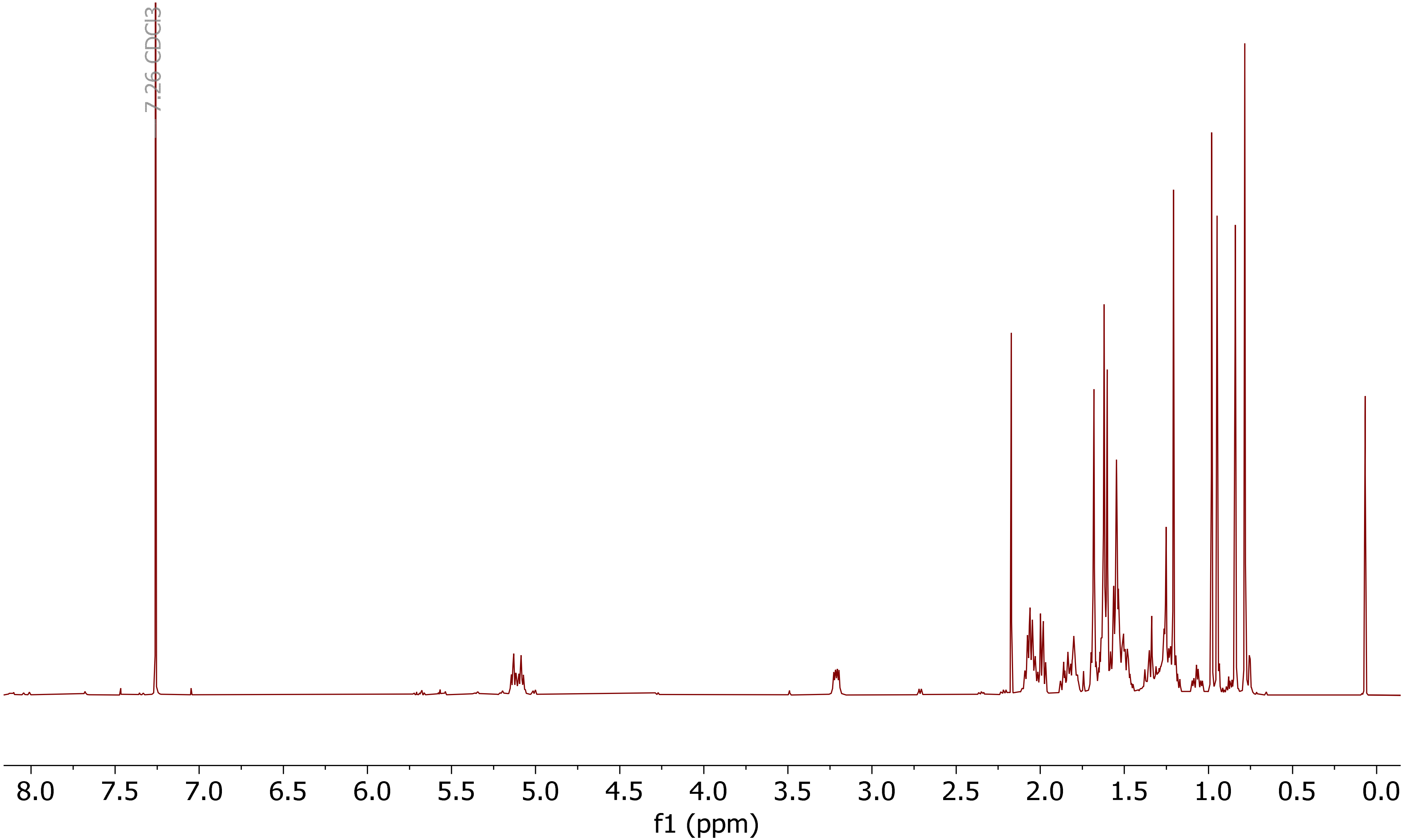

Figure S28. ^{1}H spectrum of (3*S*,13*S*)-malabarica-17,21-diene-3β,14-diol (**12**) ($CDCl_3$, 500 MHz, 298 K).

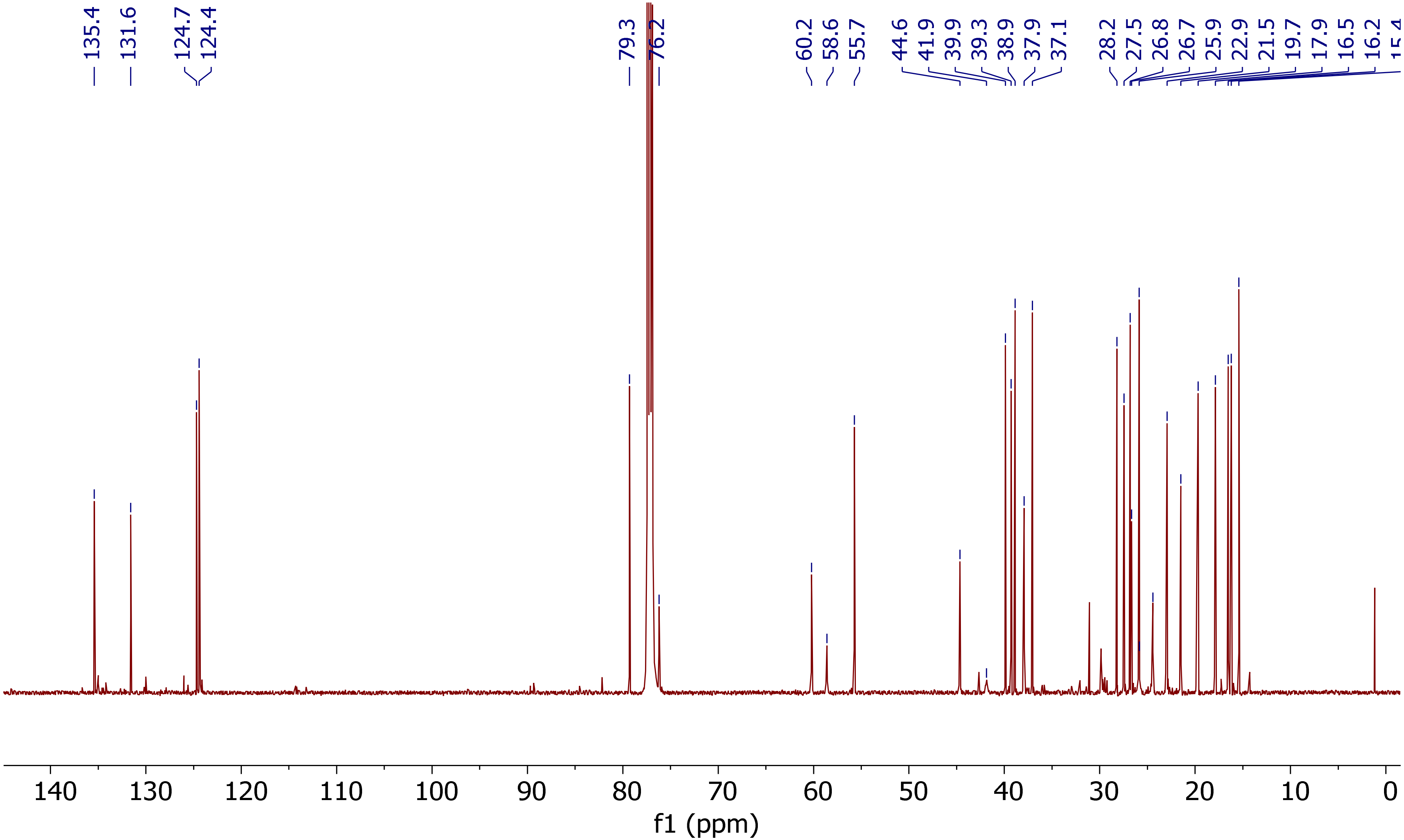

Figure S29. ^{13}C spectrum of (3*S*,13*S*)-malabarica-17,21-diene-3β,14-diol (**12**) ($CDCl_3$, 151 MHz, 298 K).

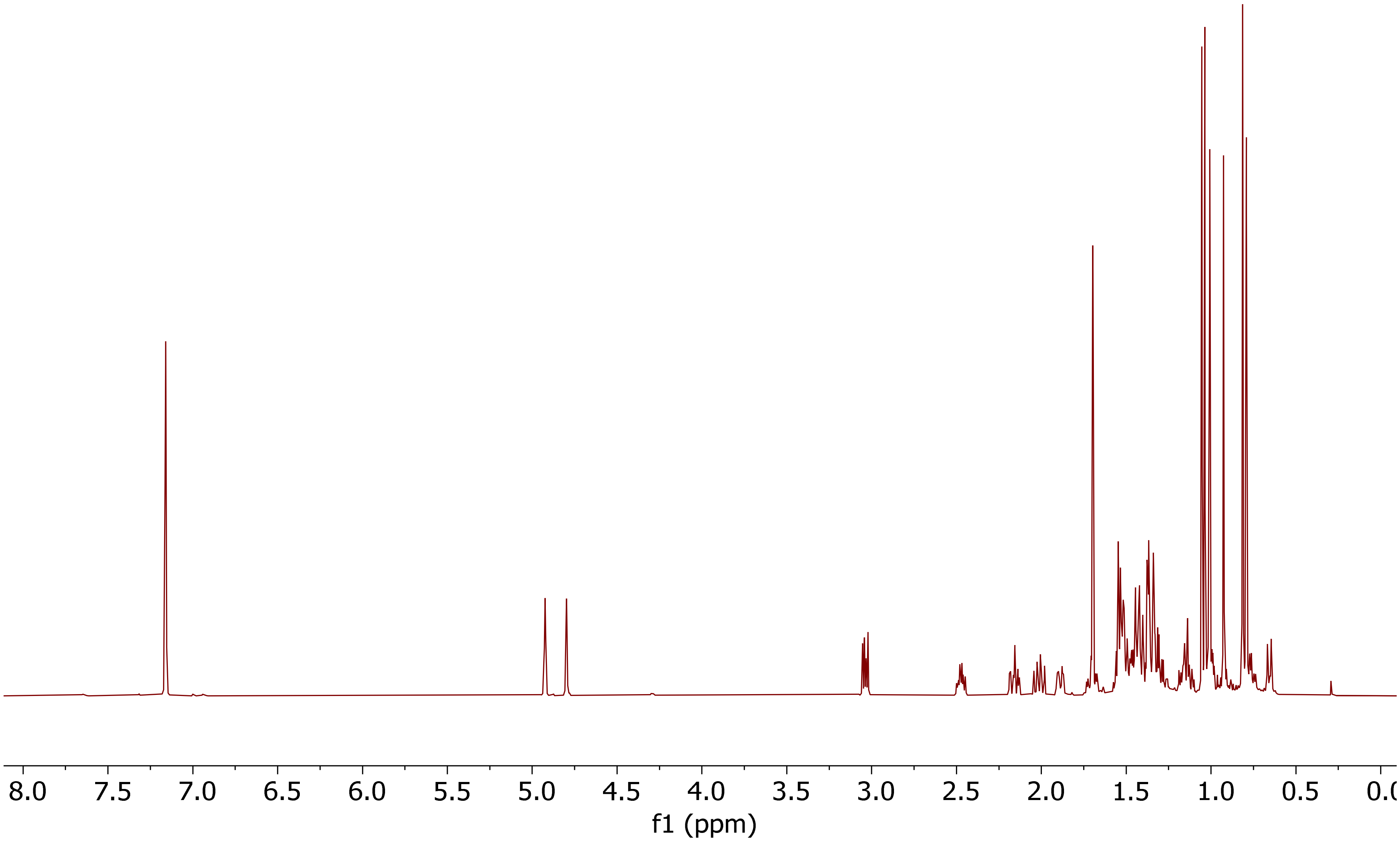

Figure S30. ^{1}H spectrum of 19-*epi*-lupeol (**14**) (C_6D_6, 500 MHz, 298 K).

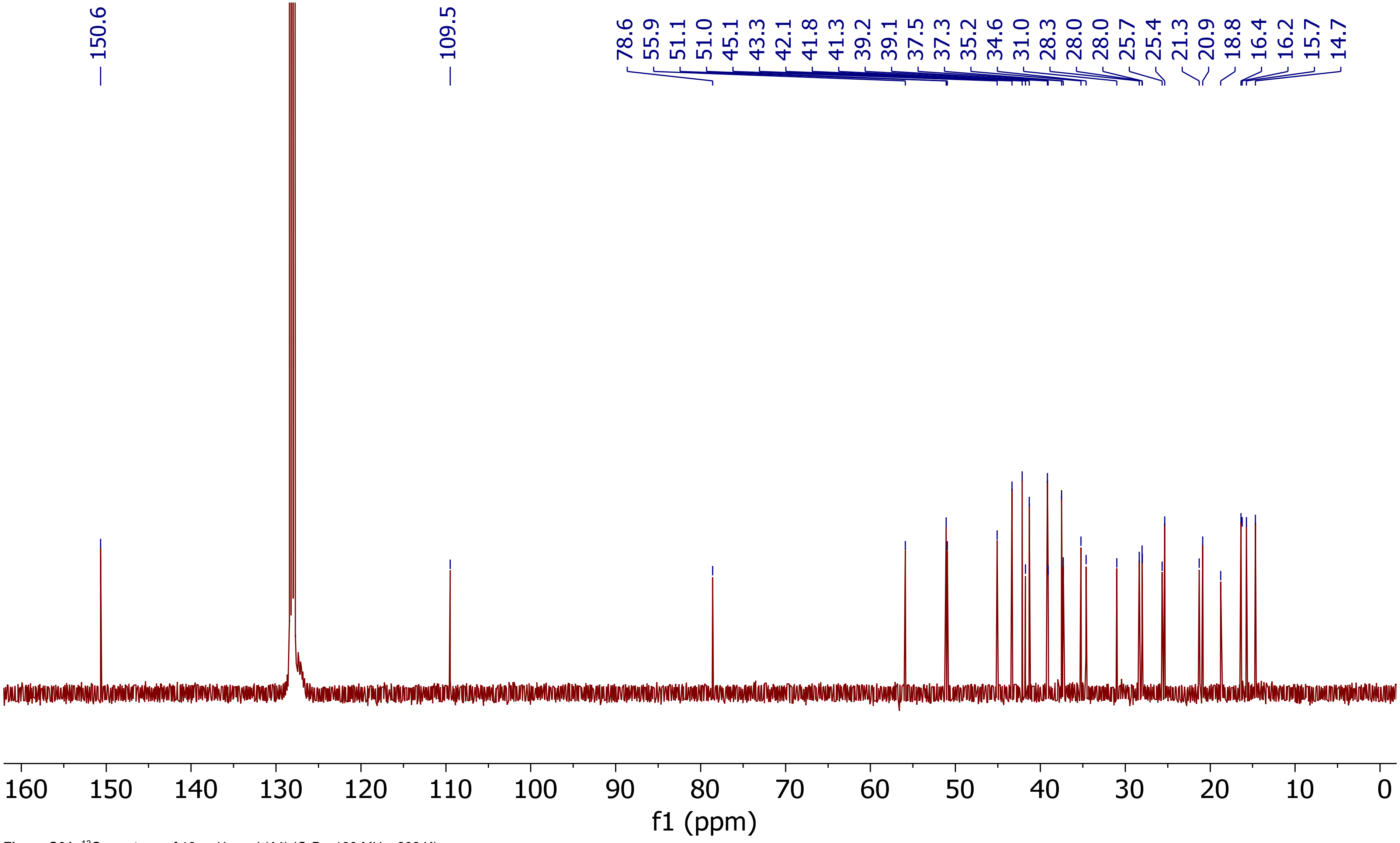

Figure S31. ^{13}C spectrum of 19-*epi*-lupeol (**14**) (C_6D_6, 100 MHz, 298 K).

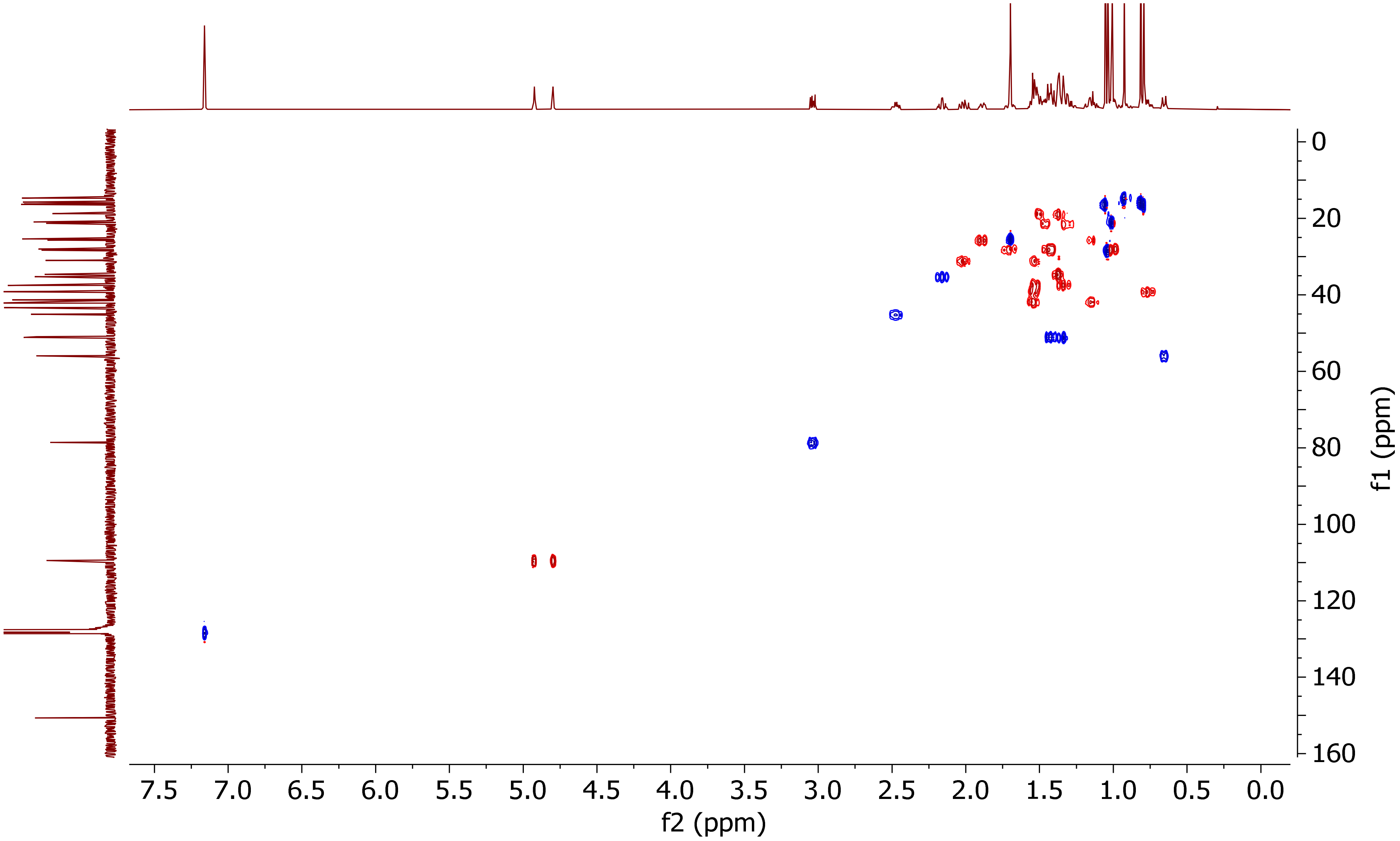

Figure S32. HSQC spectrum of 19-*epi*-lupeol (**14**) (C_6D_6, 500 MHz, 298 K).

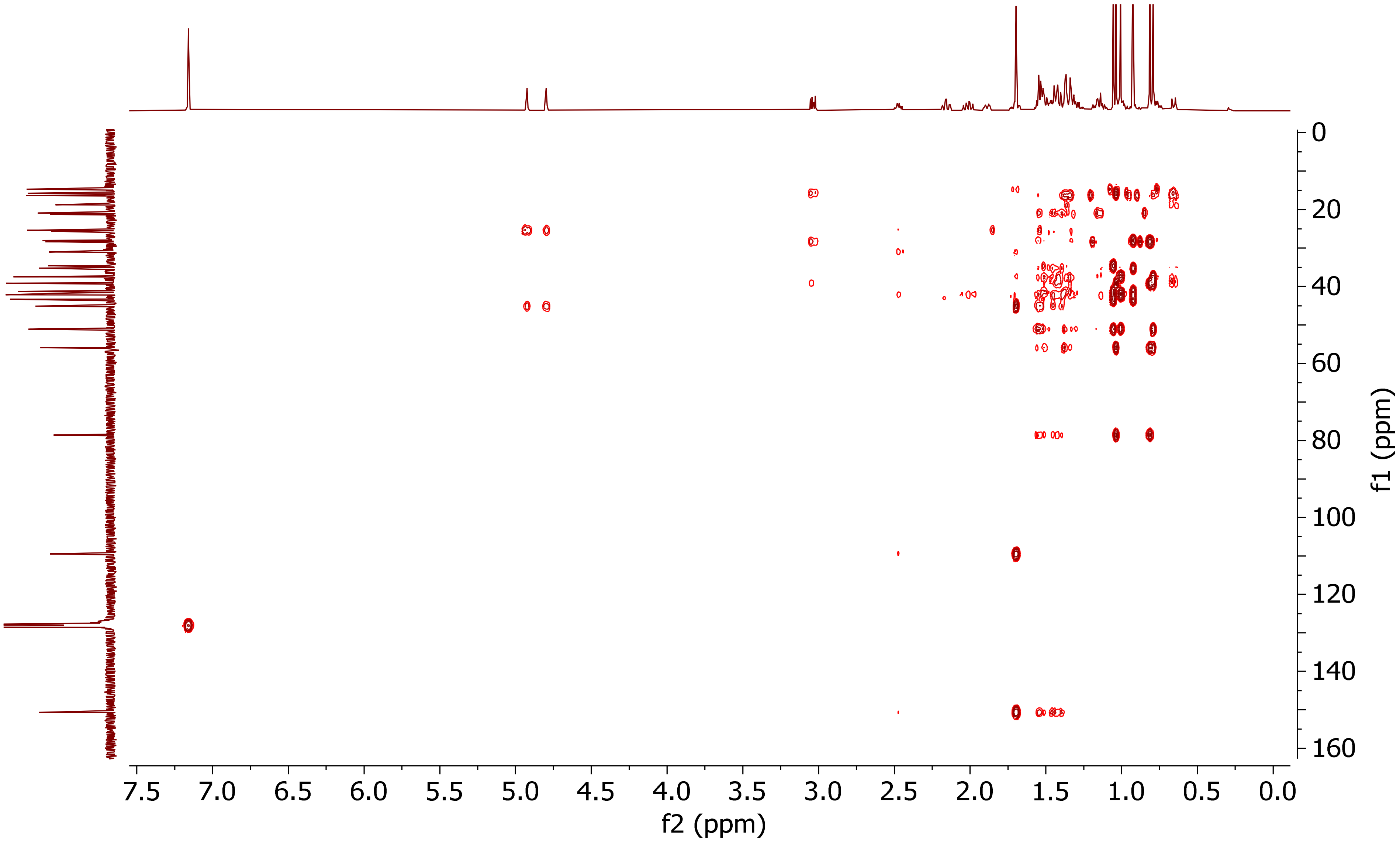

Figure S33. HMBC spectrum of 19-*epi*-lupeol (**14**) (C_6D_6, 500 MHz, 298 K).

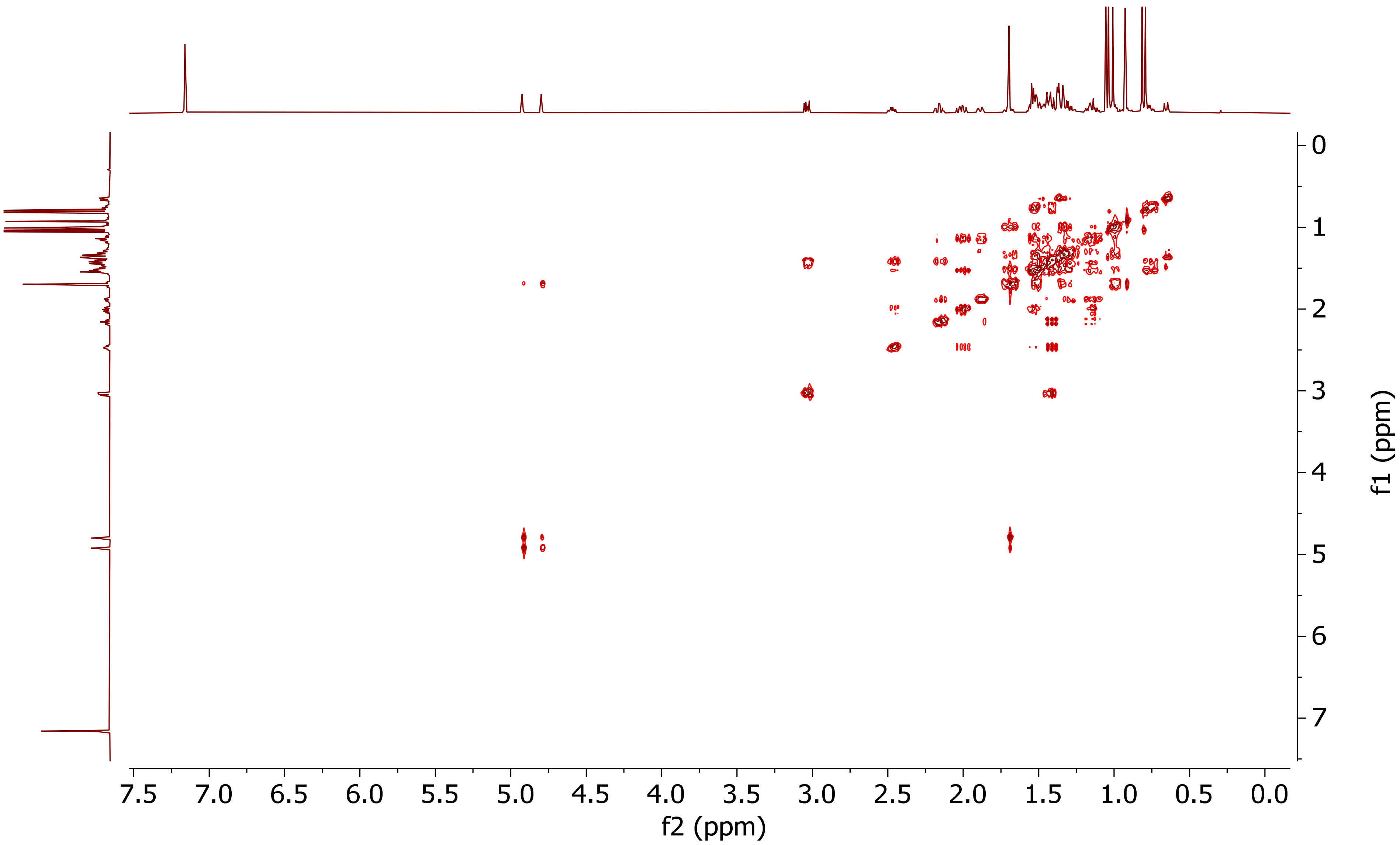

Figure S34. COSY spectrum of 19-*epi*-lupeol (**14**) (C_6D_6, 500 MHz, 298 K).

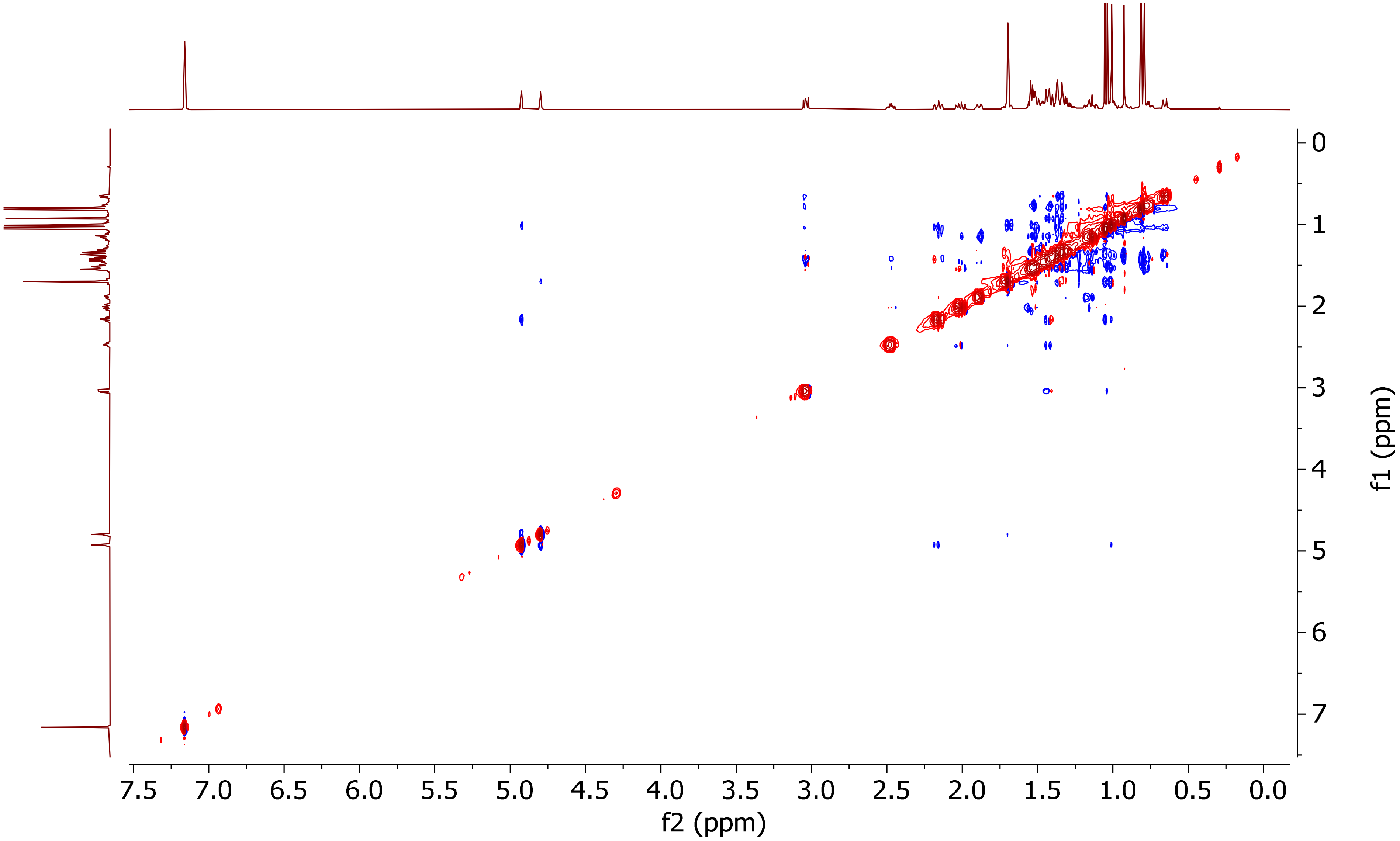

Figure S35. NOESY spectrum of 19-*epi*-Lupeol (**14**) (C_6D_6, 500 MHz, 298 K).

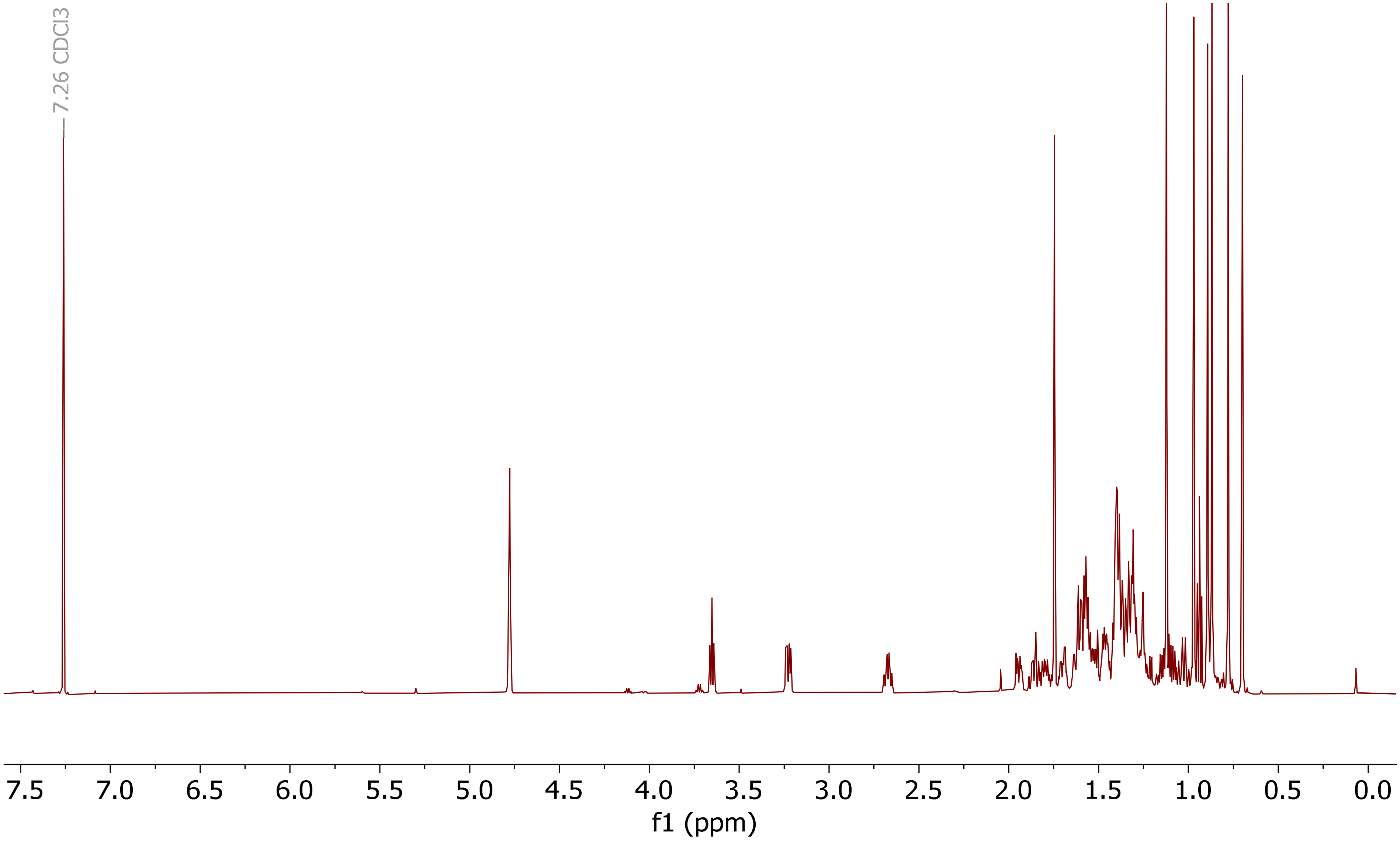

Figure S36. ^{1}H spectrum of protostahopenol (**15**) ($CDCl_3$, 600 MHz, 298 K).

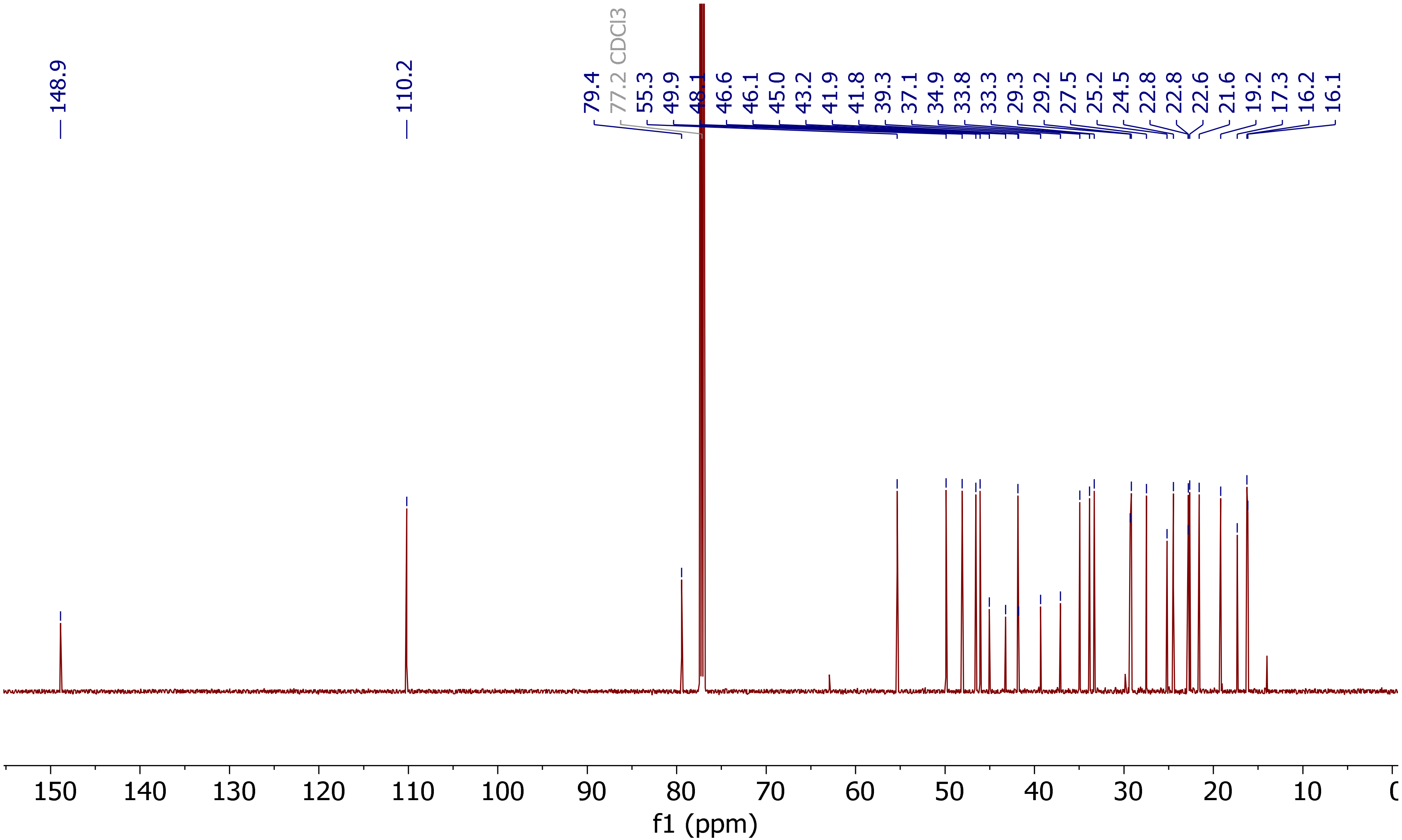

Figure S37. ^{13}C spectrum of protostahopenol (**15**) ($CDCl_3$, 151 MHz, 298 K).

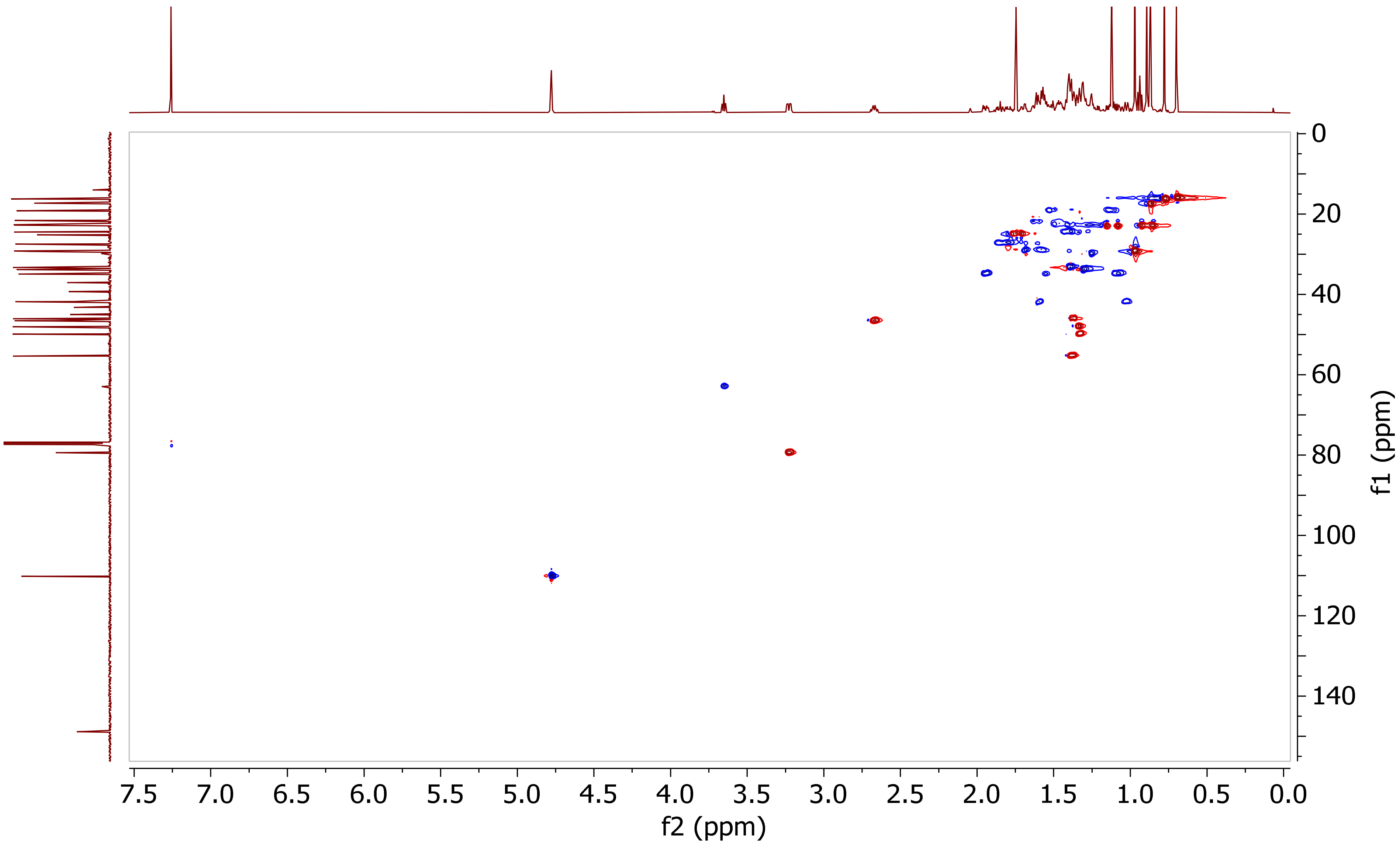

Figure S38. HSQC spectrum of protostahopenol (**15**) ($CDCl_3$, 600 MHz, 298 K).

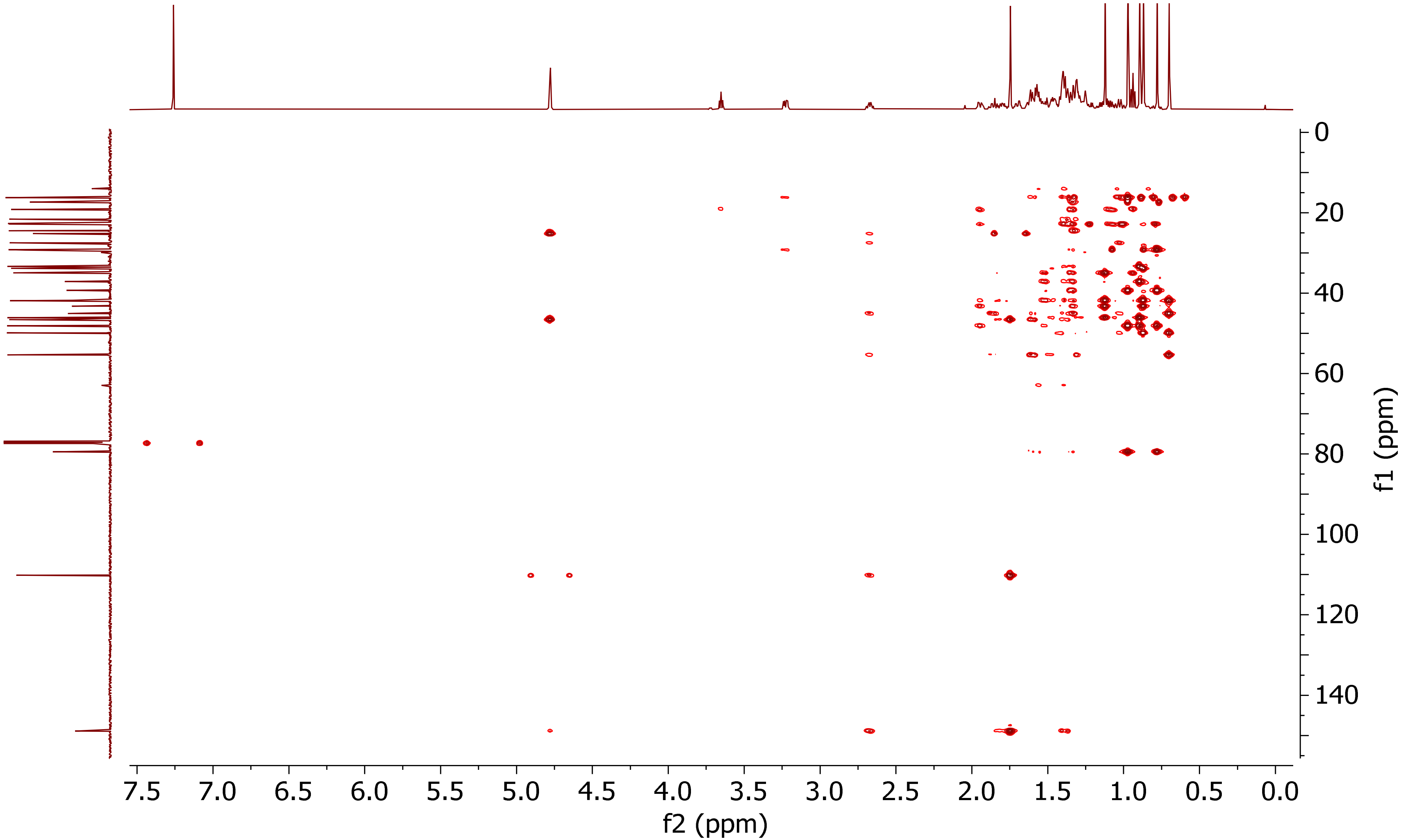

Figure S39. HMBC spectrum of protostahopenol (**15**) ($CDCl_3$, 600 MHz, 298 K).

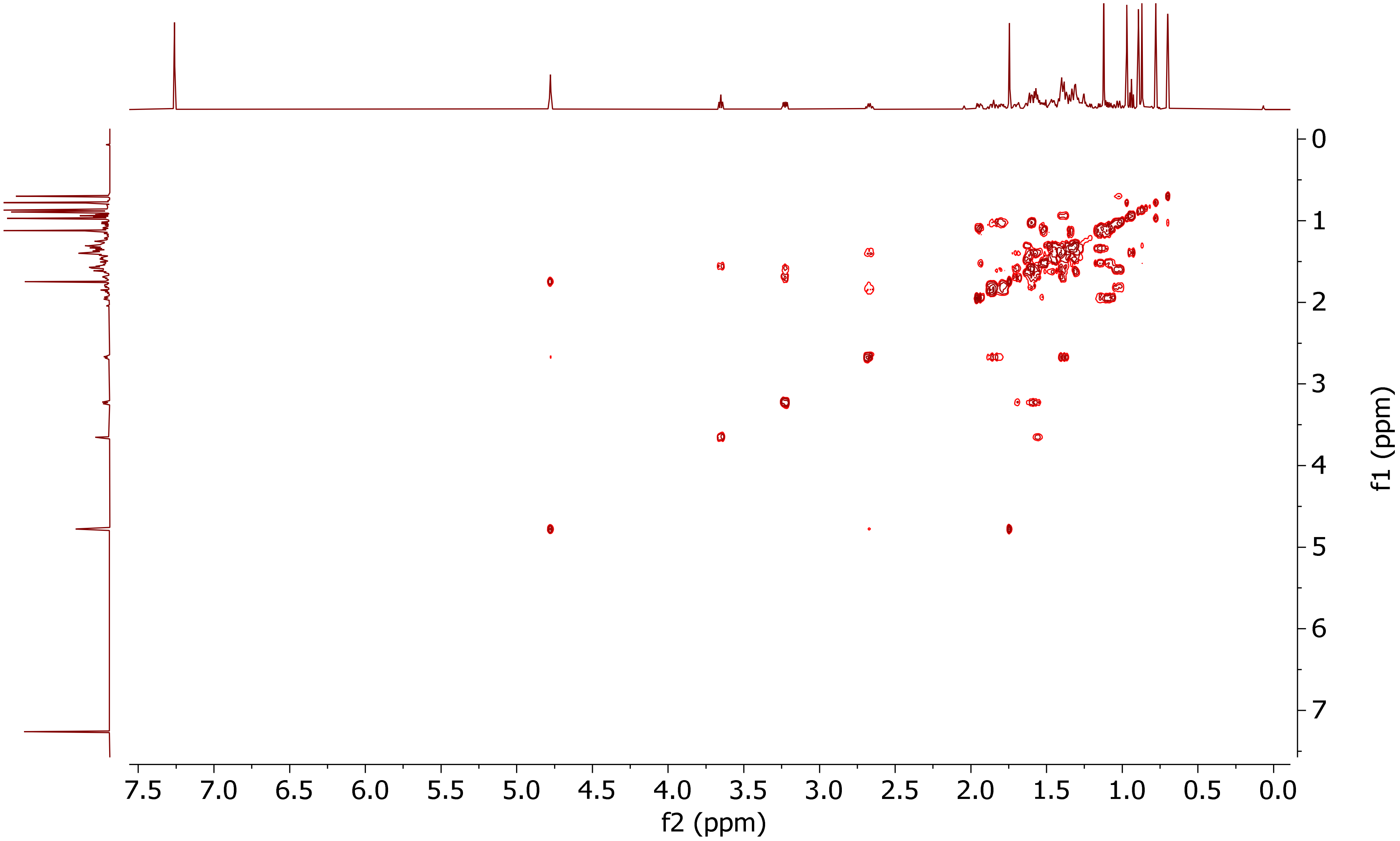

Figure S40. COSY spectrum of protostahopenol (**15**) ($CDCl_3$, 600 MHz, 298 K).

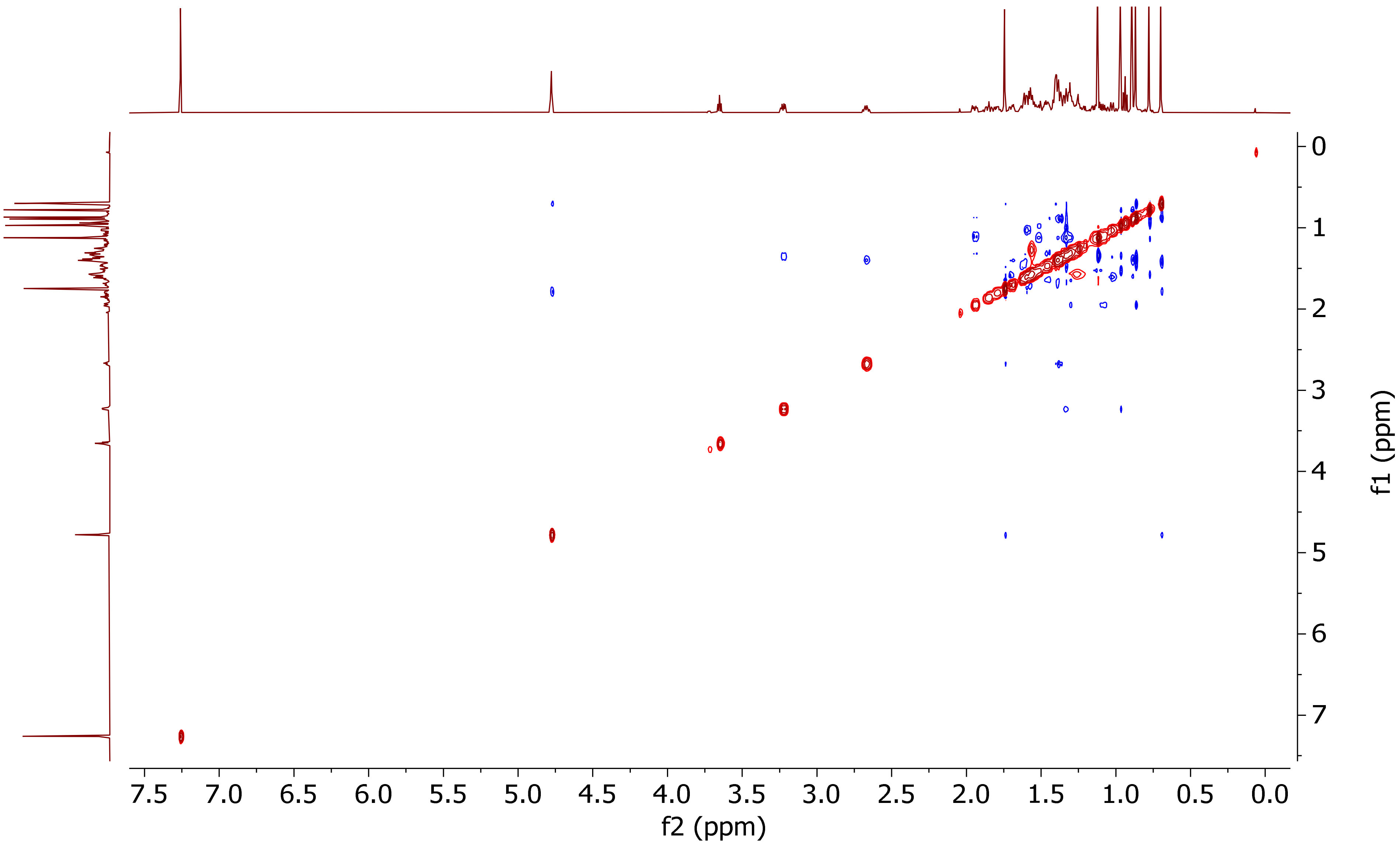

Figure S41. NOESY spectrum of protostahopenol (**15**) ($CDCl_3$, 600 MHz, 298 K).

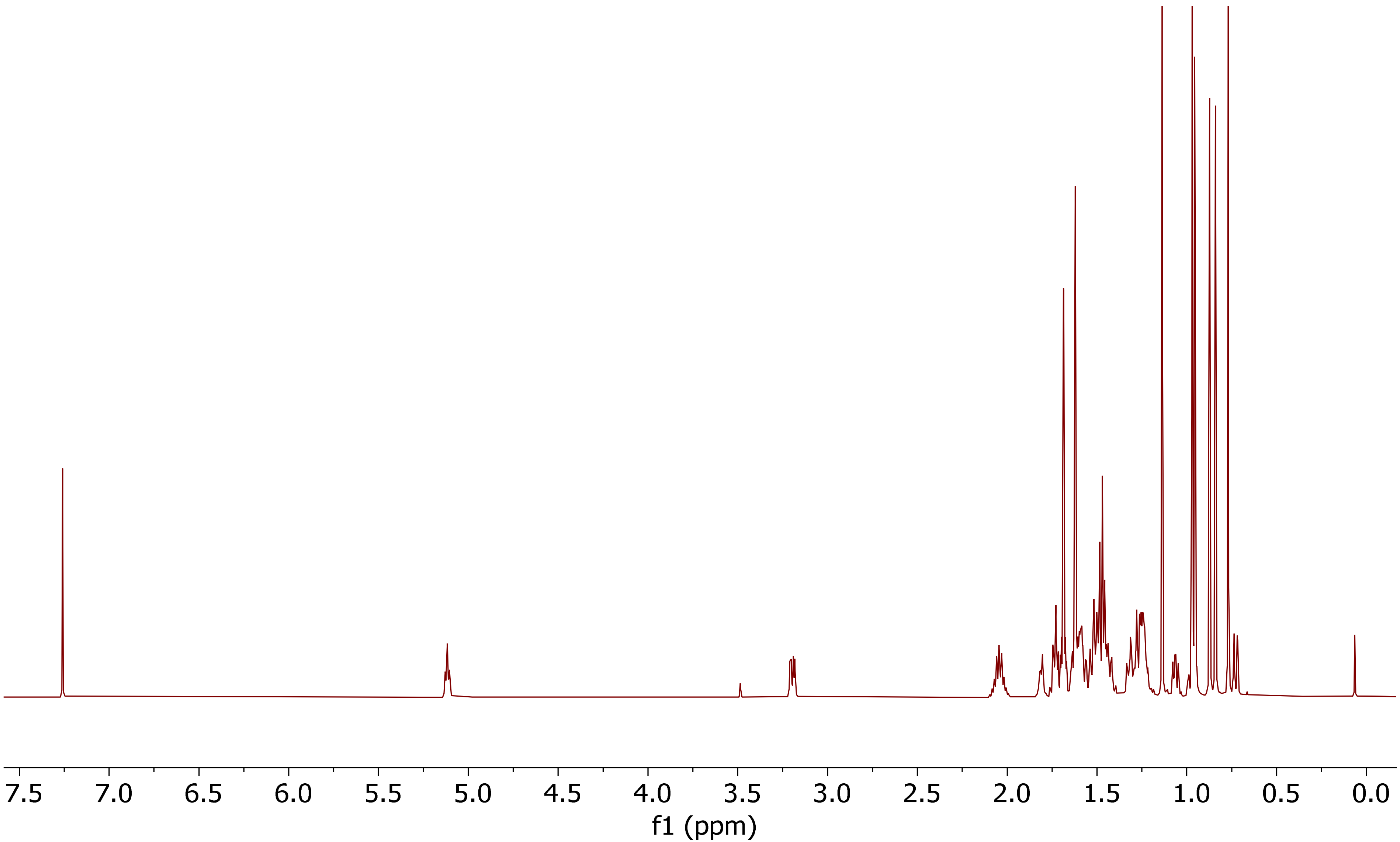

Figure S42. ^{1}H spectrum of dammarenediol II (**18**) ($CDCl_3$, 600 MHz, 298 K).

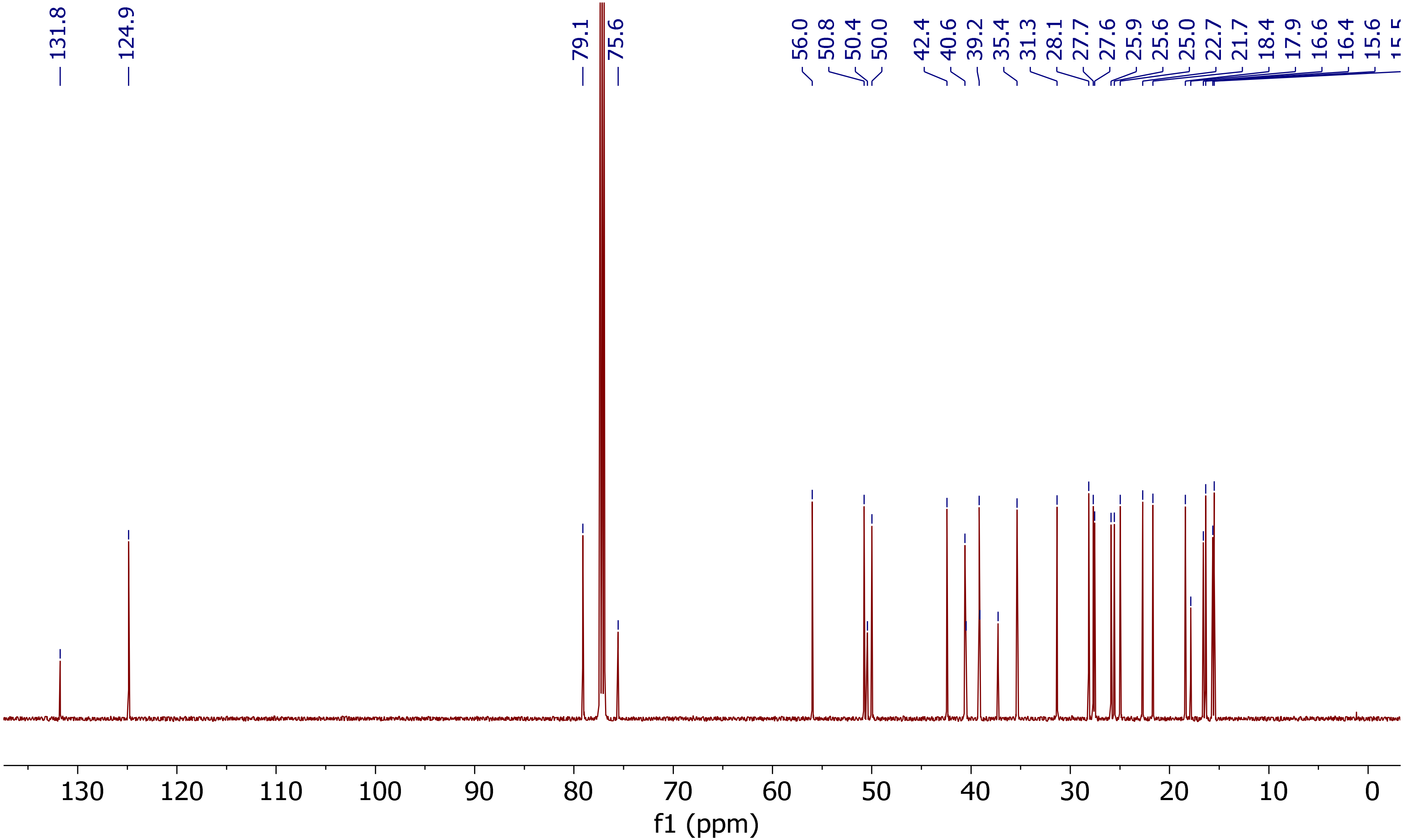

Figure S43. ^{13}C spectrum of dammarenediol II (**18**) ($CDCl_3$, 151 MHz, 298 K).

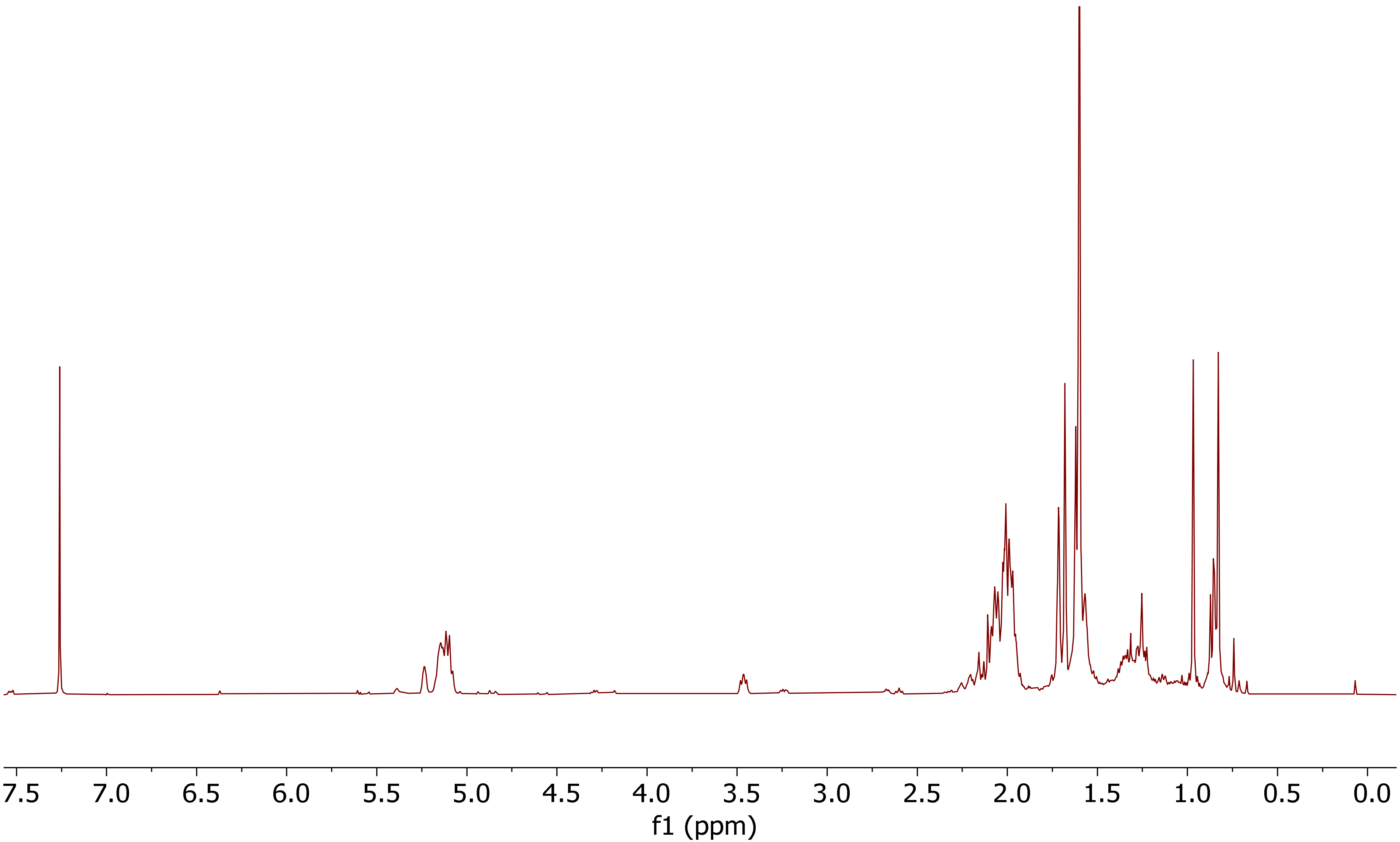

Figure S44. ^{1}H spectrum of camelliol C (**19**) ($CDCl_3$, 400 MHz, 298 K).

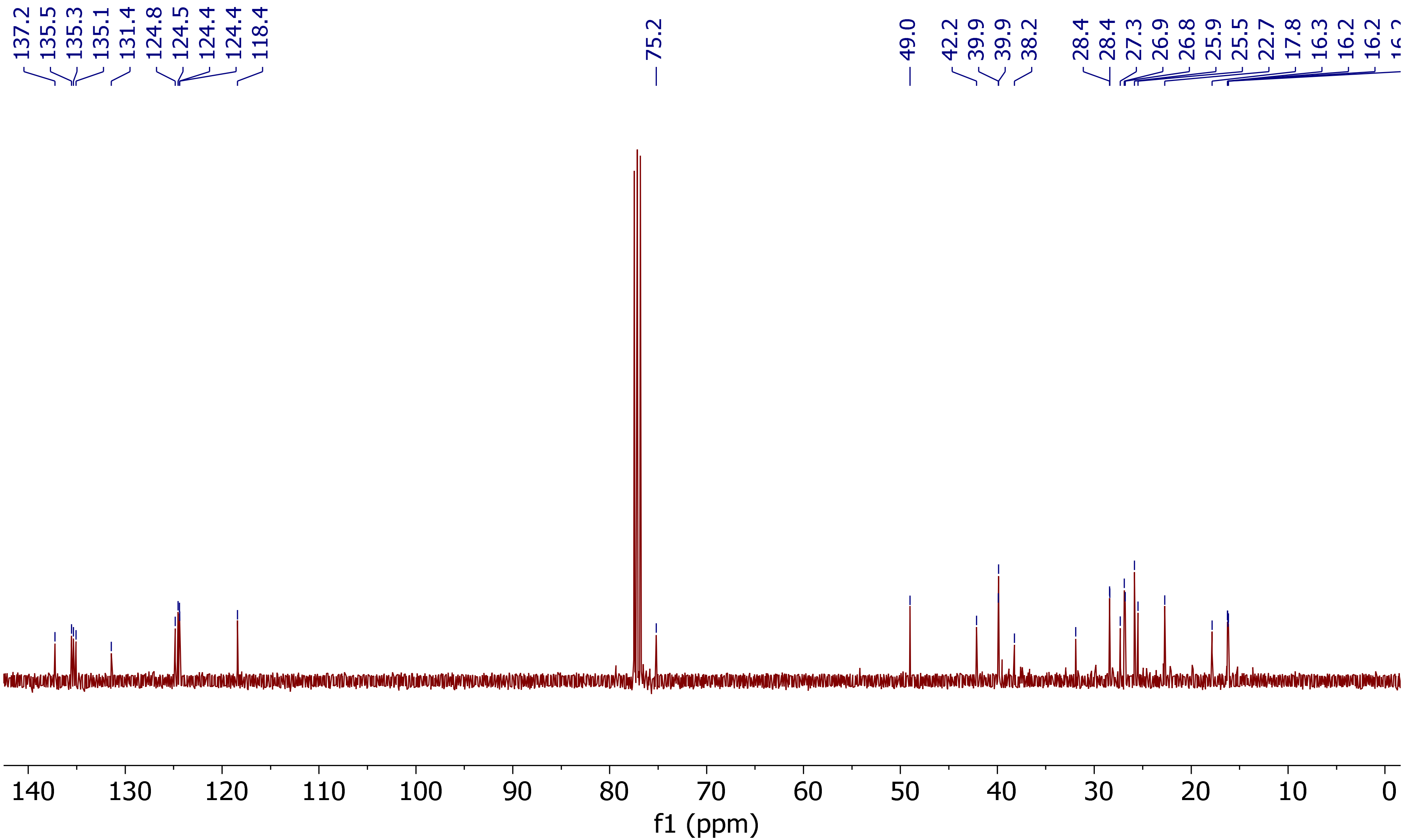

Figure S45. ^{13}C spectrum of camelliol C (**19**) ($CDCl_3$, 100 MHz, 298 K).

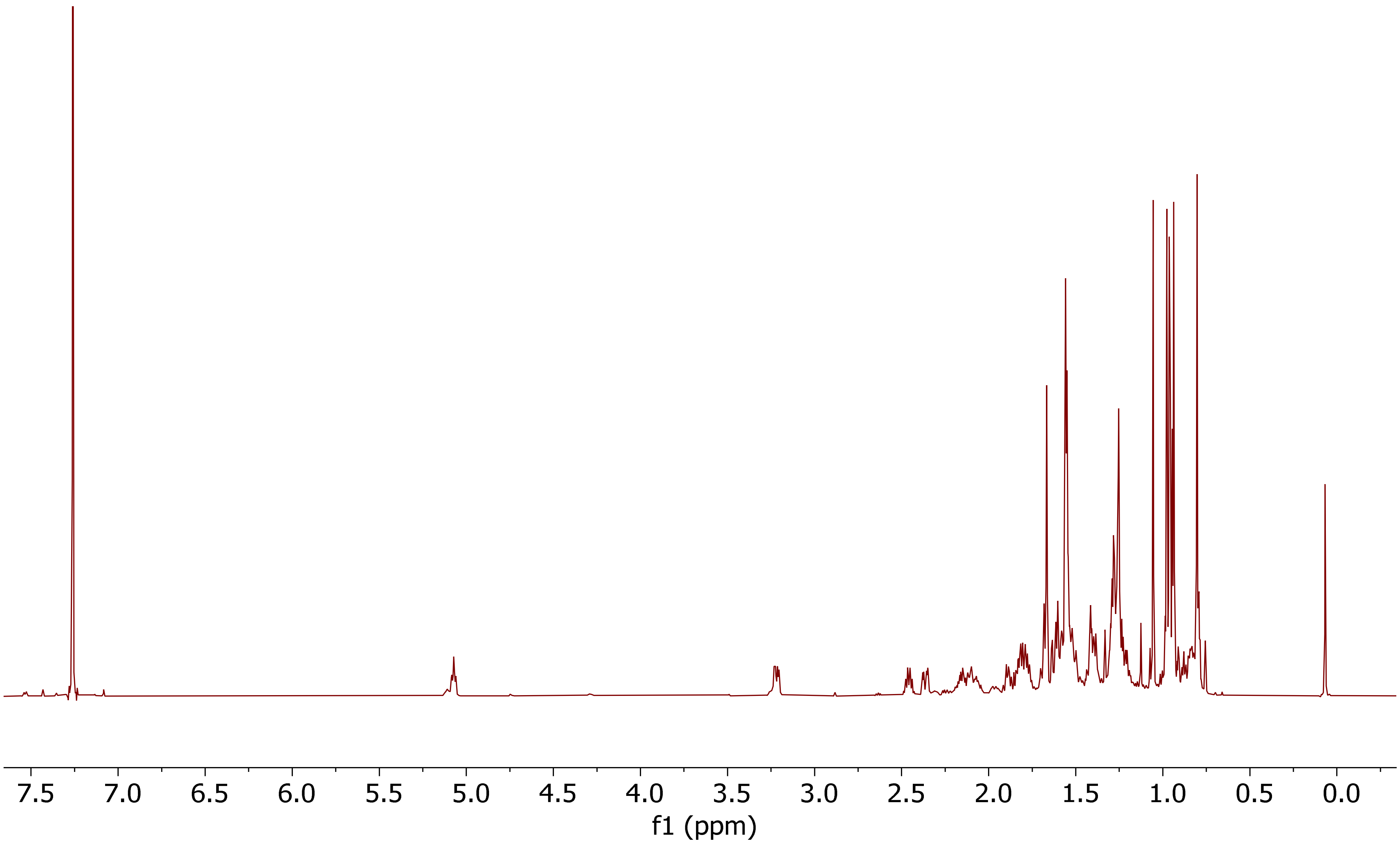

Figure S46. ^{1}H spectrum of (20*R*)-protosta-13(17),24-dien-3β-ol (**21**) ($CDCl_3$, 600 MHz, 298 K).

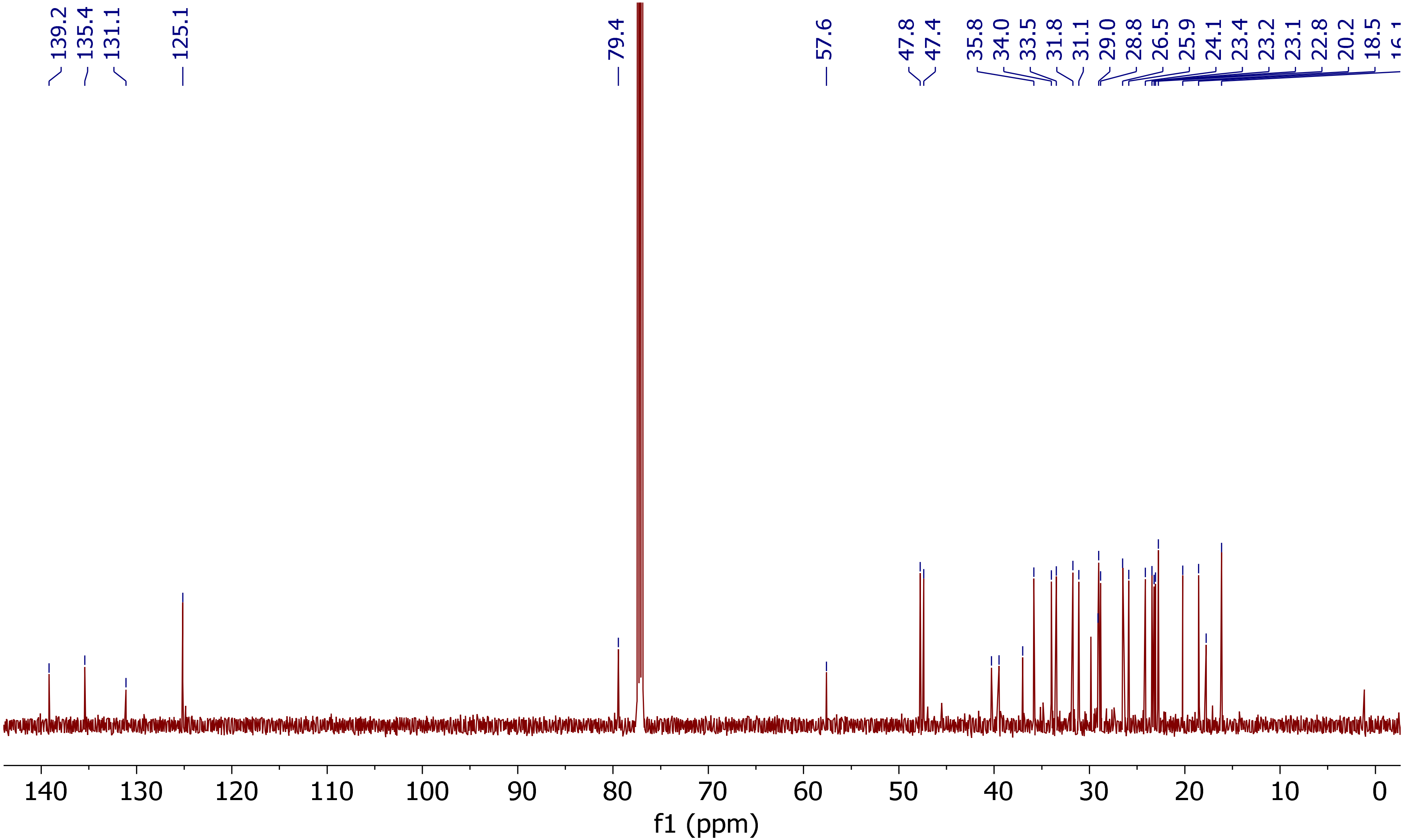

Figure S47. ^{13}C spectrum for (20*R*)-protosta-13(17),24-dien-3β-ol (**21**) ($CDCl_3$, 151 MHz, 298 K).

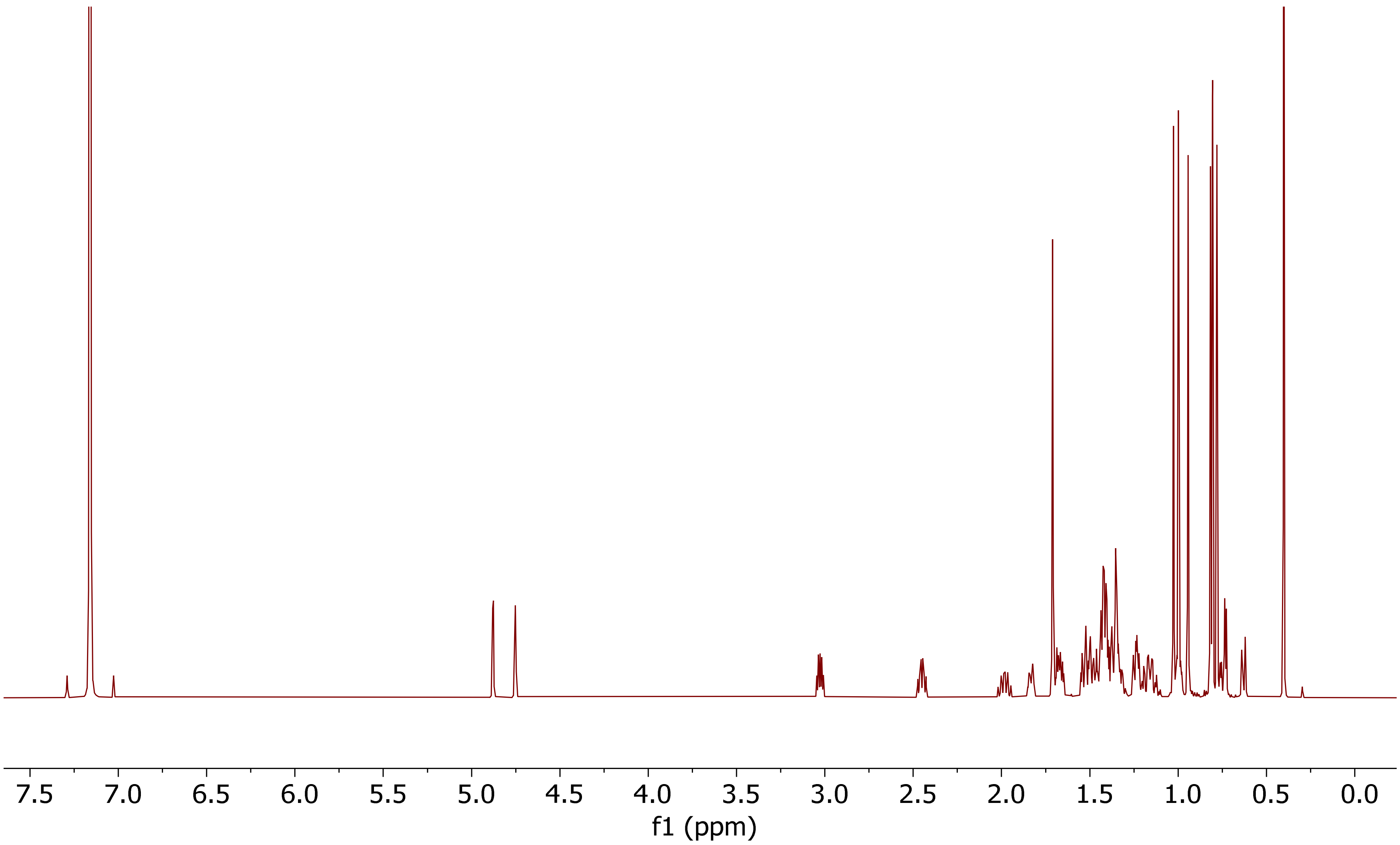

Figure S48. ^{1}H spectrum of lupeol (**8**) (C_6D_6, 600 MHz, 298 K).

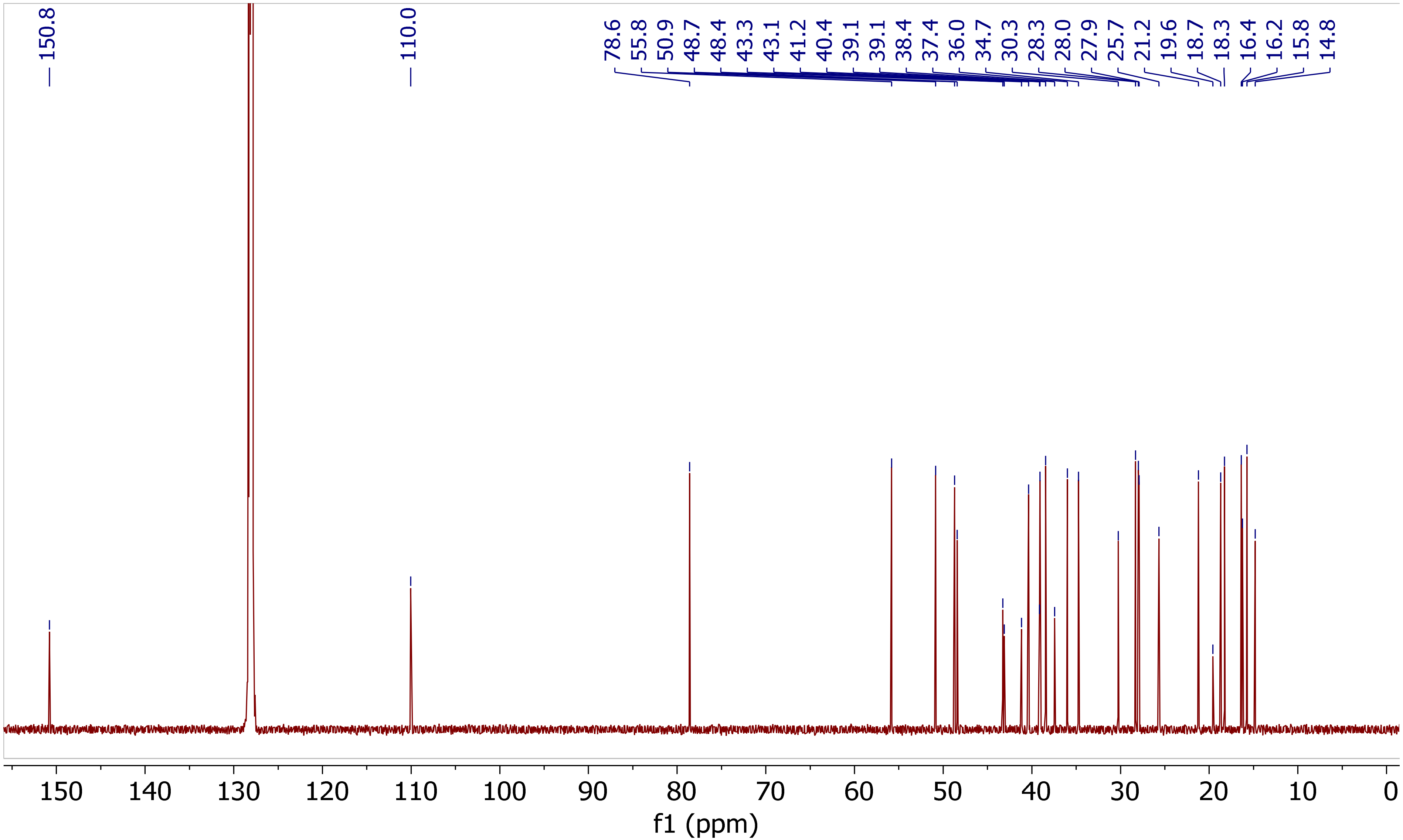

Figure S49. ^{13}C spectrum of lupeol (**8**) (C_6D_6, 151 MHz, 298 K).

Table S1. Phylogenetic group summary based on clades defined in One Thousand Plant Transcriptomes dataset.[2]

Colour code	Abbreviation	Grouped clades	Clades according to One Thousand Plant Transcriptomes
	Outgroups, Algae	Outgroups	Euglenozoa
			Dinophyceae
			Chromista (Algae)
			Glaucophyta (Algae)
		Red Algae	Red Algae
		Green Algae	Green Algae
	Bryophytes, Lycophytes, Ferns	Hornworts, Liverworts, Mosses	Hornworts
			Liverworts
			Mosses
		Lycophytes	Lycophytes
		Ferns	Leptosporangiate Monilophytes
			Eusporangiate Monilophytes
	Gymnosperms	Gymnosperms	Conifers
			Gnetales
			Ginkgoales
			Cycadales
	Basal Angiosperms, Monocots, Magnoliids	Basalmost Angiosperms	Basalmost Angiosperms
			Chloranthales
		Monocots	Monocots
			Monocots/Commelinids
		Magnoliids	Magnoliids
	Eudicots	Basal Eudicots	Basal Eudicots
		Core Eudicots	Core Eudicots
		Rosids	Core Eudicots/Rosids
		Asterids	Core Eudicots/Asterids

Table S2. List of six OSCs tested in this work.

Number	Proposed trivial name	ID	Species	Family	Order	Clade	Product
OSC1	-	scaffold-QDVW-2064089-Saruma_henryi	*Saruma henryi*	Aristolochiaceae	Piperales	Magnoliids	Cycloartenol (**5**) and lanosterol (**6**)
OSC2	-	scaffold-WYIG-2011617-Dombeya_burgessiae	*Dombeya burgessiae*	Malvaceae	Malvales	Core Eudicots/Rosids	None detected
OSC3	-	scaffold-HDWF-2002594-Francoa_appendiculata	*Francoa appendiculata*	Melianthaceae	Geraniales	Core Eudicots/Rosids	None detected
OSC4	(3*S*,13*S*)-Malabarica-17,21-diene-3β,14-diol synthase (MDDS)	scaffold-BLAJ-2025975-Hemerocallis_spp	*Hemerocallis* spp.	Hemerocallidaceae	Asparagales	Monocots	(3*S*,13*S*)-Malabarica-17,21-diene-3β,14-diol (**12**)
OSC5	19-*epi*-Lupeol synthase (19ELS)	scaffold-JVBR-2009111-Aloe_vera	*Aloe vera*	Asphodelaceae	Asparagales	Monocots	19-*epi*-Lupeol (**14**)
OSC6	Protostahopenol synthase (PHS)	scaffold-CLMX-2046562-Escallonia_rubra	*Escallonia rubra*	Escalloniaceae	Asterid II incertae sedis	Core Eudicots/Asterids	Protostahopenol (**15**)

Table S3. NMR shifts of (3*S*,13*S*)-malabarica-17,21-diene-3β,14-diol (**12**) in comparison to literature.[35]

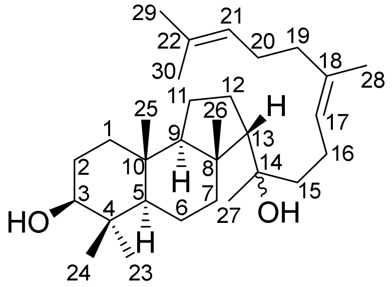

(3*S*,13*S*)-Malabarica-17,21-diene-3β,14-diol (**12**)

No.	**(3*S*,13*S*)-Malabarica-17,21-diene-3β,14-diol (12) (CDCl$_3$, 500 MHz, 298 K)**		**Literature [35] (CDCl$_3$, 400 MHz)**	
	**δ$_C$**	**δ$_H$**	**δ$_C$**	**δ$_H$**
1	39.3	1.55 (m, 1H)<br>1.07 (ddd, *J* = 12.7, 12.7, 5.0 Hz, 1H)	39.2	1.54 (m, 1H)<br>1.05 (m, 1H)
2	27.5	1.62 (m, 1H)<br>1.58 (m, 1H)	27.3	1.65 (m, 1H)<br>1.57 (m, 1H)
3	79.3	3.22 (dd, *J* = 10.7, 5.5 Hz, 1H)	79.2	3.21 (dd, *J* = 11.7, 4.1 Hz, 1H)
4	38.9	-	38.8	-
5	55.7	0.75 (m, 1H)	55.6	0.74 (dd, *J* = 9.1, 2.2 Hz, 1H)
6	19.7	1.63 (m, 1H)<br>1.48 (m, 1H)	19.1	1.63 (m, 1H)<br>1.50 (m, 1H)
7	37.9	1.80 (m, 2H)	37.8	1.80 (m, 2H)
8	44.6	-	44.5	-
9	60.2	1.24 (m, 1H)	60.1	1.25 (m, 1H)
10	37.1	-	36.9	-
11	21.5	1.49 (m, 1H)<br>1.35 (m, 1H)	21.4	1.54 (m, 1H)<br>1.37 (m, 1H)
12	24.4	1.85 (m, 1H)<br>1.53 (m, 1H)	24.3	1.86 (m, 1H)<br>1.53 (m, 1H)
13	58.6	1.69 (m, 1H)	58.5	1.70 (m, 1H)
14	76.2	-	76.1	-
15	41.8	1.54 (m, 1H)	41.7	1.55 (m)
16	22.9	2.05 (m, 2H) [a]	22.8	2.04 (m, 1H)<br>1.55 (m, 1H)
17	124.7	5.13 (tq, *J* = 7.2, 1.2 Hz, 1H)	124.6	5.13 (t, *J* = 6.1 Hz, 1H)
18	135.4	-	135.3	-
19	39.9	2.06 (m, 1H)<br>1.99 (m, 1H)	39.8	2.06 (m, 1H)<br>1.98 (m, 1H)
20	26.8	2.07 (m, 2H) [a]	26.7	2.09 (m, 1H)<br>1.99 (m, 1H)
21	124.4	5.08 (t sept, 7.0, 1.4 Hz, 1H)	124.3	5.08 (t, *J* = 7.1 Hz, 1H)
22	131.6	-	131.5	-
23	28.2	0.98 (s, 3H)	28.1	0.98 (s, 3H)
24	15.4	0.78 (s, 3H)	15.3	0.78 (s, 3H)
25	16.5	0.84 (s, 3H)	16.4	0.84 (s, 3H)
26	26.7	0.95 (s, 3H)	26.5	0.94 (s, 3H)
27	25.9	1.21 (s, 3H)	25.8	1.21 (s, 3H)
28	16.2	1.62 (s, 3H)	16.1	1.62 (s, 3H)
29	25.8	1.68 (s, 3H)	25.8	1.68 (s, 3H)
30	17.9	1.60 (s, 3H)	17.7	1.60 (s, 3H)

[a] Only a single signal for both diastereotopic protons was identified. Our HSQC and HMBC spectra did not provide any support for two separated proton signals. Our assignment is in agreement with a dataset of **12** measured in C_6D_6.[36]

Table S4. NMR shifts of 19-*epi*-lupeol (**14**) in comparison to lupeol (**8**) (own measurements) and lupeol acetate (**34**) literature data (all in C_6D_6).[37]

	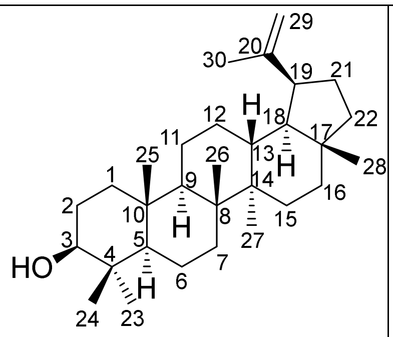 19-*epi*-Lupeol (**14**)		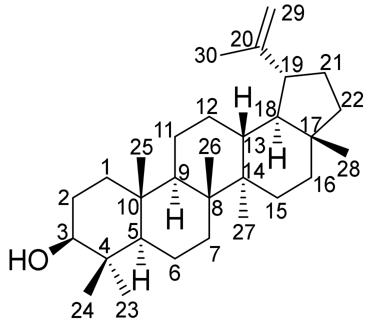 Lupeol (**8**)		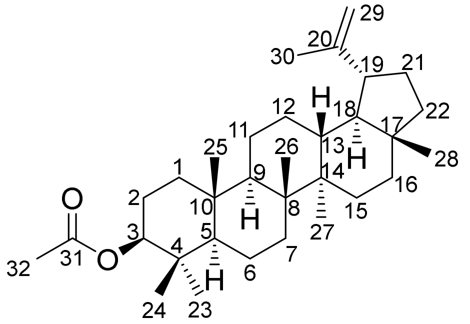 Lupeol acetate (**34**)		
No.	**19-*epi*-Lupeol (14) (C_6D_6, 500 MHz, 298 K)**		**Lupeol (8) (C_6D_6, 600 MHz, 298 K)**		**Lupeol acetate literature [37] (C_6D_6, 600 MHz)**		
	**δ_C**	**δ_H**	**δ_C**	**δ_H**	**δ_C**	**δ_H (referenced to 7.28 ppm)**	**δ_H (adjusted to 7.16 ppm) [a]**
1	39.2	1.53 (m, 1H)<br>0.77 (m, 1H)	39.1	1.53 (m, 1H)<br>0.77 (m, 1H)	38.54	1.59 (m, 1H)<br>0.88 (m, 1H)	1.47 (m, 1H)<br>0.76 (m, 1H)
2	28.0	1.43 (m, 2H)	28.0	1.43 (m, 2H)	24.11	1.84 (m, 1H)<br>1.72 (m, 1H)	1.72 (m, 1H)<br>1.60 (m, 1H)
3	78.6	3.05 (dd, *J* = 10.5, 5.7 Hz, 1H)	78.6	3.02 (dt, *J* = 11.0, 5.5 Hz, 1H)	80.56	4.82 (dd, *J* = 11.8, 4.5 Hz, 1H)	4.70 (dd, *J* = 11.8, 4.5 Hz, 1H)
4	39.1	-	39.1	-	38.00	-	-
5	55.9	0.64 (m, 1H)	55.8	0.63 (bd, *J* = 11.6 Hz, 1H)	55.65	0.806 (bd, *J* = 11.2 Hz, 1H)	0.69 (bd, *J* = 11.2 Hz, 1H)
6	18.8	1.50 (m, 1H)<br>1.37 (m, 1H)	18.7	1.49 (m, 1H)<br>1.35 (m, 1H)	18.52	1.52 (m, 1H)<br>1.42 (m, 1H)	1.40 (m, 1H)<br>1.30 (m, 1H)
7	34.6	1.37 (m, 2H)	34.7	1.36 (m, 2H)	34.58	1.44 (m, 2H)	1.32 (m, 2H)
8	41.3	-	41.2	-	41.11	-	-
9	51.1	1.35 (m, 1H)	50.9	1.23 (m, 1H)	50.58	1.31 (m, 1H)	1.19 (m, 1H)
10	37.4	-	37.4	-	37.25	-	-
11	21.3	1.46 (m, 1H)<br>1.30 (m, 1H)	21.2	1.36 (m, 1H)<br>1.16 (m, 1H)	21.13	1.40 (m, 1H)<br>1.21 (m, 1H)	1.28 (m, 1H)<br>1.09 (m, 1H)
12	25.6	1.90 (m, 1H)<br>1.15 (m, 1H)	25.7	1.82 (m, 1H)<br>1.18 (m, 1H)	25.57	1.93 (bd, *J* = 12.3 Hz, 1H)<br>1.25 (m, 1H)	1.81 (bd, *J* = 12.3 Hz, 1H)<br>1.13 (m, 1H)
13	35.2	2.16 (td, *J* = 12.5, 3.4 Hz, 1H)	38.4	1.67 (m, 1H)	38.40	1.77 (m, 1H)	1.65 (m, 1H)
14	43.3	-	43.1	-	43.10	-	-
15	28.0	1.71 (m, 1H)<br>0.98 (m, 1H)	27.9	1.69 (m, 1H)<br>1.00 (m, 1H)	27.87	1.80 (m, 1H)<br>1.12 (m, 1H)	1.68 (m, 1H)<br>1.00 (m, 1H)
16	37.3	1.53 (m, 1H)<br>1.34 (m, 1H)	36.0	1.52 (m, 1H)<br>1.41 (m, 1H)	35.95	1.64 (m, 1H)<br>1.53 (m, 1H)	1.52 (m, 1H)<br>1.41 (m, 1H)
17	42.2	-	43.3	-	43.24	-	-
18	50.9	1.42 (m, 1H)	48.7	1.41 (m, 1H)	48.69	1.54 (m, 1H)	1.42 (m, 1H)
19	45.1	2.47 (td, *J* = 9.8, 5.9 Hz, 1H)	48.4	2.44 (td, *J* = 11.1, 5.8 Hz, 1H)	48.41	2.56 (m, 1H)	2.44 (m, 1H)
20	150.6	-	150.8	-	150.7	-	-
21	31.0	2.00 (m, 1H)<br>1.52 (m, 1H)	30.3	1.99 (m, 1H)<br>1.44 (m, 1H)	30.23	2.10 (m, 1H)<br>1.55 (m, 1H)	1.98 (m, 1H)<br>1.43 (m, 1H)
22	41.8	1.55 (m, 1H)<br>1.14 (m, 1H)	40.4	1.42 (m, 1H)<br>1.24 (m, 1H)	40.34	1.55 (m, 1H)<br>1.36 (m, 1H)	1.43 (m, 1H)<br>1.24 (m, 1H)
23	28.3	1.03 (s, 3H)	28.3	1.03 (s, 3H)	28.11	1.023 (s, 3H)	0.90 (s, 3H)
24	15.7	0.81 (s, 3H)	15.7	0.81 (s, 3H)	16.83	1.033 (s, 3H)	0.91 (s, 3H)
25	16.3	0.79 (s, 3H)	16.4	0.78 (s, 3H)	16.34	0.887 (s, 3H)	0.77 (s, 3H)
26	16.2	1.05 (s, 3H)	16.2	1.00 (s, 3H)	16.18	1.099 (s, 3H)	0.98 (s, 3H)
27	14.7	0.93 (s, 3H)	14.8	0.94 (s, 3H)	14.83	1.078 (s, 3H)	0.96 (s, 3H)
28	20.9	1.00 (s, 3H)	18.3	0.82 (s, 3H)	18.23	0.943 (s, 3H)	0.82 (s, 3H)
29	109.5	4.93 (br s, 1H)<br>4.80 (m, 1H)	110.0	4.88 (d, *J* = 2.0 Hz, 1H)<br>4.75 (br s, 1H)	110.0	5.00 (s, 1H)<br>4.88 (s, 1H)	4.88 (s, 1H)<br>4.76 (s, 1H)
30	25.4	1.69 (s, 3H)	19.6	1.71 (s, 3H)	19.52	1.845 (s, 3H)	1.73 (s, 3H)
31	-	-	-	-	169.9	-	-
32	-	-	-	-	20.84	1.877 (s, 3H)	1.76 (s, 3H)

[a] For better comparison, the reported ^{1}H shifts referenced to 7.28 ppm were adjusted to the more common reference value 7.16 ppm[38] by subtracting 0.12 ppm.

Table S5. ^{13}C NMR shifts of 19-*epi*-lupeol (**14**) in comparison to literature data (all in $CDCl_3$).[39]

Substantial differences to literature data are highlighted in red.

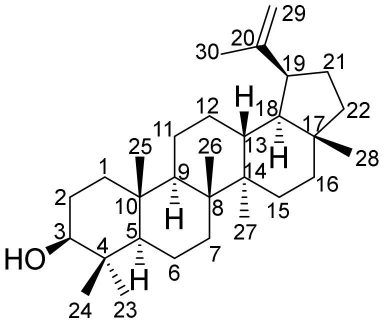

19-*epi*-Lupeol (**14**)

No.	19-*epi*-Lupeol (14) ($CDCl_3$, 126 MHz, 298 K) δ_C	Literature [39] ($CDCl_3$, 22.5 MHz) δ_C
1	38.9	38.83
2	27.6	27.50
3	79.2	79.04
4	39.0	38.93
5	55.5	55.46
6	18.5	18.44
7	34.3	34.19
8	41.1	41.04
9	50.8	50.71
10	37.4	37.30
11	21.0	20.93
12	25.4	25.26
13	35.0	34.91
14	43.2 [a]	41.88
15	27.7	27.63
16	37.0	36.95
17	42.0	42.11
18	50.8	50.71
19	44.9	44.86
20	150.7	150.51
21	30.8	30.68
22	41.5	41.45
23	28.1	28.05
24	15.5	15.38
25	16.3	16.14
26	16.1	16.05
27	14.5	14.45
28	20.7	20.62
29	109.1	108.97
30	25.3	25.10

[a] Our assignment is supported by an HMBC correlation from H26 at 1.05 ppm to C14 at 43.2 ppm.

Table S6. NMR shifts of protostahopenol (**15**) in comparison to the stereoisomer hopenol B (**16**).[10]

	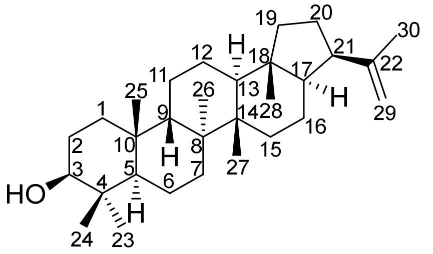 Protostahopenol (**15**)		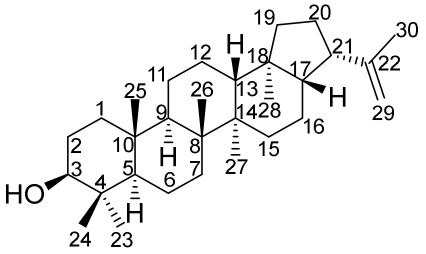 Hopenol B (**16**)	
**No.**	**Protostahopenol (15)**<br>**($CDCl_3$, 600 MHz, 298 K)**		**Hopenol B (16)**[10]<br>**($CDCl_3$, frequency/temperature not provided)**	
	δ_C	δ_H	δ_C	δ_H
1	33.3	1.39 (m, 2H)	39.0	1.70 (m, 1H)<br>0.93 (m, 1H)
2	29.3	1.69 (m, 1H)<br>1.58 (m, 1H)	21.2	1.51 (m, 1H)<br>1.33 (m, 1H)
3	79.4	3.22 (dd, *J* = 11.7, 4.9 Hz, 1H)	79.2	3.19 (dd, *J* = 11.8, 4.2 Hz, 1H)
4	39.3	-	38.9	-
5	48.1	1.34 (m, 1H)	55.0	1.39 (m, 1H)
6	19.2	1.52 (m, 1H)<br>1.14 (m, 1H)	18.5	1.52 (m, 1H)<br>1.39 (m, 1H)
7	34.9	1.95 (m, 1H)<br>1.10 (m, 1H)	33.4	1.46 (m, 1H)<br>1.24 (m, 1H)
8	41.7	-	42.1	-
9	46.1	1.38 (m, 1H)	50.5	1.22 (m, 1H)
10	37.1	-	37.3	-
11	22.6	1.50 (m, 1H)<br>1.42 (m, 1H)	21.8	1.64 (m, 1H)<br>1.47 (m, 1H)
12	24.5	1.41 (m, 2H)	27.5	1.79 (m, 1H)<br>1.61 (m, 1H)
13	49.9	1.34 (m, 1H)	49.6	1.36 (m, 1H)
14	43.2	-	41.8	-
15	33.8	1.30 (m, 2H)	33.8	1.39 (m, 1H)<br>1.24 (m, 1H)
16	21.6	1.61 (m, 1H)<br>1.48 (m, 1H)	24.1	1.47 (m, 1H)<br>1.41 (m, 1H)
17	55.3	1.38 (m, 1H)	55.2	0.68 (m, 1H)
18	45.0	-	44.9	-
19	41.8	1.59 (m, 1H)<br>1.03 (m, 1H)	42.2	1.61 (m, 1H)<br>1.02 (m, 1H)
20	27.5	1.83 (m, 2H)	27.5	1.86 (m, 1H)<br>1.56 (m, 1H)
21	46.5	2.67 (ddd, *J* = 9.2, 9.2, 6.6 Hz, 1H)	46.6	2.68 (q, *J* = 9.1 Hz, 1H)
22	148.9	-	148.9	-
23	29.2	0.97 (s, 3H)	28.2	0.97 (s, 3H)
24	16.2	0.78 (s, 3H)	16.0	0.76 (s, 3H)
25	22.8	0.89 (s, 3H)	15.5	0.82 (s, 3H)
26	22.8	1.12 (s, 3H)	16.9	0.96 (s, 3H)
27	17.3	0.87 (s, 3H)	16.8	0.93 (s, 3H)
28	16.1	0.70 (s, 3H)	16.2	0.72 (s, 3H)
29	110.2	4.78 (s, 2H)	110.2	4.78 (br s, 2H)
30	25.2	1.75 (s, 3H)	25.2	1.75 (s, 3H)

Table S7. NMR shifts of dammarenediol II (**18**), product of 19ELS mutants C119G and G409S, in comparison to literature.[40]

Inconsistent assignments in the literature are highlighted in red.

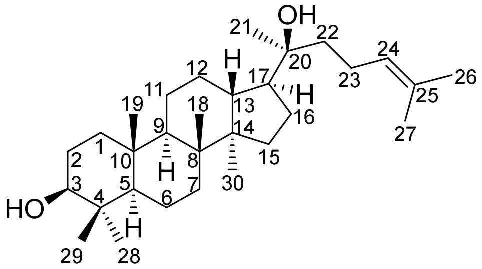

Dammarenediol II (**18**)

No.	Dammarenediol II (18) (CDCl3, 600 MHz, 298 K)		Literature [40] (CDCl3, 50 MHz)	Literature [41] (CDCl3, 125 MHz)
	δ_C	δ_H	δ_C	δ_C
1	39.2	1.69 (m, 1H)<br>0.97 (m, 1H)	39.0	39.0
2	27.5	1.61 (m, 2H)	27.4	27.4
3	79.1	3.20 (dd, *J* = 11.5, 4.2 Hz, 1H)	78.9	79.0
4	39.1	-	39.1	39.0
5	56.0	0.73 (m, 1H)	55.9	55.8
6	18.4	1.53 (m, 1H)<br>1.44 (m, 1H)	18.3	18.3
7	35.4	1.52 (m, 1H)<br>1.28 (m, 1H)	35.2	35.2
8	40.5	-	40.4	40.3
9	50.8	1.32 (m, 1H)	50.6	50.6
10	37.3	-	37.1	37.1
11	21.7	1.50 (m, 1H)<br>1.31 (m, 1H)	21.5	21.5
12	25.0	1.71 (m, 1H)<br>1.47 (m, 1H)	25.4	27.5
13	42.4	1.62 (m, 1H)	42.3	42.3
14	50.4	-	50.3	50.3
15	31.3	1.45 (m, 1H)<br>1.06 (m, 1H)	31.2	31.2
16	27.7	1.81 (m, 1H)<br>1.24 (m, 1H)	27.6	24.8
17	50.0	1.74 (m, 1H)	49.9	49.8
18	15.6	0.95 (s, 3H)	16.2	15.5
19	16.4	0.84 (s, 3H)	15.5	16.2
20	75.6	-	75.4	75.4
21	25.5	1.14 (s, 3H)	24.8	25.4
22	40.6	1.47 (m, 2H)	40.5	40.5
23	22.7	2.04 (m, 2H)	22.6	22.5
24	124.9	5.11 (t, *J* = 5.9 Hz, 1H)	124.7	124.7
25	131.8	-	131.6	131.6
26	25.9	1.69 (s, 3H)	25.7	25.7
27	17.9	1.62 (s, 3H)	17.7	17.7
28	28.1	0.97 (s, 3H)	28.0	28.0
29	15.5	0.77 (s, 3H)	15.4	15.3
30	16.6	0.87 (s, 3H)	16.5	16.4

Table S8. NMR shifts of camelliol C (**19**), product of 19ELS mutant S366G, in comparison to literature.[42]

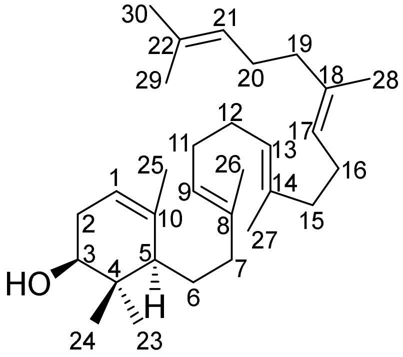

Camelliol C (**19**)

No.	Camelliol C (19) (CDCl$_3$, 400 MHz, 298 K)		Literature[42] (CDCl$_3$, 500 MHz)	
	δ$_C$	δ$_H$	δ$_C$	δ$_H$
1	118.4	5.24 (m, 1H)	118.3	5.24 (m, 1H)
2	31.9	2.22 (m, 1H)<br>1.99 (m, 1H)	31.8	2.24 (m, 1H)<br>1.97 (m, 1H)
3	75.2	3.46 (dd, *J* = 7.7, 6.1 Hz, 1H)	75.1	3.46 (br t, *J* = 6.9 Hz, 1H)
4	38.2	-	38.1	-
5	49.0	1.63 (m, 1H)	49.0	1.65 (m, 1H)
6	27.3	1.73 (m, 1H)<br>1.33 (m, 1H)	27.2	1.76 (m, 1H)<br>1.35 (m, 1H)
7	42.2	2.17 (m, 1H)<br>1.97 (m, 1H)	42.0	2.16 (m, 1H)<br>1.96 (m, 1H)
8	135.3	-	135.2	-
9	124.8	5.15 (m, 1H)	124.7 [b]	5.15 (m, 1H)
10	137.2	-	137.1	-
11	28.4	2.02 (m, 2H)	28.3	2.02 (m, 2H)
12	28.4	2.02 (m, 2H)	28.3	2.02 (m, 2H)
13	124.4	5.15 (m, 1H)	124.3 [b]	5.15 (m, 1H)
14	135.5 [a]	-	134.9 [c]	-
15	39.9	1.97 (m, 2H)	39.8	1.98 (m, 2H)
16	26.8	2.07 (m, 2H)	26.7	2.07 (m, 2H)
17	124.4	5.11 (m, 1H)	124.2 [b]	5.12 (m, 1H)
18	135.1 [a]	-	135.4 [c]	-
19	39.9	1.97 (m, 2H)	39.8	1.98 (m, 2H)
20	26.9	2.07 (m, 2H)	26.8	2.07 (m, 2H)
21	124.5	5.10 (m, 1H)	124.4	5.10 (m, 1H)
22	131.4	-	131.3	-
23	25.5	0.97 (s, 3H)	25.4	0.97 (s, 3H)
24	16.3	0.83 (s, 3H)	16.2	0.83 (s, 3H)
25	22.7	1.71 (br s, 3H)	22.6	1.72 (br s, 3H)
26	16.2	1.62 (s, 3H)	16.0	1.60 (s, 3H)
27	16.2	1.60 (s, 3H)	16.1	1.60 (s, 3H)
28	16.1	1.60 (s, 3H)	16.1	1.60 (s, 3H)
29	17.8	1.60 (s, 3H)	17.7	1.60 (s, 3H)
30	25.9	1.68 (s, 3H)	25.7	1.68 (s, 3H)

[a] Interchangeable.

[b,c] Reported as interchangeable.

Table S9. NMR shifts of (20*R*)-protosta-13(17),24-dien-3β-ol (**21**), product of PHS mutants F728A and F728S, in comparison to literature.[43]

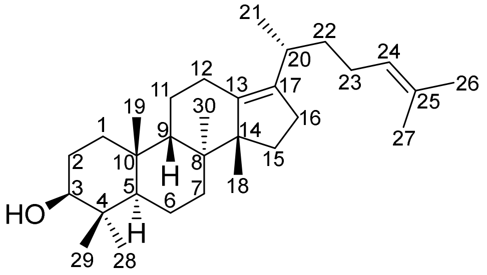

(20*R*)-Protosta-13(17),24-dien-3β-ol (**21**)

No.	(20*R*)-Protosta-13(17),24-dien-3β-ol (21) (CDCl$_3$, 600 MHz, 298 K)		Literature [43] (CDCl$_3$, 800 MHz)	
	δ$_C$	δ$_H$	δ$_C$	δ$_H$
1	34.0	1.42 (m, 2H)	33.8	1.52 (m, 1H)<br>1.41 (m, 1H)
2	29.0	1.68 (m, 1H)<br>1.60 (m, 1H)	28.9	1.69 (m, 1H)<br>1.62 (m, 1H)
3	79.4	3.21 (dd, *J* = 11.6, 4.9 Hz, 1H)	79.2	3.22 (dt, *J* = 11.7, ~5 Hz, 1H)
4	39.5	-	39.3	-
5	47.8	1.39 (m, 1H)	47.6	1.41 (m, 1H)
6	18.5	1.55 (m, 1H)<br>1.26 (m, 1H)	18.4	1.53 (m, 1H)<br>1.25 (m, 1H)
7	33.5	1.89 (m, 1H)<br>1.25 (m, 1H)	33.3	1.86 (m, 1H)<br>1.25 (m, 1H)
8	40.3	-	40.1	-
9	47.4	1.63 (m, 1H)	47.2	1.64 (m, 1H)
10	37.0	-	36.8	-
11	23.2	1.50 (m, 1H)<br>1.20 (m, 1H)	23.0	1.53 (m, 1H)<br>1.25 (m, 1H)
12	23.4	2.36 (ddd, *J* = 14.3, 5.3, 1.5 Hz, 1H)<br>1.81 (m, 1H)	23.2	2.37 (ddd, *J* = 14.2, 5.0, 2.1 Hz)<br>1.83 (m, 1H)
13	139.2	-	139.0	-
14	57.6	-	57.4	-
15	31.1	1.83 (m, 1H)<br>1.28 (m, 1H)	30.9	1.83 (m, 1 H)<br>1.25 (m, 1H)
16	28.8	2.13 (m, 2H)	28.6	2.17 (m, 1H)<br>2.10 (m, 1H)
17	135.4	-	135.2	-
18	22.8	1.06 (s, 3H)	22.6	1.06 (s, 3H)
19	24.1	0.98 (s, 3H)	23.9	0.96 (s, 3H)
20	31.7	2.46 (dqd, *J* = 6.8, 6.8, 6.8 Hz, 1H)	31.6	2.46 (dqd, *J* = 8.8, 6.9, 6.1 Hz, 1H)
21	20.2	0.95 (d, *J* = 7.0 Hz, 3H)	20.1	0.95 (d, *J* = 6.9 Hz, 3H)
22	35.8	1.28 (m, 2H)	35.6	1.25 (m, 2H)
23	26.5	1.81 (m, 2H)	26.3	1.83 (m, 2H)
24	125.1	5.07 (t, *J* = 7.3 Hz, 1H)	125.0	5.07 (m, 1H)
25	131.1	-	130.9	-
26	25.9	1.67 (s, 3H)	25.7	1.67 (q, *J* = 1.2 Hz, 3H)
27	17.8	1.56 (s, 3H)	17.6	1.56 (br d, *J* = ~1, 3H)
28	29.1	0.98 (s, 3H)	28.9	0.99 (s, 3H)
29	16.1	0.80 (s, 3H)	16.0	0.80 (s, 3H)
30	23.1	0.94 (s, 3H)	22.9	0.94 (s, 3H)

Table S10. Crystallographic details of protostahopenol (**15**).

2(**15**)·(CH_4O)	
CCDC code	2379089
Empirical formula	$C_{61}H_{104}O_3$
Formula weight	885.44
Crystal system	Monoclinic
Space group	*C*2
a (Å)	13.52280(10)
B (Å)	6.43420(10)
C (Å)	59.7120(6)
α (°)	90
β (°)	90.6440(10)
γ (°)	90
Volume ($Å^3$)	5195.12(10)
Z	4
Densitiy (g/cm^3)	1.132
F(000)	1976
Radiation	Cu Kα (λ = 1.54184)
Temperature (K)	100.00(10)
μ (mm^{-1})	0.498
Crystal size (mm)	0.62×0.14×0.11
$2\theta_{max}$	159.318
Measured reflections	112480
Independent reflections	10753
Obsvd. [$I > 2\sigma(I)$]	10427
R indices [all data]	R_1 = 0.0564 wR_2 = 0.1478
Largest diff. peak/hole e·$Å^3$	0.294 / -0.285
S	1.082
Flack parameter	0.05(11)

Table S11. Coding sequences of tested OSCs.

**Gene and coding sequence**
**> OSC1 (scaffold-QDVW-2064089-Saruma_henryi)**<br>ATGTGGAAGCTAAAGATCGCAGAGGGTGAAGATCCATGGCTTAGAAGCACAAATAATTTCTTCGGGAGACAAATATGGGAGTTCCACC<br>CACATCTTGGAAGCCATGAAGAGAGGGTGGAGATTGAGATGGCTCGTGATGAATTTTGGAAAAATCGGTTTCAGATGAAGCACAGTTC<br>AGATCTATTAATGCGAATGCAGTTTGCAAAGGAGAACCAATGTGATATAAAGCTTCCCCAAGTGATGCTAAAAGAAAATGAGGAAATTA<br>CAGAGGATAAAGTAACAATAACTTTAAGAAGAGCCATCAACTTCTACTCCATCATCCAGGCCCATGATGGGCATTGGCCCAGCGACTA<br>TGGTGGTTCATCCTTTCTTCTACCTGGTTTAGTGATTAGTTTATATGTTACAGGAGCTCTCAACACAATCTTATCTGCAGAGCATAGAAA<br>AGAGATACGCCGTTACATTTATAACCATCAGAACGCAGATGGTGGATGGGGTCTGCATATAGAGGGTCATAGCATCATGTTTTCTTCA<br>GTCCTTTTCTATGTTGTTCTGAGATTGCTTGGAGAAGATGGAGGTGATGCAAATGGAGATGTTGCAACGGAGAAAGCAAGGAGATGGG<br>TGTTGGATCGTGGTGGGGCGGCTTATATACCATCATGGGGAAAATTGTGGCTTTCGGTTCTTGGTGTATATGACTGGTCGGGAAATAA<br>TCCAATGCCGCCTGAGATGTGGCTTCTTCCTTACAGTCTTCCTTTCCATCCAGGACGGACATGGTGTCACTGTCGGTTGGTTTATCTG<br>CCCATGTCATATTTATATGGGAAGAAGTTTGTTGGCCCAATCACTGAAACAGTTTTGTCACTAAGAAACGAACTCTACACTTGCCCATA<br>CAGTAAAGTAGATTGGAATCAAGCGCGTAACTTGTGTGCGAAGGAAGATCTCTACCATAAACATCCCCTGCTACAAGATATCCTTTGG<br>GGATGTCTGTATTATATTGGGGAACCTATTCTCAAGTTCTGGCCTTTCTGCAAGCTGAGAGAAAAGGCTCTGAGCACTGTAATGCAGC<br>ATATGCACTACGAAAATGAGAATACTCGGTACCTGTGCATAGGTCCTGTGAACAAAGTGTTAAACATGCTTTGTAGTTGGGTAGAAAAT<br>CCAAATTCAGAGGCATTCAAGTTGCACCTCCCGAGACTATACGATTATTTATGGGTTGCTGAAGATGGAATGAAGATGAAGGGAAGTG<br>ATGGCAGTCAGCTTTGGGACACAGCTCTTGCTGTTCAAGCAATTGTCTCATGTAACCTTGTGGATCAATACAGTGTGATGCTTAAAAAG<br>GCTCATAAGTACATAAAGATGTCGCAGGTTTTGGAAAATTGTGCTGGTGATTTTAGGCTATGGTACCGCCACATTTCTAAAGGTGCATG<br>GACATTCACAACAGCAGATAATGGGTGGCAAGTATCAGATTGCACAGGAGAAGGACTCAAGGCTGCACTATTATTGTCAAAGCTCCCA<br>CGAGAAATCGTTGGAGAACCACTAGCTGAAGACCGATTCTATGATTCCGTGAATGTTCTTCTTTCTCTACAGAACTCCAATGGAGGGTA<br>TGCTACATACGAGCTCACAAGATCTTATAGATGGATGGAGGTGCTAAATGCTTCTGAACTTTTTGGAGATATTATCATTGATTATCCGTA<br>TGTTGAGTGCACATCGTCAGCAATTCAAGCTTTGATATCATTTATGAAGTTGTATCCAGCGCATCGAAGGAAAGAAATACAATCTTGCA<br>TCGCAAAGGGAGTTAGTTTCATTGAAAATGCGCAACTGTGTGACGGTTCATGGTATGGCTGGTGGGGAATTTGCTACACCTACGGCAC<br>ATGGTTTGGAGTAGCAGGGTTGGTGGCTGCTGGAAAAGACTACCAGAATTGCTCTGGCATCCAAAAAGCTTGTGATTTCCTGTTATCG<br>AAACAGCTCGCTTGTGGTGGGTGGGGAGAGAGTTACCTTTCCTGTGAAAACAAGGTGTACACTAATCTTGAAGGGGACAGGTCTCAT<br>CTAGTAAATACTGCATGGGCTATGCTGGCTCTTATTGAAGCCGGGCAGGCCAAGAGAGACCCACTGCCGTTGCATCGAGCAGCGAGG<br>GTGCTAATCAATTCACAAATGGGAAATGGTGACTTCCCACAACAGGAAATTATTGGAGTTTTCAATCAGAATTGTATGATCAGTTATTCC<br>TCGTACAGAAACATTTTTCCTATATGGGCTCTAGGGGAATATCGCAGGGTATTGTGA
**> OSC2 (scaffold-WYIG-2011617-Dombeya_burgessiae)**<br>ATGTGGAGGCTTAAGATAGGAAAGGGTACTGCTAACGATCCGTACCTATTCAGTACCAACAATTTCTTGGGAAGGCAGACATGGGAGT<br>TTGATCCTAACGCAGGCACTCCTGAAGAGCGAGCGAAGGTTGAAGAAGCTCGTCAAAATTACTATAAAAATCGTTTCAATGTCAAGCC<br>TAGTTCCGACCTCCTTTGGCAAATGCAGTCTCTAAAAGAGAAAAACTTCAAAACAATTCCCCAAGTAAAGATTAAGGATGGAGAGGAAA<br>TTACATATACAGCAGCTACAGCCGCATTAAAGAGAGCTGTTCACTTCTTGTCAGCCATGCAATCAAGTGATGGCCACTGGCCTGCCGA<br>GATTACTGGTCCAACGTTCTATTTTCCTATGATGGCAATTCCTTTGTACATCACTGGGCATCTTTACGTTATATTCTCACCAGATCATCG<br>GAAAGAAATTCTTCGTTACATGTACAATCATCAGAACGAAGATGGTGGATGGGGATTACATATAGAGAGTCCAAGCATAATGTTCAGCA<br>CTGCTCTGAACTACATTTGCTTGCGTATGCTTGGGGAAGGGCCTGATGGCGGCCAAGACAATGCTTGTGAGAGAGCAAGAAAATGGA<br>TTCTCGAACGTGGTGGTGTAACAACCATACCATCTTGGGGGAAAATATGGCTATCTGTACTTGGTCTATATGATTGGTCGGGCTGCCA<br>TCCCATGCCCCCAGAGTTTTGGCTCCTTCCTTCTTATTTTCCTATTAGTGCAGCCAAACTGTGGTACTATTGTCGGGTGACTTACTATG<br>CTATGTCGTATTTTTATGGAAAAAAATTTGTTGGTACAATCACACCTCTCGTTCAACAGTTGAGAGAAGAACTCCATATTGAACCTTATC<br>ATAAAATTAATTGGAAGCAAAAGCGCCATTTATGTGCAAAGGAGGATCTTCATTATCCCCATACTTTTCTACAAACGTTACTATGGGATA<br>GTCTTTACTCATTTTCAGAGCCTCTATTTTCTCGATGGCCTTTTAAGAAGTTAAGAGAAAAGGCTCTCCAAATAACAATGAATCACGTTC<br>ATTATGAAGATGAATGTAGTCGGTACATTCAGATAGCAATTGCGGAGAAGTCCGTAACCATGCTTGCTTGTTGGATAGAAGATCCTAAC<br>GGGGATTGTTTTAAAAAGCACCTTGCTAGGATTGCTGACTACATTTGGATTGCAGAAGATGGAATGAAGTTACAGAGCGTCGGAAGTC<br>AAACTTGGGAATCTAGTTTGGCTCTTCAAGCTTTGCTTGCTAGCAATCTTACTGCCGAAATTGGACCTACATTGAAGAAAGGACACTAT<br>TTCCTGAAAAATTCTCAGGCCAAAGATAATCCTCCTGGTGACTTTAGAAGAATGTTCAAACACATGACTAAAGGATCATGGACTTTTTCC<br>GATCTTGATCATGGATGTCAAGTTTCGGATTGCACAGCAGAAAGTTTGAAGTTATGCCTATATTTCTCCAAGATGCAACCAGAAATCGT<br>TGGTCAGAAAATGGAACCAGAGAGATTCTACGATGCTGTCAATTTTATATTATCCTTACAGAGTGAAAATGGGGGCTTCACAGGATGG<br>GAGCCAAAAACAGCTGGATCATGGTTGGAGTGGTTCAACCCTGTGGAGTTTCTAGAAAACATTATCGTTGAGCATGACCATGTGGAGT<br>GCACTTCATCAGCAATGCAGGCACTAATTCTGTTTCAGAAGTATTACCCTGAGCATAGAACAAAAGAAATTGACAAGTGCATTGCAAAA<br>GCTGTCCAGTTTCTTGAAGACACGCAAAAGCCTAATGGCTCATGGTATGCAAGTTGGGGGGTTTGCTTCATTTATGCAACATATTTTGC<br>ACTCGTGGGTTTAGCTGCTGCAGGTAAGACTTACAACAATTGTTTGGCTGTGCGTAAAGGAGTTAGTTTTCTGCTTGAAACCCAAACAG<br>ATGATGGGGGCTGGGGGGAGAGTTATCTTTCCATTCCAAACAAGGTATATACACCTCTTCCAGGCAAGAGATCAAATTTGGTGCATAC<br>TGCAATGGCCTTGATGGGTTTAATTCATGGAGGACAGGCAAAAAGAGATCCTAATCCCCTTCATCGTGCTGCAAAGCTTTTGATCAATT<br>CCCAATTACCAGATGGTGATTTTCCCCAGCAGGAAATGACGGGAGCTTTCTTGGGGAATGGCATACTGCACTACGCGTATAGGAAAAC<br>TTACCCAACCTGGGCTTTGGCCGAATATTGCAAGCATGTTGCACTGCCTTGA
**> OSC3 (scaffold-HDWF-2002594-Francoa_appendiculata)**<br>ATGTGGAGGCTTAAGATAGGAGAAGACGGTAACAAAGACCCATATTTGTCTAGCACAAACAACTACACGGGCAGGCAGACTTGGGAA<br>TTTGACCCCGATGCAGGTACACCTGAAGAACGCCAAGAGATTGAAAGTGCTCGTATTAATTACTACAAAAATCGTTACAATGTTAGACC<br>GAGCTCTGACCTCTTTTGGAGAATGCAGTTTCTAAGAGAGAAAAATTTCAAACAAACAATTCCACGAGTAGAAGTTGAAGATGGTGAGG<br>ATATTACGTATGAAACTGCCACAGCTGCATTGAGGAGGTCAATCCATTACCTTTGTGCTTTGCAAAGTAGCCATGGTCATTGGCCTGCT<br>GAAATTTCTGGAGATATGTTTTTCCATCCTCCCTTGGTCATGTGTTTATATATCACAGGGCATCTGAATACTGTGCTACATGCAGAACAC<br>CAAAAAGAAATTCTTCGTTACATATATTGTCATCAGAATGAAGACGGTGGCTGGGGATTACACATAGAAAGTCCTAGTACCATGTTTGG<br>TACTGTTTTGAACTACATCTGTATGCGTCTACTCGGAGAAGGACCAGATGGTGGCCAAGATGATGCTTGTGCAAGAGGCCGAAAGTG

GATTCTTGATCATGGCGGTGCAACATCCATACCATCATGGGGAAAGACATGGCTTGCTATACTTGGTGTGTATGAATGGTCAGGAAGC
AACCCAATGCCTCCTGAGTTGTGGATCTTTCCTTATATTTTCCCTTTGCATCCAGCAAAGATGTTCTGCTACTGCCGGCTAACTTATATG
CCAATGTCTTATTTGTATGGGAAGAGGTTTGTCGGGCCAATCACACCTCTCATTTTAGAATTGAGACAAGAAATATACAGTGAGCCTTA
TGATAAAATAAAATGGAGTAGCATGCGCCATTTATGTGCAAAGGAGGATAACCATTATCCTCAACCTTGGATACAAAAAATGCTATGGA
ATAGTCTCTATATGTTTTCCGAACCTCTTCTGAATCGATGGCCCTTTAACAAATTGAGAGGAAAGGTTCTTCAAGAAACAATGAAACACA
TTCATTACGAGGATGAAAGCAGTCGATACATTACCATTGGGATCGTGGAAAAGCCACTACATATGCTTAGTTGCTGGGTGGAAGATCC
AAATGGAGAATACTTTATGAAACACATTCCTCGAATCCAAGATTACCTTTGGATGGGAGAAGATGGGATGAAAATGCAGGGTTTTGGTA
GTCAGGTTTGGGATACTGCTCTTGCCTTGCAGGCTTTGCTTGCTACCAATTATTATGATGAGTATTATACGATACTCAAGAAGGGGCAC
GACTTTTTGAAGAAATCACAGGTGAGAAATAATCCTGCTGGTGACTATAAAAAGATGTATCGTCACACTTCAAAAGGAGCATGGACTTT
GTCTGATCAGGATCATGGATGGCAAGTTTCAGACTGCACTGCTGAAGGTCTCAAGTGTTGCCTACTTTTCTCAGAGATGTCACCAGAA
CTTGTGGGCGAGAAAATAGAGCCCGAAAGAGTATATGATTCAGTAAATGTCATACTTTCTTTACAGAGCAAAAATGGTGGTTTAGCAGG
ATGGGAACCAGCAGGAGCCTTTTCATGGTTGGAGCTGCTCAATCCAGTGGAGTTTCTGGAAGACGTTGTCATCGAATATGAGTATGTT
GAATGTACTTCATCGGCAATCCAAGCTATTCTTCTGTTTCGAAAGCTATATCCTCATCATAGGAAGAAAGAGATCGATAGCTTTATTGCA
AAAGCAGTACAGTACCTCCTAGATGAACAAAAGGATGATGGTTCTTGGTATGGAGAATGGGGAATCTGCTTCATATATGGAACATGGT
TTGCACTCAGAGGATTGGTGGCTGGCGGAAAGACTTACAAGAATTGTTTGACAATACGAAAAGCTGTAGAGTTTCTACTAAAAACACA
GACAGATGATGGAGGTTGGGGAGAGAGTTACCTATCCTGCCCAAAAAAAGTTTTTTGGCCTCTTGAAGGAAACAGATCAAATTTAGTA
CATACTGCATTCGGTTTAATGGGTCTAATTTATGCTGGACAGGCTGAAAGAGATCCCACGCCCATTCATCGTGCTGCAAAGCTATTGAT
CAATTCTCAAACGGAAACTGGTGATTTTCCTCAGCAGGAATTGATGGGAGTTTTCATGAAAACTTGCATGTTACACTATGGAGCATACA
GAAATGTGTTCCCATTATGGGCTCTTGCAGAATACCAAAAGTGA

> OSC4 / (3*S*,13*S*)-Malabarica-17,21-diene-3β,14-diol synthase (MDDS) (scaffold-BLAJ-2025975-Hemerocallis_spp)

ATGTGGAAGCTTAGGATTGCTGAAGGGGGGCCAGGTGTGAAGAGTCGCTATAATTTCATAGGAAGGCAAGTCTGGGAGTTTGAGGAA
AAACTTGGATCATCCGAAGAAGAAGAAGAAGAAGAGAAACTGGCAGTAGAAAAAGCTAGACACGAATTTCGGAGGAACAGGTTCCAG
AAGAAACAAGCATCGGATCTGCTTATGCGAATGCAGTTTGCTAAAGAGAAAACTTGTGATACAAATCTGTCGCAAGTAAAACTGGAGG
AAGAGCAGGAAGTTTCTCAAGTAGAAGTTACAATAACATTGCGAAGAGCAATCAACTATTTCTCAACCATTCAGGCACATGACGGGCAT
TGGCCAGCCGACTTTCCAGGGCCCTTATTTCTAACTCCAACCTTGATCATAGTTTTATATGTTACCCAAGCATTAGACACCATTCTGTCA
GCAGAACACCGGAAGGAGTTTTCCCGCTACCTATACAACCATCAGAATGAAGATGGAGGTTGGGGATTCCACACAGAGGGTCCCAGC
GTAATGTTTAGTACTGCTCTCTGCTACATAGCATTGAGACTACTCGGTGAGGATGTTGATGGTGGTGAAGATGGTGTTCTAACAAAAG
GACGAACATGGATTCTTGAACGTGGTGGAGTAACAGCTATACCTACTTGGGGGAAATTGTGGCTTTCGATACTAGGAGTGTTCGAATG
GTCAGGAGTAAACCCCATTCCACCAGAGCTCTTTATCTTTCCTACCCTTCTTCCTATTCATCCAGGTAGATTTTGGTGTCACTTTAGACT
GGCTTATCTACCAATGTCTTACTTGTATGGAAAGAAGTTTGTTGGTCCAGTTACAAACATTGTTCTATCGCTGAGAGAAGAGCTGCATA
GTCATCCTTACCATAAGATTGACTGGAATGCAGCACGTAAATTGTGTGCAAAGGAAGATCTCTACTATCCACATCCTTTAGCACAAGAT
ATCATATTGGAATGTCTGCATAGACTTGGTGAACCTCTTCTAAAGCACTGGCCTTTTTCAAGTTTGAGAAAGAAGGCACTACGAGACAT
AATGCAGTACATACACTATGAAGATGAAAATTCTGTATATCTCTGCGTTGGAGCCGGGCAAAAGGTATTATGTATGCTCTGCAGTTGGG
TAGAAGATCCAAGTTCAGATGCCTTCAAGCTGCATCTTGCAAGAATCCCTGATTATTTATGGGTTGCTGAAGATGGCATGAAAATGCAG
GGTTGTGGAAGTCAGCTCTGGGATGCTGCACTAGCAGTCCAAGCAATTTTGTCAAGCAACATCATAGAGGAGTATAGCACTAGTCTAC
TGAAAGCACATGAATTCATAAAATTTTCGCAGATTCTAGAGAATCCTTCTGGCAACTTCAATCGTTGGCATCGCCATATGTCTAAAGGT
GGATGGGCTTTCACAATTCCAGATCACGGGTGGCCAGTATCTGACTGCACAGCAGAGGCTCTTAAGGCGGCACTACTGTTATCAAAG
ATTTCACCAAATATAGTAGGCCAACCAATGGCAACTGAGCAGCTGTTTGATGCTCTACATATTATTCTCTCTTTACAGAATAGAAATGGG
GGCTTCTCTACTTGGGAGTTGGCACGTACATATCAATGGATGGAGTTTTTCAATGCCTCGGAGTTTTTTGCAGACATTGTTCTTGAGCA
TCAGTATGTGGAATGCACTTCATCTGCAGTCCAGGCACTGGCAATATTTAAGGAAATTTATCCTGGGCACCGCACGGAAGAAATAGAA
CAATGCATGGAAAAGGCAATGAAATTTATTGAAAATACACAAAAAGATGATGGTTCATGGTATGGCTCTTGGGGTATATGCTACACTTA
TGGCACATGGTTCGGAGTAGAAGGTCTTCTAGCTTGTGGAAAGACCTACAACACAAGTTCCAGGATCCGCAAGGCCTGCCAGTTTTTG
TTATCAAAGCAATTAAACTGTGGTGGGTGGGGAGAGAGTTATCTTTCATCCAAAAATCAGGCGTACACAAATCTAGATGGCAACCAAC
CTCATCTAGTAAATACTGCATGGGCAATGCTTGCTCTTATGAAAGCAGGCCAGGTTGAGAGAGATCCTACACCATTGAATCGTGCAGC
CAAATTCCTTATAAATATGCAAGAAGAAAATGGGGATTTTCCACAACAGGAAATGCTGGGAAGTCTTGGCAAAAATGGAGTTTTGAACT
ATGCAGCATACAGGAACATATTTCCGATATGGGCACTTGGAGAGTATCGAAAACATCTTCTCTGA

> OSC5 / 19-*epi*-Lupeol synthase (19ELS) (scaffold-JVBR-2009111-Aloe_vera)

ATGTGGAAGCTTAAGATTGCTGAAGGAGGTCCGGATGTCATGACTCGCTCTAATTTCATAGGAAGACAATTCTGGGAATTTGATCCAG
AACTTGGTTCTCGTGAAGAGCGTGAGGCAGTTGAAAGAGCTCGAGAAGAATTTGGAAGGAATAGGTTCCAAAAGAAAATACCATCAGA
TCTGCTTATGCGAATGCAGTTTGCAAGAGAGAATCCTTGTGATACAAAGCTACCACAAGTGAAACTGAATGAAAAGGAGGAAATTTCTG
TAGAGGCAGTCACAATAACATTGAGAAGAGCAATCAGCTATTTCTCATCCATTCAGGGACATGACGGACACTGGCCAGGCGATGCTG
GATGCTTACTGTATCTAACTCCAAGCTTGGTCATAGCTTTATATATCACTGGAGCACTAGACAGAACTTTGTCAGCTGAGCATCAGAAA
GAGATGAGACGCTACCTATACAATCATCAGAATGAAGATGGAGGCTGGGGGTTCCACGAAGAAGGCCACAGTGTCATGTTTAGCACT
GCTCTCTGCTACATAACATTAAGGTTACTAGGTGAGAAGATCAACGGCGGTGAAGATGGTGCTATGGTGAAAGCGCGTACATGGATTC
GTGACCGTGGTGGAGTAACATCTATACCTACTTGGGGGAAATTCTGGCTTTCGGTGCTAGGAATCATTGAGTGGGCGGGAATAAATCC
TATTCAACCAGAGTTCTTTATGCTTCCTTCCTTTCTTCCCATTCATCCAGGAAAACTTTGGTGTCATCTCAGACTGGTTTATCTTGGAAT
GTCTTACTTATATGGGAAGAAGTTTGTAGCTCCAGTTACAAATGTAATTTTGTCACTGAGAGAAGAGTTACATATTCAGCCTTACCACAT
GGTAGACTGGAATCTAGCACGCAAGTCATTCGCAAAGGAAGATGTCTATTATCCACATCCTTTAGCACAAGATATCATATGGGAATTCC
TCTATAGAATTGCCGAACCTCTTTTCACACGCTGGCCCCTTTCGACTTTGAGAGAGAAGGCTCTTCAAGCCGTTATAGAACAACTACAC
TACGAAGATGAAAATTCTCTTTATCACTGTGTATCATGTGTTGAAAAGATGTTAGCTATGCTTTGTTGTTGGGTGGAAGATCCAAGTTCA
GATGCATTCAAGCTGCACCTTGCAAGAATCCCTGATTATTTATGGGTTGCGGAGGACGGCGTGAAAATGCGGGGCGTTGGAAGTCAG
ATTTGGGATGCTGCATTGGCAGTCCAAGCAATTATGTCAAGCATGCTTGTAGAGGAATATGGGACTACTCTAAAGAAAGCACATGATTT
CATAAAGCTATCACAGAATCTGGAGAACAATTCTGGTGACTACAGCCGTCGGTATCGTCATATGTCTAAAGGTGGGTGGTCCTTTACA
ATGCCTGATAACAGATGGCCAGTATCTGACTGCACTGCGGAGGCTTTTAAGGCTGCACTTCTGCTATCAAAGCTCTCACCAAATATAG

TGGGTCAGTCTATGACAATTGAGAGGATGTGTGATGCTGTAAAATCTATTCTCTCTTTTCAGAACAAAAATGGGGGCTTCTCTGCATGG
GAGTTGACACGCACCTACGAGTGGCTTGAGTTTTGCAATGCTTCAGAGTTCTTTGCAGCCATCGTTGTCGATTACCAGTTTGTGGAAT
GCACTTCGTCAGCTGTTCAGGCATTGGTATTATTCAAGGAAATGTATCCTGGATATCTCAAGGAAGAAATAGAAATATGCGTTAAAAGG
GCAATGAAATATATTGAAAGTACACAGAAGGAAGATGGTTCATGGTATGGCTCTTGGGGAATATGCTTCACTTACGCAACATGGTTTGG
CATAGAAGGCCTTTTAGCTTGTGGAAAGACCTACGATACAAGTTCCAGAATCCGCAAGGCTTGTCAATTTCTGTTGTCAAAGCAGTTAG
ACTCAGGTGGTTGGGGGGAGAGTTATCTTTCATCCACTACTGAGGTATATACAAATCTAGAGGGCAATCGATCGCATCTAGTAAATAC
ATCATGGGCAATGCTTGCTCTTATCAAAGCTGGCCAGGTCGAGAGGGATCCTGAACCTATGCATCGTGCAGCAAAATTCCTCATAAAT
ATGCAAGAAGAAAATGGGGATTTTCCACAACAGGAAATTCTGGGGATTTTCGCCAAAAATGCTGGTCTCAACTTTGCACAGTACAGAA
GCATATTCCCAATTTGGGCTCTTGGAGAGTATCGAAAGCGTCTTCTATGA

> OSC6 / Protostahopenol synthase (PHS) (scaffold-CLMX-2046562-Escallonia_rubra)

ATGTGGAAGCTTATACACTCGGAGGGCAGGGATGACCCTGAGCTTAAAAGCTATAATAATCATCTTGGTAGACAGTTCTGGGAATTCC
ACCCTGATCTCGGGACGCCCGAGGAACGAGCTCAAATCGAAAAACTCCGTGAGGAGTTTACTAAGAACCGATTCCAAGTTAAAGAAA
GTTCTGATCTTCTTTTTAGATTGCAGTTTGCAAAAGAACATCCCTTGGAGAAGAAGTTACCGCCACAGGTCAAGGTGGGAAGTTCGGA
GGATGTGGGCGAGGAAGCAGTCGAAACCACATTGAGGAGGGCTTTAAGATACTTCTCAACACTTCAGACTGACGATGGCTTCTGGCC
TGGTGATTATGCCGGCATTTTGTTCCTTCTCCCTGGCCTTGTTATCGCTCTGTCTGTAACAGGGGCCTTAGATGCAAGTTTGTCGCAAG
AACACCAAAGGGAACTATGTCGTTACATTTATAACCATCAGAATGTAGATGGAGGTTGGGGATTTCACATAGAAGGACCGAGCAGCAT
GTTCAGTACAGCCCTTAATTATGTGGCTATGAGGTTGCTGGGAGAAAAGATGGATGGTGGAGATGGGGCCATGGAGAAAGCAAGAAG
ATGGATTCTTGACCATGGTGGCGCTACCGGCATTACATCTTGGGGAAAAATGTGGCTCTCGGTACTCGGGGTTTATGAGTGGAGCGG
CAACAATCCTATCCCACCAGAGATATGGCTTCTTCCTCACTTTCTTCCCATACATCCAGGGCGTATGTGGTGCCATTGCCGAATGGTGT
ATCTCCCTATGTCATATCTTTATGGGAAAAGATTTGTAGGGCCCGTGGACTCTACAATTTTATCACTACGAGGAGAACTTTATACACGT
CCATATAATGAGATTAACTGGGACATGGCAAAGAATGAATGTGCCAAGGAAGATCTATTTTACCCACATTTGCTCGGACAAGAGTTGGT
TTTAAATGGTTTGCACAAGTTTGTAGAACCTCTTCTAATGACTTGGCCTTTTTCGAAACTGCGAAAGAAGGCACTGAGCACTGCGATGC
AACATATCGAGTATGAGGACGAAAGCACTCAATATTTATGCATCGGACCTGTAAATAAGGCACTAAATATGATATGTCGTTGGATAGAC
GATCCGAATTCAAGGGCGAATAAGTTACACCTTTCAAGGGTAAAAGATTATCTATGGGTGGCTGAAGATGGCATGAAGTATCAGGCGT
ACAATGGATCTCAGCTATGGGATGTTATATTTGCTGTTCAAGCAATACTTGGGACGAAGCTCAGCGACGAATACGGTTCAGTACTTAAA
AGAGCAAATGAATTCATCAAAGGCTCACAATTTAAAATCAATAGTTCAGCTGATTTTAGTCAATGGTACCGTGACAATACAATTGGTGGA
TGGAGCTTTAGTACAGTGGATCAAGGTTGGATTGTAACCGATTGCACCGGAGAATGTCTGAAGATATCAATGTTGCTATCACAAATGC
CATCGGATGTTGTTGGTGATACATTGGCACCAGAAGGTTTATATAATGCCGTTAATTTGCTTCTACCACTTCAGAACAGCAATGGAGGT
TTTGGTTCATATGAGCTCGCAAGGTCCTATCCGTGGTTAGAGATGATCAATCCAGCTGAAACATTCGGGGGTATCATGATTGATTACCA
GACCGTAGAGTGCACCTCTTCAGTAACTCAAGGACTTTCTTTATTCAAGAAATTATATCCAGATCACAGGAGCGATGAAATAGAATCAT
GCATCAGAAAAGCACTCGAGTTCATCGAGAGCGTGCAATTGCCGGATGGTTCATGGTTTGGGACTTGGGGAGTATGCTACACATACG
GGACATGGTTCGGAATCAAAGGCTTGGTAAATGGAGGCAAGACGTACCAAAGTAGCAGTAGCATTCGAAGGGCTTGTGATTTCCTTCT
ATCCAAACAACTTGATTCCGGTGGTTGGGGAGAAAATTACACCTCTGGCCAAGACAAGGTGTATAAAAATCTACAAGGGAACAAATCA
CATATAGTGAACACTGCTTGGGCTATGCTGGGTCTCATTGAGGCTGGGCAGGGCAAAAGAGATCCGACCCCATTGCATCGTGCTGCC
AAGGTTTTAATAAATCACCAATTGGAAAATGGAGACTTCCCTGAACAGGAACTTGTGGGAGTTTTTAATAGGAACAGTATGATAACCTA
TGCTTCATACAGAAATGTCTTCCCTATCTGGGCTCTTGGAAAGTATCTCAATGATGTGTTAATATGA

Table S12. Sequences of the primers used in this study.

Start and stop codons are marked in red. BsaI restriction sites for Golden Gate cloning are marked in green, and the cutting site is indicated with a slash. Mutagenesis sites are highlighted in yellow.

Primer name	Primer sequence	Vector	Purpose
HsOSC_Fw	TTGGTCTCA/AAAAATGTGGAAGCTTAGGATTGCTGAAGG	pHREAC	Cloning MDDS into pHREAC
HsOSC_Rv	TTGGTCTCT/AGCGTCAGAGAAGATGTTTTCGATACTCTCCA	pHREAC	Cloning MDDS into pHREAC
HsF122N_Fw	TTGGTCTCA/CAACCCAGGGCCCTTATTTCTAACTCCAACC	pHREAC	Mutagenesis MDDS-F122N
HsF122N_Rv	TTGGTCTCT/GTTGTCGGCTGGCCAATGCCCGT	pHREAC	Mutagenesis MDDS-F122N
HsF122S_Fw	TTGGTCTCA/CAGCCCAGGGCCCTTATTTCTAACTCCAACC	pHREAC	Mutagenesis MDDS-F122S
HsF122S_Rv	TTGGTCTCT/GCTGTCGGCTGGCCAATGCCCGT	pHREAC	Mutagenesis MDDS-F122S
HsF122V_Fw	TTGGTCTCA/CGTGCCAGGGCCCTTATTTCTAACTCCAACC	pHREAC	Mutagenesis MDDS-F122V
HsF122V_Rv	TTGGTCTCT/CACGTCGGCTGGCCAATGCCCGT	pHREAC	Mutagenesis MDDS-F122V
HsF122Y_Fw	TTGGTCTCA/CTATCCAGGGCCCTTATTTCTAACTCCAACC	pHREAC	Mutagenesis MDDS-F122Y
HsF122Y_Rv	TTGGTCTCT/ATAGTCGGCTGGCCAATGCCCGT	pHREAC	Mutagenesis MDDS-F122Y
HsH262Y_Fw	TTGGTCTCA/GTTATTTTAGACTGGCTTATCTACCAATGTCT	pHREAC	Mutagenesis MDDS-H262Y
HsH262Y_Rv	TTGGTCTCT/TAACACCAAAATCTACCTGGATGAATAGGAAGAA	pHREAC	Mutagenesis MDDS-H262Y
HsAGQ372_374PVN_Fw	TTGGTCTCA/GAACAAGGTATTATGTATGCTCTGCAGTTGG	pHREAC	Mutagenesis MDDS-AGQ372-374PVN
HsAGQ372_374PVN_Rv	TTGGTCTCT/GTTCACCGGTCCAACGCAGAGATATACAGAATTTTCATC	pHREAC	Mutagenesis MDDS-AGQ372-374PVN
HsAGQ372_374SVP_Fw	TTGGTCTCA/TGCCGAAGGTATTATGTATGCTCTGCAGTTGG	pHREAC	Mutagenesis MDDS-AGQ372-374SVP
HsAGQ372_374SVP_Rv	TTGGTCTCT/GGCACGCTTCCAACGCAGAGATATACAGAATTTTCATC	pHREAC	Mutagenesis MDDS-AGQ372-374SVP
HsC415F_Fw	TTGGTCTCA/GTTTTGGAAGTCAGCTCTGGGATGCTGC	pHREAC	Mutagenesis MDDS-C415F
HsC415F_Rv	TTGGTCTCT/AAACCCTGCATTTTCATGCCATCTTCAG	pHREAC	Mutagenesis MDDS-C415F
HsC415Y_Fw	TTGGTCTCA/GTTATGGAAGTCAGCTCTGGGATGCTGC	pHREAC	Mutagenesis MDDS-C415Y
HsC415Y_Rv	TTGGTCTCT/TAACCCTGCATTTTCATGCCATCTTCAG	pHREAC	Mutagenesis MDDS-C415Y
HsGC414_415SF_Fw	TTGGTCTCA/CTTTGGAAGTCAGCTCTGGGATGCTGC	pHREAC	Mutagenesis MDDS-G414S/C415F
HsGC414_415SF_Rv	TTGGTCTCC/AAAGGACTGCATTTTCATGCCATCTT	pHREAC	Mutagenesis MDDS-G414S/C415F
HsS729V_Rv	TTGGTCTCT/AGCGTCAGAGAAGATGTTTTCGATACTCTCCAAGTGCCCATATCGGAAATATGTTCCTGTATGCTGCATAGTTCAAAACTCCATTTTTGCCAAGCACTCCCAGCATTTCCT	pHREAC	Mutagenesis MDDS-S729V (in combination with HsOSC_Fw)
HsL730F_Rv	TTGGTCTCT/AGCGTCAGAGAAGATGTTTTCGATACTCTCCAAGTGCCCATATCGGAAATATGTTCCTGTATGCTGCATAGTTCAAAACTCCATTTTTGCCAAAACTTCCCAGCATTTCCT	pHREAC	Mutagenesis MDDS-L730F (in combination with HsOSC_Fw)
HsSL729_730VF_Rv	TTGGTCTCT/AGCGTCAGAGAAGATGTTTTCGATACTCTCCAAGTGCCCATATCGGAAATATGTTCCTGTATGCTGCATAGTTCAAAACTCCATTTTTGCCAAACACTCCCAGCATTTCCT	pHREAC	Mutagenesis MDDS-S729V/L730F (in combination with HsOSC_Fw)
AvOSC_DomFw	TTGGTCTCA/AAAAATGTGGAAGCTTAAGATTGCTGAAGGA	pHREAC	Cloning 19ELS into pHREAC
AvOSC_DomRv	TTGGTCTCT/AGCGTCATAGAAGACGCTTTCGATACTCTCCAAGAGCCCAAATTGGGAA TATGCTTCTGTACTGTGCAAAGTTCAGACCAGCATTTTTGGCGAAAATCCCCA	pHREAC	Cloning 19ELS into pHREAC
AvOSC_CloRv	TTGGTCTCT/AGCGTCATAGAAGACGCTTTCGATACTCT	pHREAC	Cloning 19ELS into pHREAC
AvC119G_Fw	TTGGTCTCA/AGGCTTACTGTATCTAACTCCAAGCTTGGTC	pHREAC	Mutagenesis 19ELS-C119G
AvC119G_Rv	TTGGTCTCT/GCCTCCAGCATCGCCTGGCCAGT	pHREAC	Mutagenesis 19ELS-C119G
AvL120P_Fw	CTGGATGCCCACTGTATCTAAC	pEAQ	Mutagenesis 19ELS-L120P
AvL120P_Rv	GTTAGATACAGTGGGCATCCAG	pEAQ	Mutagenesis 19ELS-L120P
Av119_120_Fw	TTGGTCTCA/AGGCCCGCTGTATCTAACTCCAAGCTTGGTCATA	pHREAC	Mutagenesis 19ELS-C119G/L120P
Av119_120_Rv	TTGGTCTCG/GGCCTCCAGCATCGCCTGGCCAGTGTCCGT	pHREAC	Mutagenesis 19ELS-C119G/L120P
AvV365L_Fw	TTGGTCTCA/TCTGTCATGTGTTGAAAAGATGTTAGCTATG	pHREAC	Mutagenesis 19ELS-V365L
AvV365L_Rv	TTGGTCTCT/CAGACAGTGATAAAGAGAATTTTCATCTTCGTAGTG	pHREAC	Mutagenesis 19ELS-V365L
AvS366G_Fw	TTGGTCTCA/AGGCTGTGTTGAAAAGATGTTAGCTATGCTT	pHREAC	Mutagenesis 19ELS-S366G
AvS366G_Rv	TTGGTCTCT/GCCTACACAGTGATAAAGAGAATTTTCATCTTCGTA	pHREAC	Mutagenesis 19ELS-S366G
AvVS365_366LG_Fw	TTGGTCTCA/TGGGCTGTGTTGAAAAGATGTTAGCTATGCTT	pHREAC	Mutagenesis 19ELS-V365L/S366G
AvVS365_366LG_Rv	TTGGTCTCG/CCCAGACAGTGATAAAGAGAATTTTCATCTTCGTAGT	pHREAC	Mutagenesis 19ELS-V365L/S366G
AvG409S_Fw	TTGGTCTCA/GAGCGTTGGAAGTCAGATTTGGGATGCTGC	pHREAC	Mutagenesis 19ELS-G409S
AvG409S_Rv	TTGGTCTCT/GCTCCGCATTTTCACGCCGTCCTCC	pHREAC	Mutagenesis 19ELS-G409S
AvV410F_Fw	TTGGTCTCA/CTTTGGAAGTCAGATTTGGGATGCTGCATTG	pHREAC	Mutagenesis 19ELS-V410F
AvV410F_Rv	TTGGTCTCT/AAAGCCCCGCATTTTCACGCCGTCC	pHREAC	Mutagenesis 19ELS-V410F
AvGV409_410SF_Fw	TTGGTCTCA/CTTTGGAAGTCAGATTTGGGATGCTGCATTG	pHREAC	Mutagenesis 19ELS-G409S/V410F
AvGV409_410SF_Rv	TTGGTCTCC/AAAGCTCCGCATTTTCACGCCGTCCTCCGC	pHREAC	Mutagenesis 19ELS-G409S/V410F
ErOSC_Fw1	TTGGTCTCA/AAAAATGTGGAAGCTTATACACTCGGAGGG	pHREAC	Cloning PHS into pHREAC
ErOSC_Rv1	TTGGTCTCT/CAGACCCAGCATAGCCCAAGCAGTGT	pHREAC	Cloning PHS into pHREAC
ErOSC_Fw2	TTGGTCTCA/TCTGATTGAGGCTGGGCAGGGCAAAAG	pHREAC	Cloning PHS into pHREAC

ErOSC_Rv2	TTGGTCTCT/AGCGTCATATTAACACATCATTGAGATACTTTCCAAGAG	pHREAC	Cloning PHS into pHREAC
ErH259F_Fw	TTGGTCTCA/CTTTTGCCGAATGGTGTATCTCCCTATGTCA	pHREAC	Mutagenesis PHS-H259F
ErH259F_Rv	TTGGTCTCT/AAAGCACCACATACGCCCTGGATGTATGG	pHREAC	Mutagenesis PHS-H259F
ErF474L_Fw	TTGGTCTCA/CCTGAGTACAGTGGATCAAGGTTGGATTGTA	pHREAC	Mutagenesis PHS-F474L
ErF474L_Rv	TTGGTCTCT/CAGGCTCCATCCACCAATTGTATTGTCACGGTA	pHREAC	Mutagenesis PHS-F474L
ErS533A_Fw	TTGGTCTCA/TGCGTATGAGCTCGCAAGGTCCTATCCG	pHREAC	Mutagenesis PHS-S533A
ErS533A_Rv	TTGGTCTCT/CGCACCAAAACCTCCATTGCTGTTCTGAAGT	pHREAC	Mutagenesis PHS-S533A
ErF728A_Rv	TTGGTCTCT/AGCGTCATATTAACACATCATTGAGATACTTTCCAAGAGCCCAGATAGGGAAGACATTTCTGTATGAAGCATAGGTTATCATACTGTTCCTATTCGCAACTCCCACAAGTT	pHREAC	Mutagenesis PHS-F728A (in combination with ErOSC_Fw1)
ErF728S_Rv	TTGGTCTCT/AGCGTCATATTAACACATCATTGAGATACTTTCCAAGAGCCCAGATAGGGAAG ACATTTCTGTATGAAGCATAGGTTATCATACTGTTCCTATTGCTAACTCCCACAAGTT	pHREAC	Mutagenesis PHS-F728A (in combination with ErOSC_Fw1)
ErI734F_Rv	TTGGTCTCT/AGCGTCATATTAACACATCATTGAGATACTTTCCAAGAGCCCAGATAGGGAAGACATTTCTGTATGAAGCATAGGTAAACATACTGTTCCTATTAAAAACTCCCACAAGTT	pHREAC	Mutagenesis PHS-I734F (in combination with ErOSC_Fw1)
rLC39-pEAQ-910F	CAACGTTGTCAGATCGTGCTTC	pEAQ	Sequencing, PCR and colony PCR
rLC54-pEAQ-3UR	TCCCTTCAGCAAGGACACAAAAAG	pEAQ	Sequencing, PCR and colony PCR
rLC42_pHREAC_5292F	CTGTCACTTTATTGAGAAGATAGTGG	pHREAC	Sequencing and colony PCR
rLC59_pHREAC_5828R	CCTTGCTGAAGGGACGACCTG	pHREAC	Sequencing and colony PCR
ShOSC-SeqRv	CCACCCATTATCTGCTGTTGTG	pEAQ	Sequencing
DbOSC-SeqFw	AGCCGCATTAAAGAGAGCTGT	pEAQ	Sequencing
FaOSC-SeqRv	CAGTGCAGTCTGAAACTTGCC	pEAQ	Sequencing
HsOSC-SeqRv	CTTTGATAACAGTAGTGCCGCC	pEAQ/ pHREAC	Sequencing
AvOSC-SeqFw	AGCACTGCTCTCTGCTACAT	pEAQ	Sequencing
AvOSC-SeqRv	GCCATACCATGAACCATCTTCC	pEAQ	Sequencing
AvOSC_SeqRv2	CAACTCCCATGCAGAGAAGC	pHREAC	Sequencing
ErOSC-SeqRv	CGGTGCAATCGGTTACAATC	pEAQ/ pHREAC	Sequencing

Supplementary References

(1) Xu, Z.; Godber, J. S. Purification and Identification of Components of γ-Oryzanol in Rice Bran Oil. *J. Agric. Food Chem.* **1999**, *47* (7), 2724–2728. https://doi.org/10.1021/jf981175j.

(2) Carpenter, E. J.; Matasci, N.; Ayyampalayam, S.; Wu, S.; Sun, J.; Yu, J.; Jimenez Vieira, F. R.; Bowler, C.; Dorrell, R. G.; Gitzendanner, M. A.; Li, L.; Du, W.; K. Ullrich, K.; Wickett, N. J.; Barkmann, T. J.; Barker, M. S.; Leebens-Mack, J. H.; Wong, G. K.-S. Access to RNA-Sequencing Data from 1,173 Plant Species: The 1000 Plant Transcriptomes Initiative (1KP). *GigaScience* **2019**, *8* (10), giz126. https://doi.org/10.1093/gigascience/giz126.

(3) Chen, K.; Zhang, M.; Ye, M.; Qiao, X. Site-Directed Mutagenesis and Substrate Compatibility to Reveal the Structure–Function Relationships of Plant Oxidosqualene Cyclases. *Nat. Prod. Rep.* **2021**, *38* (12), 2261–2275. https://doi.org/10.1039/D1NP00015B.

(4) Gerlt, J. A.; Bouvier, J. T.; Davidson, D. B.; Imker, H. J.; Sadkhin, B.; Slater, D. R.; Whalen, K. L. Enzyme Function Initiative-Enzyme Similarity Tool (EFI-EST): A Web Tool for Generating Protein Sequence Similarity Networks. *Biochim. Biophys. Acta BBA - Proteins Proteomics* **2015**, *1854* (8), 1019–1037. https://doi.org/10.1016/j.bbapap.2015.04.015.

(5) Zallot, R.; Oberg, N.; Gerlt, J. A. The EFI Web Resource for Genomic Enzymology Tools: Leveraging Protein, Genome, and Metagenome Databases to Discover Novel Enzymes and Metabolic Pathways. *Biochemistry* **2019**, *58* (41), 4169–4182. https://doi.org/10.1021/acs.biochem.9b00735.

(6) Oberg, N.; Zallot, R.; Gerlt, J. A. EFI-EST, EFI-GNT, and EFI-CGFP: Enzyme Function Initiative (EFI) Web Resource for Genomic Enzymology Tools. *J. Mol. Biol.* **2023**, *435* (14), 168018. https://doi.org/10.1016/j.jmb.2023.168018.

(7) Shannon, P.; Markiel, A.; Ozier, O.; Baliga, N. S.; Wang, J. T.; Ramage, D.; Amin, N.; Schwikowski, B.; Ideker, T. Cytoscape: A Software Environment for Integrated Models of Biomolecular Interaction Networks. *Genome Res.* **2003**, *13* (11), 2498–2504. https://doi.org/10.1101/gr.1239303.

(8) Bally, J.; Jung, H.; Mortimer, C.; Naim, F.; Philips, J. G.; Hellens, R.; Bombarely, A.; Goodin, M. M.; Waterhouse, P. M. The Rise and Rise of *Nicotiana Benthamiana*: A Plant for All Reasons. *Annu. Rev. Phytopathol.* **2018**, *56* (1), 405–426. https://doi.org/10.1146/annurev-phyto-080417-050141.

(9) Chuang, L.; Franke, J. Rapid Combinatorial Coexpression of Biosynthetic Genes by Transient Expression in the Plant Host Nicotiana Benthamiana. In *Engineering Natural Product Biosynthesis: Methods and Protocols*; Skellam, E., Ed.; Methods in Molecular Biology; Springer US: New York, NY, 2022; pp 395–420. https://doi.org/10.1007/978-1-0716-2273-5_20.

(10) Liang, M.; Zhang, F.; Xu, J.; Wang, X.; Wu, R.; Xue, Z. A Conserved Mechanism Affecting Hydride Shifting and Deprotonation in the Synthesis of Hopane Triterpenes as Compositions of Wax in Oat. *Proc. Natl. Acad. Sci. U. S. A.* **2022**, *119* (12), e2118709119. https://doi.org/10.1073/pnas.2118709119.

(11) Ito, R.; Mori, K.; Hashimoto, I.; Nakano, C.; Sato, T.; Hoshino, T. Triterpene Cyclases from Oryza Sativa L.: Cycloartenol, Parkeol and Achilleol B Synthases. *Org. Lett.* **2011**, *13* (10), 2678–2681. https://doi.org/10.1021/ol200777d.

(12) Sainsbury, F.; Thuenemann, E. C.; Lomonossoff, G. P. pEAQ: Versatile Expression Vectors for Easy and Quick Transient Expression of Heterologous Proteins in Plants. *Plant Biotechnol. J.* **2009**, *7* (7), 682–693. https://doi.org/10.1111/j.1467-7652.2009.00434.x.

(13) Peyret, H.; Brown, J. K. M.; Lomonossoff, G. P. Improving Plant Transient Expression through the Rational Design of Synthetic 5′ and 3′ Untranslated Regions. *Plant Methods* **2019**, *15* (1), 108. https://doi.org/10.1186/s13007-019-0494-9.

(14) Reed, J.; Stephenson, M. J.; Miettinen, K.; Brouwer, B.; Leveau, A.; Brett, P.; Goss, R. J. M.; Goossens, A.; O'Connell, M. A.; Osbourn, A. A Translational Synthetic Biology Platform for Rapid Access to Gram-Scale Quantities of Novel Drug-like Molecules. *Metab. Eng.* **2017**, *42*, 185–193. https://doi.org/10.1016/j.ymben.2017.06.012.

(15) Sheldrick, G. M. SHELXT – Integrated Space-Group and Crystal-Structure Determination. *Acta Crystallogr. Sect. Found. Adv.* **2015**, *71* (1), 3–8. https://doi.org/10.1107/S2053273314026370.

(16) Dolomanov, O. V.; Bourhis, L. J.; Gildea, R. J.; Howard, J. a. K.; Puschmann, H. OLEX2: A Complete Structure Solution, Refinement and Analysis Program. *J. Appl. Crystallogr.* **2009**, *42* (2), 339–341. https://doi.org/10.1107/S0021889808042726.

(17) Sheldrick, G. M. Crystal Structure Refinement with SHELXL. *Acta Crystallogr. Sect. C Struct. Chem.* **2015**, *71* (1), 3–8. https://doi.org/10.1107/S2053229614024218.

(18) Thoma, R.; Schulz-Gasch, T.; D'Arcy, B.; Benz, J.; Aebi, J.; Dehmlow, H.; Hennig, M.; Stihle, M.; Ruf, A. Insight into Steroid Scaffold Formation from the Structure of Human Oxidosqualene Cyclase. *Nature* **2004**, *432* (7013), 118–122. https://doi.org/10.1038/nature02993.

(19) Guindon, S.; Dufayard, J.-F.; Lefort, V.; Anisimova, M.; Hordijk, W.; Gascuel, O. New Algorithms and Methods to Estimate Maximum-Likelihood Phylogenies: Assessing the Performance of PhyML 3.0. *Syst. Biol.* **2010**, *59* (3), 307–321. https://doi.org/10.1093/sysbio/syq010.

(20) Jumper, J.; Evans, R.; Pritzel, A.; Green, T.; Figurnov, M.; Ronneberger, O.; Tunyasuvunakool, K.; Bates, R.; Žídek, A.; Potapenko, A.; Bridgland, A.; Meyer, C.; Kohl, S. A. A.; Ballard, A. J.; Cowie, A.; Romera-Paredes, B.; Nikolov, S.; Jain, R.; Adler, J.; Back, T.; Petersen, S.; Reiman, D.; Clancy, E.; Zielinski, M.; Steinegger, M.; Pacholska, M.; Berghammer, T.; Bodenstein, S.; Silver, D.; Vinyals, O.; Senior, A. W.; Kavukcuoglu, K.; Kohli, P.; Hassabis, D. Highly Accurate Protein Structure Prediction with AlphaFold. *Nature* **2021**, *596* (7873), 583–589. https://doi.org/10.1038/s41586-021-03819-2.

(21) Eberhardt, J.; Santos-Martins, D.; Tillack, A. F.; Forli, S. AutoDock Vina 1.2.0: New Docking Methods, Expanded Force Field, and Python Bindings. *J. Chem. Inf. Model.* **2021**, *61* (8), 3891–3898. https://doi.org/10.1021/acs.jcim.1c00203.

(22) Trott, O.; Olson, A. J. AutoDock Vina: Improving the Speed and Accuracy of Docking with a New Scoring Function, Efficient Optimization, and Multithreading. *J. Comput. Chem.* **2010**, *31* (2), 455–461. https://doi.org/10.1002/jcc.21334.

(23) Meng, E. C.; Goddard, T. D.; Pettersen, E. F.; Couch, G. S.; Pearson, Z. J.; Morris, J. H.; Ferrin, T. E. UCSF ChimeraX: Tools for Structure Building and Analysis. *Protein Sci.* **2023**, *32* (11), e4792. https://doi.org/10.1002/pro.4792.

(24) Langmead, B.; Salzberg, S. L. Fast Gapped-Read Alignment with Bowtie 2. *Nat. Methods* **2012**, *9* (4), 357–359. https://doi.org/10.1038/nmeth.1923.

(25) Li, H.; Handsaker, B.; Wysoker, A.; Fennell, T.; Ruan, J.; Homer, N.; Marth, G.; Abecasis, G.; Durbin, R.; 1000 Genome Project Data Processing Subgroup. The Sequence Alignment/Map Format and SAMtools. *Bioinformatics* **2009**, *25* (16), 2078–2079. https://doi.org/10.1093/bioinformatics/btp352.

(26) Robinson, J. T.; Thorvaldsdóttir, H.; Winckler, W.; Guttman, M.; Lander, E. S.; Getz, G.; Mesirov, J. P. Integrative Genomics Viewer. *Nat. Biotechnol.* **2011**, *29* (1), 24–26. https://doi.org/10.1038/nbt.1754.

(27) Yang, C.; Halitschke, R.; O'Connor, S. E. OXIDOSQUALENE CYCLASE 1 and 2 Influence Triterpene Biosynthesis and Defense in Nicotiana Attenuata. *Plant Physiol.* **2024**, *194* (4), 2580–2599. https://doi.org/10.1093/plphys/kiad643.

(28) Tareen, A.; Kinney, J. B. Logomaker: Beautiful Sequence Logos in Python. *Bioinformatics* **2020**, *36* (7), 2272–2274. https://doi.org/10.1093/bioinformatics/btz921.

(29) Püllmann, P.; Ulpinnis, C.; Marillonnet, S.; Gruetzner, R.; Neumann, S.; Weissenborn, M. J. Golden Mutagenesis: An Efficient Multi-Site-Saturation Mutagenesis Approach by Golden Gate Cloning with Automated Primer Design. *Sci. Rep.* **2019**, *9* (1), 1–11. https://doi.org/10.1038/s41598-019-47376-1.

(30) Jamison, M. T.; Wang, X.; Cheng, T.; Molinski, T. F. Synergistic Anti-Candida Activity of Bengazole A in the Presence of Bengamide A. *Mar. Drugs* **2019**, *17* (2), 102. https://doi.org/10.3390/md17020102.

(31) Neelakandan, A. K.; Song, Z.; Wang, J.; Richards, M. H.; Wu, X.; Valliyodan, B.; Nguyen, H. T.; Nes, W. D. Cloning, Functional Expression and Phylogenetic Analysis of Plant Sterol 24C-Methyltransferases Involved in Sitosterol Biosynthesis. *Phytochemistry* **2009**, *70* (17), 1982–1998. https://doi.org/10.1016/j.phytochem.2009.09.003.

(32) Xue, Z.; Duan, L.; Liu, D.; Guo, J.; Ge, S.; Dicks, J.; ÓMáille, P.; Osbourn, A.; Qi, X. Divergent Evolution of Oxidosqualene Cyclases in Plants. *New Phytol.* **2012**, *193* (4), 1022–1038. https://doi.org/10.1111/j.1469-8137.2011.03997.x.

(33) Pearson, A.; Budin, M.; Brocks, J. J. Phylogenetic and Biochemical Evidence for Sterol Synthesis in the Bacterium Gemmata Obscuriglobus. *Proc. Natl. Acad. Sci. U. S. A.* **2003**, *100* (26), 15352–15357. https://doi.org/10.1073/pnas.2536559100.

(34) Banta, A. B.; Wei, J. H.; Gill, C. C. C.; Giner, J.-L.; Welander, P. V. Synthesis of Arborane Triterpenols by a Bacterial Oxidosqualene Cyclase. *Proc. Natl. Acad. Sci. U. S. A.* **2017**, *114* (2), 245–250. https://doi.org/10.1073/pnas.1617231114.

(35) Song, Z.; Chen, D.; Sui, S.; Wang, Y.; Cen, S.; Dai, J. Characterization of a Malabaricane-Type Triterpene Synthase from Astragalus Membranaceus and Enzymatic Synthesis of Astramalabaricosides. *J. Nat. Prod.* **2023**, *86* (7), 1815–1823. https://doi.org/10.1021/acs.jnatprod.3c00331.

(36) Hoshino, T.; Shimizu, K.; Sato, T. Deletion of the Gly600 Residue of Alicyclobacillus Acidocaldarius Squalene Cyclase Alters the Substrate Specificity into That of the Eukaryotic-Type Cyclase Specific to (3S)-2,3-Oxidosqualene. *Angew. Chem. Int. Ed.* **2004**, *43* (48), 6700–6703. https://doi.org/10.1002/anie.200461523.

(37) Ito, R.; Hashimoto, I.; Masukawa, Y.; Hoshino, T. Effect of Cation–π Interactions and Steric Bulk on the Catalytic Action of Oxidosqualene Cyclase: A Case Study of Phe728 of β-Amyrin Synthase from Euphorbia Tirucalli L. *Chem. – Eur. J.* **2013**, *19* (50), 17150–17158. https://doi.org/10.1002/chem.201301917.

(38) Gottlieb, H. E.; Kotlyar, V.; Nudelman, A. NMR Chemical Shifts of Common Laboratory Solvents as Trace Impurities. *J. Org. Chem.* **1997**, *62* (21), 7512–7515. https://doi.org/10.1021/jo971176v.

(39) Nazir, M.; Ahmad, W.; Kreiser, W. Isolation and NMR-Assignments of 19αH-Lupeol from E. Helioscopia Linn (N.O. Euphorbiaceae). *Biol. Sci. - PJSIR* **1998**, *41* (1), 6–10.

(40) Bianchini, J.-P.; Gaydou, E. M.; Rafaralahitsimba, G.; Waegell, B.; Zahra, J.-P. Dammarane Derivatives in the Fruit Lipids of Olea Madagascariensis. *Phytochemistry* **1988**, *27* (7), 2301–2304. https://doi.org/10.1016/0031-9422(88)80147-X.

(41) Fuchino, H.; Konishi, S.; Satoh, T.; Yagi, A.; Saitsu, K.; Tatsumi, T.; Tanaka, N. Chemical Evaluation of Betula Species in Japan. II. Constituents of Betula Platyphylla Var. Japonica. *Chem. Pharm. Bull. (Tokyo)* **1996**, *44* (5), 1033–1038. https://doi.org/10.1248/cpb.44.1033.

(42) Akihisa, T.; Arai, K.; Kimura, Y.; Koike, K.; Kokke, W. C. M. C.; Shibata, T.; Nikaido, T. Camelliols A−C, Three Novel Incompletely Cyclized Triterpene Alcohols from Sasanqua Oil (Camellia Sasanqua). *J. Nat. Prod.* **1999**, *62* (2), 265–268. https://doi.org/10.1021/np980336a.

(43) Lodeiro, S.; Xiong, Q.; Wilson, W. K.; Ivanova, Y.; Smith, M. L.; May, G. S.; Matsuda, S. P. T. Protostadienol Biosynthesis and Metabolism in the Pathogenic Fungus Aspergillus Fumigatus. *Org. Lett.* **2009**, *11* (6), 1241–1244. https://doi.org/10.1021/ol802696a.
